# Is there a health inequality in gambling related harms? A systematic review

**DOI:** 10.1186/s12889-021-10337-3

**Published:** 2021-02-06

**Authors:** Jodie N. Raybould, Michael Larkin, Richard J. Tunney

**Affiliations:** School of Psychology, Aston University, Birmingham, B7 4ET UK

**Keywords:** Gambling, Gambling disorder, Problem gambling, Pathological gambling, Harms, Gambling harms, Harms measures, Health inequality, Risk severity, Age, Gender, Culture, Gambling behaviour, Online gambling, Socioeconomic status

## Abstract

**Background:**

Here we present a systematic review of the existing research into gambling harms, in order to determine whether there are differences in the presentation of these across demographic groups such as age, gender, culture, and socioeconomic status, or gambling behaviour categories such as risk severity and participation frequency.

**Primary and secondary outcome measures:**

Inclusion criteria were: 1) focus on gambling harms; 2) focus on harms to the gambler rather than affected others; 3) discussion of specific listed harms and not just harms in general terms. Exclusion criteria were: 1) research of non-human subjects; 2) not written in English; 3) not an empirical study; 4) not available as a full article.

**Methods:**

We conducted a systematic search using the Web of Science and Scopus databases in August 2020. Assessment of quality took place using Standard Quality Assessment Criteria.

**Results:**

A total of 59 studies published between 1994 and 2020 met the inclusion criteria. These were categorised into thematic groups for comparison and discussion. There were replicated differences found in groups defined by age, socioeconomic status, education level, ethnicity and culture, risk severity, and gambling behaviours.

**Conclusion:**

Harms appear to be dependent on specific social, demographic and environmental conditions that suggests there is a health inequality in gambling related harms. Further investigation is required to develop standardised measurement tools and to understand confounding variables and co-morbidities. With a robust understanding of harms distribution in the population, Primary Care Workers will be better equipped to identify those who are at risk, or who are showing signs of Gambling Disorder, and to target prevention and intervention programmes appropriately.

**Supplementary Information:**

The online version contains supplementary material available at 10.1186/s12889-021-10337-3.

## Background

We know that excessive gambling can impact an individual’s finances, relationships, employment, and psychological wellbeing [1]. While some individuals may gamble without issue there are many who will experience negative consequences from their gambling behaviour. Policy makers within the UK, as well as the broader public health community, acknowledge the need for better understanding of gambling harms [2] in order to promote effective policies for harm reduction.

Global data suggests that in several jurisdictions with mature gambling markets participation rates have dropped significantly, whereas harm has plateaued [1],and within the Conceptual Framework of Harmful Gambling it is suggested that further harm reduction may need interventions to address a wider spectrum of risk, including socioeconomic factors. We predict that harmful consequences are not distributed evenly amongst the population, and in conducting this review we aim to identify which individuals are most at risk, and how harms are likely to present in the general population before clinical diagnosis.

The fifth edition of the Diagnostic and Statistical Manual of Mental Disorders (DSM-5) [3] categorises Gambling Disorder as a behavioural addiction. This is the first behavioural addiction to be included in the DSM and the condition is an increasing public health concern [4], as new accessible methods of play such as online and mobile gambling have led to an increase in new types of gambling behaviour. For diagnosis using the DSM-5, an individual must experience harmful consequences from their behaviour, and the exposure hypothesis suggests that increased availability of gambling tools increases the levels of harm and problematic gambling within a population [5], so understanding the potential harms resulting from gambling is more important than ever.

There have been a number of recent Systematic Reviews completed in the field of gambling, investigating a range of ideas. For example, the relationship between crime and gambling disorders [6, 7], quality of life measurement tools [8], comorbidity with other conditions [9] ,socioeconomic risk factors and vulnerable populations [10], impulsivity in gambling [11, 12], harms reported by significant others [13], or potential interventions and harm minimisation tools [14–17]. Despite this body of research, and many individual studies investigating specific gambling harms, a systematic review of how harms are distributed across society has not yet been done. Although many studies have investigated how harms can be minimised [18–20], a complete understanding of the disease and its impact on society is dependent on understanding how harms are distributed across the population.

If our intuitions from reviewing the literature are correct, then this poses a health inequality that needs addressing. Health inequalities are “unfair and avoidable differences in health across the population, and between different groups within society” [21]. For example, when one individual or population experiences more consequences, or more severe consequences, from a disease than another despite equivalent exposure. By understanding the distribution of harms within society we hope to identify at risk groups. This information could support harm reduction through a public health model of addiction, which suggests that interventions should target the host as well as the addictive ‘agent.’ In addition, interventions which target the environment, such as public health campaigns, could be targeted to reach the most vulnerable groups in order to reduce harm as effectively as possible [22].

Harms related to gambling behaviour have been found to affect all types of individuals, including low and moderate risk, or sub-clinical, gamblers [23–25]. However, evidence suggests that gambling harms are disproportionately experienced by economically and socially disadvantaged groups [10]. The National Strategy to Reduce Gambling Harm [26] states “An effective prevention plan must seek to identify the right mix of interventions to be applied at both the population and individual level,” and so a thorough understanding of how an individual experiences harm would be beneficial in understanding gambling as a whole, and developing effective interventions. Current estimates suggest that there are 2 million adults experiencing some level of harm from gambling in the UK alone [4] and an estimated 1.6 billion people gambling worldwide [27]. A thorough understanding of how harms are presented within these individuals, and within at-risk groups, may help in identifying those who are at risk and targeting interventions where they are most needed.

### Objective

To present a systematic review of the existing research into gambling harms, to determine whether there are differences in the presentation of these across demographic groups such as age, gender, culture, and socioeconomic status, or gambling behaviour categories such as risk severity and participation frequency.

Following the PICO model we determined that all potential participants would be considered, including all ages, genders, and cultural backgrounds from both clinical and general samples. Inclusion of an intervention is not relevant however we only considered studies which investigated harms to the individual. Comparison was made within sub groups, i.e. between genders or age groups. The intended outcome is to determine if a health inequality appears to exist in gambling and how different demographic groups experience harms to support primary caregivers in identifying patients who need support.

## Method

### Search strategy

In conducting the review we followed the Preferred Reporting Items for Systematic Reviews and Meta-Analysis Protocols (PRISMA-P) which can be seen in The PRISMA Checklist (Additional file 1). Studies that have explored specific harms and the prevalence of these within a population were identified using a search of records held by Web of Science. The database was searched on 18th August 2020 using the following criteria; TI = (gambl* AND (harm* OR “negative impact” OR “adverse impact” OR “detrimental impact” OR “negative? *ffect” OR “adverse? ffect” OR “detrimental? ffect” OR consequence)).* This yielded 189 results, which can be seen in detail in the Full Search Report (Additional file 2). An initial search within abstracts yielded 1997 results, however due to time constraints the search was restricted to titles only.

Search terms were chosen using ‘thesaurus.com’ [28], the Oxford English Dictionary Online [29], and the keywords of some relevant studies. The final criteria were developed with support from an Aston University subject librarian. The criteria were then adapted to search records held by Scopus; TITLE = (gambl* AND (harm* OR “negative impact” OR “adverse impact” OR “detrimental impact” OR “negative? ffect” OR “adverse? ffect” OR “detrimental? ffect” OR consequence)), and this search yielded 195 results, giving a combined 384 studies from both websites.

### Inclusion criteria

Studies were included that discussed gambling in terms of harm to the individual, and discussed or listed a minimum of one identified harm, rather than harm as a concept without specifics. Primary data sources were considered and grey literature was not included.

### Exclusion criteria

Studies to be excluded were those unrelated to gambling, and those that did not discuss harms. Further exclusion criteria included results not available in English, studies discussing the notion of harm without giving specific examples, those that only investigated harms to others, or from other related sources, and those that only discussed strategies for harm minimisation without measuring actual harms experienced.

### Screening

A free trial of Covidence was used to screen studies, along with EndNote software to organise the bibliography. Duplicate studies removed by Covidence totalled 147, and an additional 15 were removed during title and abstract screening. Title and abstract screening and full text screening were both conducted using Covidence by one researcher.

### Quality evaluation

Studies were assessed for quality by two researchers using the Standard Quality Assessment Criteria [30]. This measures the quality of both quantitative and qualitative research using a series of standardised questions, and we evaluated studies that followed a mixed methods approach in terms of the most prominent research style. The results of this assessment can be seen in the Table of Quality Checks (Additional file 3). Studies were coded in Excel using the guidelines set out by Kmet, Lee and Cook and coloured using a traffic light system for reviewing. Disagreements of more than one degree were discussed to reach a consensus, and scores were then combined to find an average.

### Data analysis plan

We extracted study design, country, participant sample, measures used, funding source, and relevant results on harm from the studies before identifying categories for analysis. Full extracted data can be seen in the Table of Extracted Data (Additional file 4). We divided the data into the identified categories for comparison, with several studies providing results for multiple groups.

Data was extracted from qualitative studies by highlighting key terms, and for relevant comparable data we have used the 73-Item checklist developed by Langham et al. [31] which identifies 8 domains of harm (Table 1). Delfabbro and King [32] argue that certain items attributed as harms are labelled incorrectly; they suggest that chasing losses, gambling to obtain more excitement, or betting above affordable means, are behaviours that lead to harm and not the harms themselves. Schellinck et al. [33]also argue that borrowing money is not a harm, but is in fact a predictor for the harms, debt and relationship conflict. Critical appraisal of the defined harms used in each study is therefore necessary.

**Table 1:** Langham et al. [31]Taxonomy of Harm Domains

Domain	Items Include but are not Limited to …
Financial Harms	Erosion of Savings Bankruptcy
Relationship Disruption, Conflict or Breakdown	Dishonest communication Social Isolation
Emotional or Psychological Distress	Distorted cognitions or erroneous beliefs Extreme distress
Decrements to Health	Reduced self-care Ongoing disability
Cultural Harm	Reduced engagement in cultural rituals Extreme cultural shame
Reduced Performance at Work or Study	Tiredness and Distraction Loss of Job
Criminal Activity	Vulnerability to illegal activities Arrest and/or conviction
Life-course and Intergenerational Harms	Loss of life-course event i.e. marriage Homelessness

A sample of harms identified by Langham, Thorne [31].

These comments were used when excluding studies from the research, for example MacLaren [34] discussed the CPGI and PGSI, but did not list actual specific harms, and Booth et al. [35] measured harm using only the PGSI rather than actual listed harms, so these studies were excluded. We also considered these criticisms of harm labelling when extracting data from studies, excluding behaviours such as chasing losses.

### Patient and public involvement

There was no involvement from the general population or any individual with a Substance Addiction or Behavioural Addiction Disorder in this systematic review.

## Results

### Search and selection results

The database searches returned 384 papers for review and 162 of these were excluded as duplicates. Analysis of titles and abstracts led to a further 9 exclusions for not discussing gambling and 20 exclusions for not discussing harms. The remaining 193 studies were reviewed in full, resulting in a further 6 exclusions for conflating gambling severity scores (i.e. PGSI) with harms, 9 exclusions for only discussing harms to others, 46 exclusions for only mentioning harm as a concept in general terms, and 57 exclusions for only discussing harm minimisation. There were 8 studies not in English, 2 were short letters, and 3 were abstracts for conference presentations. There were 2 studies which could not be accessed in full, and full-text requests to the authors were unsuccessful. Finally, 1 systematic review into harms [36] was removed because it described the process by which a systematic review would be conducted but did not report any results.

**Fig. 1:**
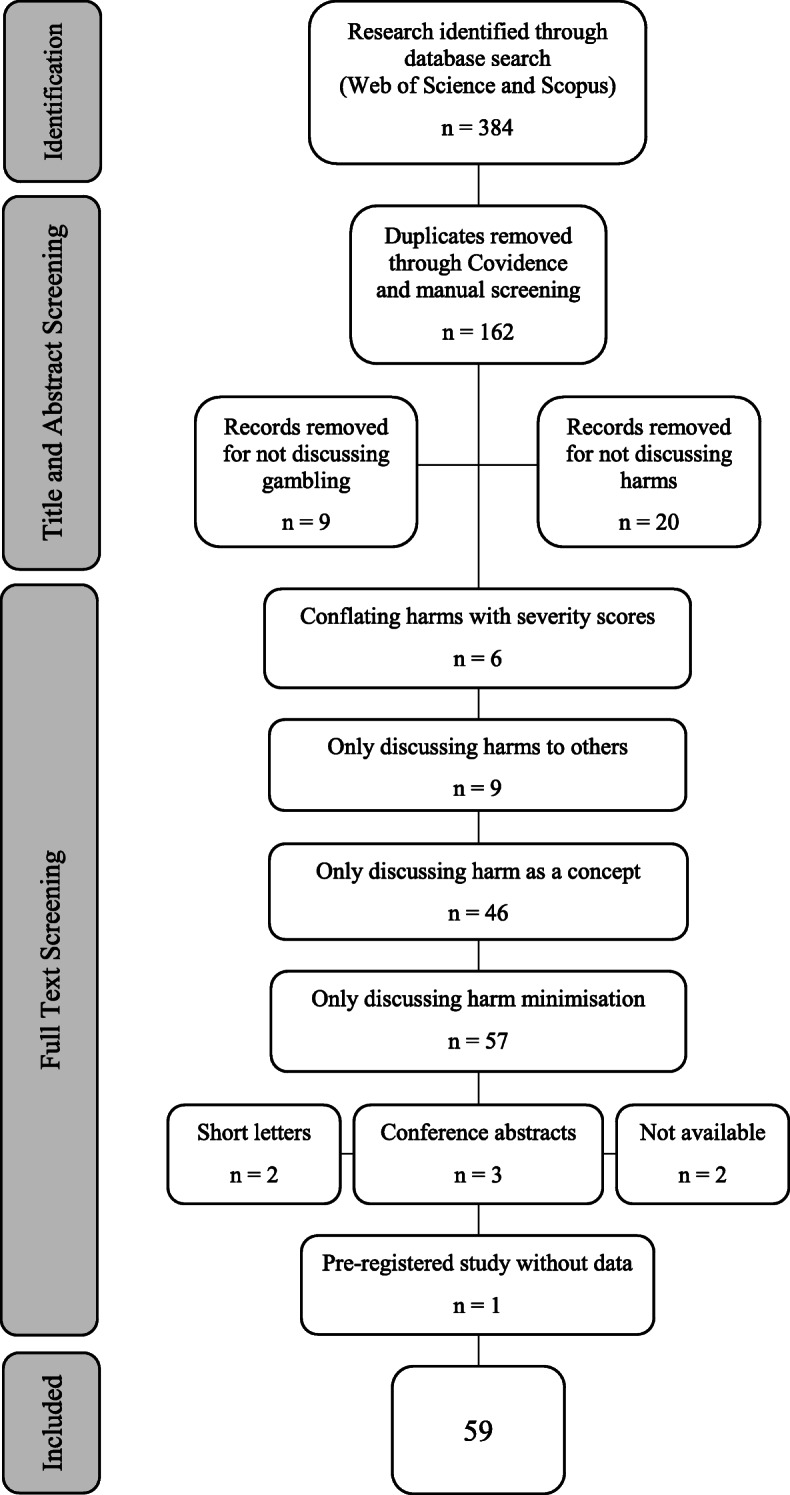
Preferred Reporting Items for Systematic Reviews and Meta-Analysis (PRISMA-P) Flowchart of Exclusions [37]

This left 59 studies for review of which 22 were qualitative, 36 were quantitative, and 2 were mixed methods design. Of the mixed method studies 1 was predominantly qualitative and 1 was predominantly quantitative (Fig. 1).

## Main results

### Description of included studies

Of the 59 studies included in this review, 5 were cohort studies, 2 were case-control studies, 16 were cross-sectional, 21 were qualitative, and 2 were mixed methods. Of the qualitative studies, 2 used multiple methods, 11 were interviews, 2 were focus groups, 4 were narrative reviews and 2 were systematic reviews. Secondary data analysis was conducted in 13 of the studies.

The most common funding sources for this selection of studies were the Ministry of Social Affairs and Health Helsinki (5) and the Victorian Responsible Gambling Foundation (6). In total, the government funded 12 studies,responsible gambling foundations funded 9, general research funds were used for 5, gambling focused research funds were used for 5, Colleges and Universities funded 4 studies, and there were 2 funding contest awards (International Contest ONCE; Irish Research Council Innovation Award). In addition, 2 studies were funded by alcohol foundations, 1 by a business school, 1 by a casino, 1 by a non-gambling charitable foundation, 1 by an information company, 1 by a gambling authority, and 1 by a psychiatric association. Of the remaining 14 studies, 8 received no funding and 6 did not declare their funding status (see Table 2).

**Table 2:**
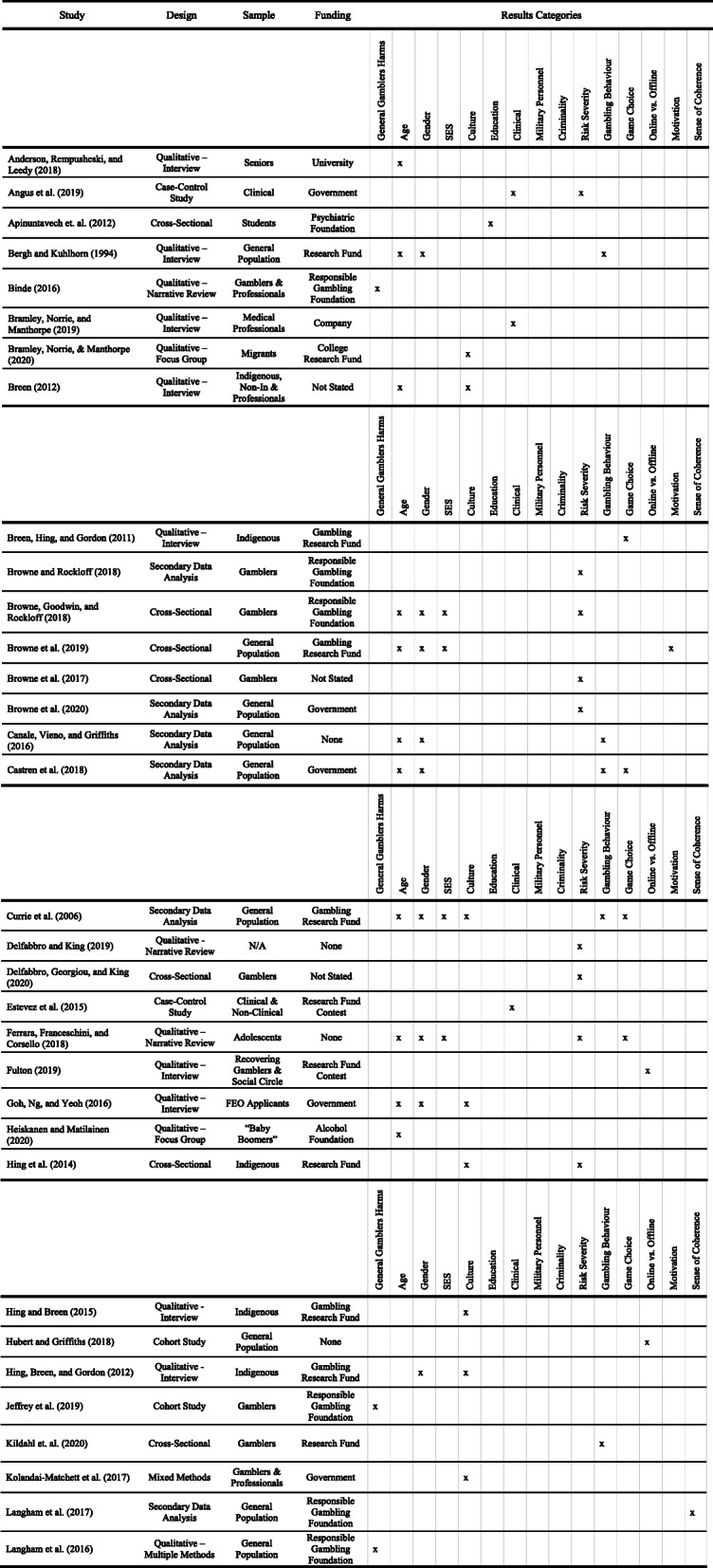
Summary of Data Extracted

### General gambling harms

Five studies include data on gambling harms generally, with some investigating specific harm locations, such as casinos or the workplace. Ricijas [38] reported that inappropriate social behaviour such as shouting at machines, aggression towards other patrons, appearing depressed, being withdrawn and excessive sweating were observed at all of the gambling venues included in their study. And Binde [39] found that participants identified gambling during work breaks and during work hours, poor work performance and lateness, depression and anxiety, tiredness and irritability, absences from work, tax authorities investigating staff wages, poor standards of self-care and belongings, and crimes such as embezzlement.

Jeffrey et al. [40] investigated how gamblers report and recognise harms in comparison to other individuals in their lives. They found that gamblers were more likely to report problems which impacted them individually such as lack of money, using work or study time to gamble, alcohol use, suicide attempts, hygiene issues, sleep problems, and feelings of shame or worthlessness. In comparison, spouses of gamblers reported shared harms such as missed bill payments and relationship tension or conflicts. The researchers suggested that this may mean gamblers are less aware of relationship dysfunctions. Another study [41] reported that harms in all domains accumulated more quickly in gamblers than in affected others.

Langham et al. [31] developed a taxonomy of gambling harms and found that many of the category domains interacted, or had individual specific outcomes. For example, cultural and relationship harms often appeared together due to the link between family and culture. They also reported that emotional harms were affected by all other domains, and criminality was often a second-order harm to address a primary harm such as financial issues. Financial harms reportedly led to a change in behaviour, however the crisis point was dependent on individual tolerance for deprivation. The level and type of relationship harm experienced appeared to be dependent on how the non-gambling person viewed gambling, and health harm was found to occur in recreational gamblers, but was not well documented. Finally, criminality was only found within those individuals who scored highly on risk severity measures.

### Age

Twenty-two studies include data on age, and several of these found that being younger was associated with a higher risk of experiencing gambling harms [42–47]. One study found that younger age groups (16–34) were at risk of dependence and social harms [42], and Ferrara et al. [45] found younger age groups showed higher rates of “problematic gambling” and a higher comorbidity with other addictions. In Breen [48] it was found that youths who were exposed to card gambling were more likely to gamble later in life to increase their income, and those who missed school had reduced lifelong aspirations and reduced opportunities. Salonen et al. [47] reported that financial harm, work and study harm, health harms, and emotional harm all tended to decline within the older age groups, and financial harm in particular was most common in the younger participants, and Splevins et al. [49] reported that students spent their pocket money or part-time job wages on gambling. Bergh and Kuhlhorn [50] reported that gamblers aged 20–34 spent more time gambling than those over 35, and Salonen et al. [51] also reported that females aged 18–24 increased their occasional gambling and consequently reported more harms.

In contrast, Raisamo et al. [52] reported that gambling involvement increased with age, and some studies found that younger gamblers were less at risk of financial harms [50, 52]. Larsen et al. [53] found that alcohol use increased with age in lifetime problem gamblers, as defined by the DSM-IV criteria for ‘pathological gambling’, in opposition to the trend seen in a general population. Whereas Pitt et al. [54] found that children aged 8–16 showed little or no current harms as they were gambling at home with their families, spending small amounts of pocket money, or betting with activities such as push-ups against family members. Despite this, children developed false beliefs around gambling, such as that skill can be used to win, or that it is necessary for everyone to gamble at least once. Similarly, Melendez-Torres et al. [55] found that harms increased with age; however, they only researched participants attending school who would be categorised in the younger age groups of other studies.

Livazovic and Bojcic [56] found that older participants scored higher on risk severity measures, however they did not report a difference in harms. Browne et al. [57] found that age had no impact on harm profiles, and Lloyd et al. [58] found no association between age and gambling-induced thoughts of self-harm. Browne et al. [59] found that although younger age appeared to correlate with harm this was not statistically significant, and Raisamo et al. [46] reported that guilt was not associated with age.

The remaining studies researched the distribution of harms within a single age group. Anderson et al. [60] reported that seniors who gambled experienced arguments, broken relationships, anxiety, debt, exhausted pensions or savings, and shame. Heiskanen and Matilainen [61] found that gamblers from the generation categorised as ‘Baby Boomers’ had difficulty walking past a machine without gambling, and spent excessive time and money both online and offline, and some participants reported that they felt unable to ‘meddle’ in another person’s gambling problems, suggesting there may be less peer support within this age group.

Further research is needed to understand the distribution of harms across age groups as it was found by Estevez et al. [62] that sensation seeking and impulsivity were high in young gamblers. Anxiety, depression and psychoticism were partially mediated by impulsivity, and somatisation, obsessive-compulsive behaviour, interpersonal sensitivity, paranoid ideation and hostility were perfectly mediated.

### Gender

Nineteen studies examined gender, and 5 of these found no difference between men and women [43, 50, 57, 59, 63]. Despite this, several studies showed that men have a higher prevalence of harms than women [42, 44–47, 52, 55, 56, 58], however Canale et al. [42] and Raisamo et al. [46] found that men gamble more frequently and spend more money when gambling. Raisamo et al. [46] in particular found that when controlling for frequency and spends, gender was no longer significantly related to harm. And in complete contrast Salonen et al. [51] reported that while gambling was more common in young males, women displayed an increase in specific harms between 2011 and 2015 where men did not.

Breen et al. [64] found that women from small villages and men from towns were both more likely to be heavy commercial gamblers, however harms were the same and so this was likely due to usage level rather than gender. Livazovic and Bojcic [56] found that males in Croatia scored significantly higher on psychological, social, and financial consequences than females. However, they also scored significantly higher on risk behaviour and were more likely to score as a problem gambler on the Canadian Adolescent Gambling Inventory. Splevins et al. [49] found that men started gambling earlier than women did and found it more exciting. This led to increased spending and therefore an increased risk of harms such as substance use and interpersonal conflicts.

Despite this some studies suggested differences in how gambling harms present between genders. In Singapore, Goh et al. [63] reported that “tentative evidence … points to the risk of child neglect when the problem gambler is the mother.” They also found that verbal abuse was most commonly males towards their mother, but found no difference in cases of physical abuse between genders. McCarthy et al. [65] found that women were more likely to report mental health comorbidity than males, however causality was not discussed, and Raisamo et al. [66] found that while the most common harm was guilt for both genders, the second was disrupted schoolwork for females and conflict with friends for males.

### Socioeconomic status

There were ten studies examining socioeconomic factors, and more than half of these studies concluded that less affluent socioeconomic groups are more at risk of experiencing harms than more affluent groups [44, 58, 67–70]. Angus et al. [67] found that clinical participants had significantly lower incomes than a community sample and a higher proportion of them reported harms. Currie et al. [44] concluded that participants who reported harms were more likely to be in a lower income bracket, and to have received no further education than high school. Similarly, Lloyd et al. [58] found that gambling related thoughts of self-harm, as well as acts of self-harm were more frequently found among the unemployed, although were not related to marriage status. Gambling related thoughts of self-harm were also found to be associated with parents gambling behaviour. And Skaal et al. [69] reported that urban residents were more likely to report psychological distress and those that scored as high risk of problem gambling on the PGSI were more likely to use alcohol.

Apinuntavech et al. [68] examined education level, and found that the average GPA of gambling participants was lower than non-gamblers. Gamblers subsequently had a higher risk of smoking, abusing alcohol and energy drinks, and reporting harms. The most common of which were psychological, in particular guilt, depression, anxiety, and considering suicide. These individuals also reported lying, perceived poor health, insomnia, debt, selling possessions, substance use, and school absence. Livazovic and Bojcic [56] found that lower achievers in school reported more psychological harms, however there was no difference between school types. However, Melendez-Torres et al. [55] reported that more harms and increased gambling behaviour were a result of feeling less school belonging.

Interestingly, Tu et al. [70] found that people in managerial or professional occupations were more likely to participate in gambling than people in routine (semi-skilled or unskilled) occupations. Melendez-Torres et al. [55] also found that participants from more affluent households were participating in more gambling than those from less affluent households. In light of this, they highlighted that more affluent individuals were reporting more harms, however Tu et al. [70] reported that although gambling rates in the most affluent groups dropped during times of recession, the rates within deprived communities did not. This suggests that less wealthy people may be more likely to gamble in times of economic stress. When controlling for confounding variables the most deprived groups were 4.5 times as likely to experience arguments or money issues.

The remaining studies found little to no effect from socioeconomic factors, with Browne et al. [57] reporting a difference of less than 5 points between individuals earning $15-30 k AUD and those earning $101-150 k AUD. Browne et al. [59] reported that part time work, unemployment, marriage status, lower education, and lower income all had large correlations, but these were statistically insignificant. And Livazovic and Bojcic [56] found that family life and parent’s education level had no significant effect on harms.

### Culture

Twelve studies include data on culture and five of these discuss Australia and New Zealand [48, 71–74]. The included studies largely focus on single groups or comparing indigenous people and migrants to a society, so there are significant gaps that future studies may address.

Hing et al. [73] interviewed Indigenous Australians and reported that female gamblers from small villages and male gamblers from towns both experienced similar harms. However, they were also heavy commercial gamblers, meaning they played at casinos and other commercial establishments. Hing et al. [72] interviewed counsellors who noted that cultural acceptance for gambling within Indigenous Australian communities was high, and so a strong support network was in place for individuals with a problem. Despite this, Indigenous participants’ highlighted isolation from the community as a key harm in a few studies [48, 71, 72], and missing key community events, neglecting children, lying, arguments, violence and breakups were found to lead to social isolation. Gamblers also admitted to hiding their losses due to shame, guilt and low self-esteem, which meant they were reluctant to seek help. In addition they reported financial problems, and outside criticism or lack of support [71, 72], as well as debt, lack of resources [48, 72], distress, cut off utilities, crime, loss of employment, and homelessness [72]. Breen [48] also noted that many people would gamble within a group, increasing their behaviour, but also feelings of shame from losses and potential gossip. Similarly, Hing et al. [71] reported that participants were betting above their means, felt the need to spend more, borrowed or sold, and had health problems.

Goh et al. [63] found that families in Singapore were at risk of acute financial harms when the problem gambler was a parent, with households suffering double financial harms through loss of income and debt. When the gambler was a mother without income, they found that the father would leave employment to care for the children, resulting in an income reduction for the entire household. They also found that many people in Singapore viewed gamblers as self-centred, and siblings would often give up on them.

Kolandai-Matchett et al. [74] found that Pacific New Zealand people experienced similar gambling harms to other populations. However, the context of collectivist cultural values meant that additional harm dimensions were present, such as a loss of belonging or isolation, shame, loss of the community’s respect, disruption of trusting relationships, transference of communal responsibilities, and an overall loss of social cohesion. In a quotation from one of the interviewed participants, the researchers noted that the wider collective might exclude non-present or non-contributing members of the society. Similarly, Bramley et al. [75] found that migrants in the UK reported similar harms to the general population, including selling possessions, relationship breakdown, mental health problems, drug use and sale, homelessness, domestic violence, sex work and suicide. Despite this, participants felt that harms were exacerbated by a lack of ‘safety net’ and difficulty accessing informal support. Sub-Saharan African men in particular felt that when they lost money they lost community status.

McCarthy et al. [65] conducted a worldwide study which suggested that women from ethnic minorities, indigenous communities and specifically Maori and Pacific women in New Zealand were more vulnerable to gambling harms than European women were. Melendez-Torres et al. [55] also found that participants from white ethnicities were less likely to feel guilt from gambling, and a non-white British background was associated with more harms. Ferrara et al. [45] found that non-white males were most at risk of developing a gambling problem and addiction comorbidity, and Wardle et al. [76] found that although migrants were less likely to gamble they were more likely to experience harms than individuals born in the country. They found minimal evidence on specific harms experienced, but did report that Spanish migrants tended to spend over 300 euros daily and claim losses as wins, and Australian migrants experienced financial harm, shame, relationship issues, suicide, mental health issues, isolation and prostitution. Similarly, Currie et al. [44] found that in Canada, non-white men were more likely to have reported two or more harms in the last year.

### Clinical

Five studies reported on a clinical sample and all of these found more harms within a clinical population compared to the general community. Angus et al. [67] reported that 100% of their clinical sample reported psychological harms, compared to only 14.85% of the non-clinical participants. And while they found a greater severity of harm in all domains for the clinical sample, they specifically found a 97.98% response on financial harms compared to 23.33% in the non-clinical sample. Similarly Bramley et al. [77] reported that a clinical sample with habitual gambling showed high levels of anxiety, financial difficulties and depression.

Salonen et al. [47] reported that while 11% of a general sample experienced at least one harm of any domain, they found that 88% of the clinical sample reported emotional harms, 87% financial or health, and 81% experienced relationship harms. The specific harms reported were similar for all domains apart from emotional harm, where the clinical sample reported more anger, as well as being more likely to promise to pay debts without intending to, more likely to steal, and more likely to feel like an outcast.

Shannon et al. [78] found that the highest rated harms within their clinical sample were reduced savings, going without, worry, frustration, and debt. The lower rated consequences included drug use, suicide, bankruptcy, self-injury, and educational problems. In contrast the general population rated debt, relationship issues, feeling constrained, going out less, poor self-control and lowered pride highest. Despite these different results the averaged distribution of harm was consistent across both samples, excluding reduced savings and decreased happiness.

Finally, Estevez et al. [62] reported that young adults within their clinical sample had more dysfunctional symptomology. Specifically anxiety, depression, hostility, out of character behaviour, and somatisation. They also found high comorbidity for alcohol, drug, gaming, shopping and sex ‘addiction’. Despite this they found no significant differences for eating behaviour or internet use, and when repeating the analysis discovered that impulsivity partially mediated anxiety, depression and psychoticism. While perfectly mediating somatisation, OCD symptoms, interpersonal sensitivity, paranoid ideation and hostility.

### Military personnel

Only one study reported on a military population [79], however this was a systematic review of existing literature. One examined study found that individuals would be quickly reprimanded for gambling, but meaningful assistance was slow to come, whereas another found that 21/25 active personnel who received treatment were retained in the military, compared to the 4 who lost their jobs. Several of the investigated studies highlighted comorbid mental health problems with gambling in the military, including suicide. It was also found that 9/35 gamblers receiving treatment had depressive disorder, 20% endorsed suicidal ideation and 3 participants had made actual attempts on their life.

### Criminality

May-Chahal et al. [80] investigated harms within the British prison population and found that although the prevalence of gambling was higher in prisons, the prevalence of gambling behaviour prior to incarceration was significantly lower. They found that high rate offenders in their mid-20s were 5.3 times more likely to be frequent loss chasers than other categories, and occasional gamblers were less likely to use alcohol or drugs in prison, with nearly 2/3 of the problem-gambling group abstaining completely from substance use. The researchers suggest that this may be because the individuals’ ‘addiction needs’ are being met by their gambling behaviour.

### Risk severity

Nineteen studies include data on risk severity, which is the measure of behaviour that puts someone at risk of developing a problem with gambling or experiencing harms from gambling. Angus et al. [67] found that the number of harms experienced increased with PGSI classification, and significantly less low-moderate risk gamblers reported harms compared with problem gamblers. Problem gamblers were also more likely to come from the clinical sample, who had significantly greater severity of harms in all domains. Similarly, Delfabbro et al. [81] reported that ‘problem gamblers’ experienced more harm in general than lower risk groups. In fact, the number of gambling harms within the lower risk categories was close to zero in all but the financial and psychological domains. Ricijas et al. [38] also found that social gamblers had no consequences, moderate risk occasional gamblers experienced low-moderate harms, and high risk frequent gamblers suffered serious consequences. Specifically in terms of delinquency and cognitive distortions.

In contrast, Browne et al. [57] reported that the prevalence of harm within a non-problem gambling group was twice that of the problem category, and Raisamo et al. [46] found that most of the harms reported originated from low-moderate risk participants. However, when scaling for severity of harms, Delfabbro and King [32] reported that low and moderate risk participants experienced only a low-medium severity of harm. Interestingly, more severe financial harms, such as selling belongings, were found in the lowest risk group even when scaling. However, there was a significant number of participants from less affluent socioeconomic backgrounds, which the researchers suggest may impact these results.

In considering scaling, Browne et al. [82] reported that all individuals in the high risk category reported at least one harm, and while mild harms were broadly distributed across all risk groups, severe harms were repeatedly more prevalent in the highest risk group. Hing et al. [71] found that 93.8% of high risk gamblers spent more than they could afford to lose, and 92.9% felt the need to bet more each time for the same thrill. Family arguments were experienced by 18% of moderate risk gamblers, compared to only 0.9% of low risk participants, and 94.9% of high risk participants had a gambling related health issue. Browne et al. [57] found that only 10% of financial harms across the study population were in the problem gambling or pathological gambling groups and that more than 50% of cases where someone sold their belongings to fund their gambling were in recreational or low risk gamblers. In contrast to this, they found that more than 50% of social deviance harms are found within problem gamblers, and the remaining categories of harm were evenly distributed across the severity groups.

Langham et al. [31] reported that criminality was only found within high risk participants, and Skaal et al. [69] found psychological distress was only associated with problem gambling. Similarly, Splevins et al. [49] reported that high scoring participants were more likely to miss school, sell their personal property, commit illegal acts, and use cigarettes or drugs. Larsen et al. [53] found that harmful alcohol and marijuana use were common among high risk scorers, and Yani-de-Soriano et al. [83] reported the highest degree of harm across all domains was found in high risk participants. Specifically reporting that as risk scores increased, so did physical, mental health, social, and academic harms.

Browne et al. [84] conducted their study using disability weights, a health-related measure of quality of life which uses a ratio scale between 0 and 1, representing ideal health and death. They found that problem gamblers show similar disability weights to those of Bipolar Disorder or alcohol dependence, whereas the low risk group show disability weights equal to moderate anxiety. In addition, they reported that the less severe harms were experienced by a large proportion of the population, compared to the intense harms, such as suicide attempts, which were mostly confined to the highest risk participants.

Li et al. [41] found that selling personal items, absence from work or study, reduced performance, poor sleep and extreme distress had the highest correlation with PGSI categories. They also found that reduced spending on essentials, absence from work or study, feelings of worthlessness, relationship conflict, and feeling like an outcast were the most effective discriminators between the low and high-risk groups. Similarly, Ferrara et al. [45] found that participants rated as high risk were more likely to use alcohol or substances, have depression, dysthymia, anxiety, phobia, and anger, resentment, headaches, gastrointestinal problems, eating disorders, and criminality, as well as family conflict, less independence, less engagement in intellectual or cultural activities, and reduced expression of emotion.

In contrast, Livazovic and Bojcic [56] reported only a weak correlation between success in school and risk score, and May-Chahal et al. [80] found that nearly two thirds of high risk participants in the prison system were actually abstaining from drugs and alcohol.

### Gambling behaviour

Thirteen studies include data on gambling behaviours and many of these studies agreed that a higher frequency of play, and higher amount of spending per session, leads to more harms [42–44, 46, 50, 52, 85–87]. In particular, Castren et al. [43] found that spending at least 1% of your monthly income increased harms, and daily gambling doubled them. Kildahl et al. [85] also reported that overconsumption of money and time, social consequences, and emotional consequences all increased linearly with gambling frequency.

Samuelsson et al. [87] found that low frequency stable gamblers only reported mild harms such as shame or guilt, whereas high frequency gamblers with decreasing use experienced substantial financial losses, frustration, alcohol use, and isolation. They also noted that periodic gamblers experienced financial, psychological, and relationship harms, including insomnia, isolation, and low self-esteem. The most severe harms, such as irrational thought and increasing spends, were found in the high frequency gamblers with increasing use. However they did find that financial harms and psychological distress could lead to a period of reduced play depending upon an individual’s support network.

Similarly to participation frequency Lloyd et al. [58] reported that number of years gambling was associated with thoughts of self-harm, and Rintoul et al. [86] found that gambling fast and intensely lead to more harm. Specifically highlighting multiple machine use, skipping meals, withdrawing money multiple times and betting over $3 per spin. Interestingly, Canale et al. [42] reported that most of the identified harms in their study were reported by non-high time and spend regular gamblers. Despite this, harm odds increased with greater frequency of play individually, suggesting a higher individual risk in high volume play, but a larger proportion of at least one harm among low volume players.

Five studies looked at motivations for gambling, and although Browne et al. [59] found no link between motivation of play and harms, Lee et al. [88] found that excitement, escape and challenge motives were linked with positive outcomes, but financial motivation led to harms. Lloyd et al. [58] also found that self-harm thoughts were associated with money as a motivator but was negatively associated with enjoyment motivations, and Kildahl et al. [85] reported individuals who were influenced by reward frequency were more likely to swap card decks rather than persevere with the same cards. This led to overconsumption of time and money, and negative social and emotional consequences.

Similarly, Mageau et al. [89] found that harmonious passion was related to positive emotions and thoughts, whereas obsessive passion lead to harms. Harmonious passion is when an individual chooses to gamble, whereas obsessive passion is when someone feels compelled to gamble. Mageau et al. [89] reported that in comparison to harmonious passion, obsession was strongly related to feelings of guilt, anxiety, and negative emotions, and negatively correlated with feeling in control and having fun.

### Game choice

Game choice also affected harms, and nine studies reported on this relationship. Breen et al. [64] found that card games led to financial losses and lost welfare benefits, whereas commercial gambling (i.e. Casinos, EGMs) led to financial hardship, family and relationship issues, mental health issues, crime, eviction, homelessness, domestic violence, neglect, relationship breakdown, depression, suicidality, theft, and sold belongings. Hing et al. [73] reported that heavy card players spent their pensions, borrowed money, and played all day and night. Similarly, heavy commercial players gambled alone, spending their whole pay and playing all day and night. They experienced debt, relationship issues, lost home, overcrowded housing, missed bills, lack of resources, abuse, neglect, self-esteem issues, depression, suicidality, theft, selling belongings and crimes against their workplace. Ferrara et al. [45] also found that sports betting was associated with high rates of addiction comorbidity, Mihaylova et al. [90] found that online poker players had higher annual debts, and Ricijas et al. [38] reported that sports bettors, VLT users, and virtual bettors showed severe psychosocial consequences.

When considering casino gambling Mageau et al. [89] also found more negative consequences than in lottery players. However, they also reported more positive outcomes overall in casino gamblers. Similarly McCarthy et al. [65] found that older women believed electronic gaming machines were less harmful than other games as they were able to socialise while gambling.

Castrén et al. [91] found that six out of twelve game type predictors were associated with more harmful consequences, including scratch games, betting, slot machines, non-poker online games, online poker, and non-monopoly games. They found that lottery play caused the lowest number of harms, and this finding is consistent with findings reported by Currie et al. [44] who found that frequency of play on lottery games did not increase the harms experienced, whereas electronic gambling machines, ticket gambling, bingo and casino games did.

### Online vs. offline gambling

As well as specific game type six studies look at the broader categories of online or offline gambling. Castrén et al. [91] found only a weak link between online gambling and an increase in harms, however Mihaylova et al. [90] found that online poker players had a greater risk of alcohol dependency, illicit drug use, family issues, studying issues and financial issues in comparison to offline poker players.

Yani-de-Soriano et al. [83] found that online gambling was associated with binge drinking but not smoking, and around 60% of online gamblers scored as high risk for gambling problems. These increased risk severity scores in turn led to increased physical, mental health, social, and academic harms. Hubert and Griffiths [92] also found a link between online gambling and alcohol dependence, and they discovered that online gamblers were less likely to have jobs, children and a stable relationship, leading to unemployment and less money later in life. They further found that online gamblers were less able to control impulsivity and frustration, but despite this, they had fewer suicidal thoughts than offline gamblers, although actual suicide attempts were comparable in both groups.

Feelings of anxiety and guilt appeared to be higher in online gamblers relative to offline gamblers [88]. However, Fulton [93] observed that secretive gambling increased financial harms due to the likelihood of concealed debt; and by living a double life secretive gamblers experienced increased stress, relationship conflicts, and emotional deterioration.

### Sense of coherence

Langham et al. [94] found that an individuals’ sense of coherence correlated strongly with gambling harms in all domains. Sense of coherence is the extent to which someone feels confident in the predictability of his or her environment, and that things will generally turn out as expected. They reported specifically that a stronger sense of coherence meant fewer harms, and that a weaker sense specifically led to reduced spending on essential items, increased negative health behaviour such as lost sleep, reduced physical activity, and poor nutrition, as well as stress related illness and depression. Weaker sense of coherence also resulted in feelings of failure, worthlessness, hopelessness, shame, anger and feeling the need to run away. Despite this, a weaker sense of coherence was not related to increased risk of suicide.

### Quality checks

In applying the Standard Quality Assessment Criteria [30] we found that several studies were not robust in their quality control. In particular, studies scoring below 0.5 on the assessment may not be an accurate representation of gambling harms, whereas studies that scored above 0.9 may present the most reliable data on harm distribution (Table 3).

**Table 3:** Highest and Lowest Quality Assessment Scores [30]

Study	Highest Scores	Study	Lowest Scores
Angus et al. (2019)	1.00	Browne and Rockloff (2018)	0.86
Browne et al. (2019)	1.00	Browne et al. (2017)	0.86
Browne, Goodwin, and Rockloff (2018)	1.00	Hing et al. (2014)	0.86
Delfabbro, Georgiou, and King (2020)	1.00	Li et al. (2017)	0.86
Langham et al. (2017)	1.00	Tu, Gray, and Walton (2014)	0.86
Larsen, Curtis, and Bjerregaard (2013)	1.00	Splevins et al. (2010)	0.82
Mihaylova, Kairouz, and Nadeau (2013)	1.00	Salonen et al. (2018)	0.77
Raisamo et al. (2015)	1.00	Yani-de-Soriano, Javed, and Yousafzai (2012)	0.77
Salonen, Alho, and Castren (2017)	1.00	Goh, Ng, and Yeoh (2016)	0.71
Canale, Vieno, and Griffiths (2016)	0.95	Hing, Breen, and Gordon (2012)	0.71
Estevez et al. (2015)	0.95	Anderson, Rempusheski, and Leedy (2019)	0.68
Hubert and Griffiths (2018)	0.95	Apinuntavech et al. (2012)	0.68
Jeffrey et al. (2019)	0.95	Langham et al. (2016)	0.68
Kildahl et al. (2020)	0.95	Pitt et al. (2017)	0.68
Lee, Chung, and Bernhard (2014)	0.95	Samuelsson, Sundqvist, and Binde (2018)	0.68
Lloyd et al. (2016)	0.95	Wardle et al. (2019)	0.68
Mageau et al. (2005)	0.95	Breen (2012)	0.64
Raisamo et al. (2013)	0.95	Breen, Hing, and Gordon (2012)	0.64
Ricijas, Hundric, and Huic (2016)	0.95	Fulton (2019)	0.64
Shannon, Anjoul, and Blaszczynski (2017)	0.95	Heiskanen and Matilainen (2020)	0.64
Skaal et al. (2016)	0.95	Rintoul, Deblaquiere, and Thomas (2017)	0.64
Browne et al. (2020)	0.91	Hing and Breen (2015)	0.61
Castren et al. (2018)	0.91	Kolandai-Matchett et al. (2017)	0.61
Currie et al. (2006)	0.91	Bramley, Norrie, and Manthorpe (2020)	0.57
Livazovic and Bojcic (2019)	0.91	Paterson, Whitty, and Leslie (2020)	0.57
May-Chahal et al. (2017)	0.91	Bramley, Norrie, and Manthorpe (2019)	0.54
Melendez-Torres et al. (2019)	0.91	McCarthy et al. (2019)	0.46
Raisamo et al. (2019)	0.91	Binde (2016)	0.43
		Bergh and Kuhlhorn (1994)	0.39
		Delfabbro and King (2019)	0.36
		Ferrara, Franceschini and Corsello (2018)	0.29

## Discussion

The results presented here suggest that there may be a health inequality in gambling harms, as several studies have found differences in the number and types of harms reported in different social groups. Although further analysis and investigation is necessary for a complete understanding of the distribution of gambling harms in society, the results suggest that there are differences that are dependent upon several factors. Studies such as Wardle et al. [76], Castrén et al. [91] and Tu et al. [70] pose a particular concern as there are suggestions that certain groups experience more harms even when gambling less, presenting a health inequality that needs to be understood and addressed. In particular, several studies report differences between age groups, socioeconomic status, and gambling behaviour or play styles.

In considering the differences found between demographic groups we can make some assumptions for why harms may be more acute in certain groups. For example, financial hardships can be traced back to losses while gambling excessively, however where an affluent individual may be able to lose 50% of their monthly wage and still survive, a less affluent person might no longer be able to pay their bills, or purchase necessary items such as food. Despite this, some harms are not as clearly tracked, and we need to examine how successfully each study can attribute the harms measured to actual gambling behaviour.

Most studies included in this review are cross-sectional, and therefore it is difficult to confidently derive causal relationships. While some studies will ask participants to consider gambling harms specifically, human error is likely to cause participants to mistake the source of certain harms.

It is also important to consider the quality of these studies to determine the most reliable and valid results. For example Hing et al. [73], who reported a difference in harm between genders, score only 0.64 in our quality assessment. This suggests that their results may not be as robust as Raisamo et al. [46] who scored 1.00 and reported no differences in harm between the genders. When examining the results of the highest scoring studies in each category a few patterns seem to emerge.

In particular, individuals who gambled more frequently and spent more money were found to have the highest number of harms [42, 52], and Currie et al. [44] found that harms increased significantly when an individual gambled more often than once per week. Raisamo et al. [46] showed that when controlling for frequency of play gender differences were no longer significant, and so the most interesting results may be those where more harms occur despite reduced play time. For example, Raisamo et al. [52], who scored 0.95 on the quality assessment, found that while older participants gambled more, harms reported differed very little, suggesting younger participants were experiencing the same level of harm despite lesser involvement. Similarly, Salonen et al. [51] found that harm reports increased for females, but not males, despite both genders gambling more frequently.

Despite this many of the high rated studies found no significant differences between groups [43, 57–59], or reported differences without considering participation frequency. For example, two studies on age found more harm among older participants [53, 55], although Melendez-Torres et al. [55] only conducted their study within school-age participants. Whereas seven studies all indicated younger age as a predictor of increased harms [42–44, 46, 51, 52, 56]. Similarly three studies which found more harms in males than females did not examine frequency of play [44, 55, 58]. However, three found that male participants displayed higher frequency of play and higher spends while reporting more harms [42, 46, 56], and one study did report more harms in non-frequent male gamblers [52].

Unfortunately no evidence was found on the differences in non-binary genders, suggesting there may be a gap in the research. This missing information could be significant in understanding the impact of brain structure on gambling, as past research has suggested similarities between cisgender brain structures and the brains of transgender individuals in terms of their identified gender [95–97].

Despite not including data on participation frequency four highly rated studies suggest that living in an urban area, having a low income, less feelings of school connectedness, or being unemployed predicted more harms from gambling [44, 56, 58, 69]. And while three studies reported little or no significant differences [56, 57, 59], Melendez-Torres et al. [55] reported that more affluent participants had more harms with a higher frequency of play.

The two high quality studies reporting on culture both found that non-white participants experienced more harms [44, 55], and two studies with a clinical sample found significantly more harm experienced by the clinical participants [62, 67]. However these studies also lack detail on the different participation frequency and spending habits of participants.

The findings on risk severity showed that the majority of harm impact was present in the lower risk rated groups [24, 42, 46, 67], suggesting more harms within individuals who gamble less often. Where the majority of cases of a disease come from a population at low or moderate risk of that disease this is known as the prevention paradox. In the case of gambling this means, the majority of harms are found within the low to moderate risk gamblers, and the minority is found within high risk or problem gamblers. However, these harm reports were collective rather than individual and so increased harm numbers are due to the larger population in these categories. Interestingly, the high quality studies also found that the highest severity of harm, such as alcohol use [53] or severe psychological distress [69], was most often present in the problem-gambling group. Angus et al. [67] in particular reported significantly greater severity of harms in all domains for the clinical sample, even when controlling for those community participants who reported zero harms.

This suggests that rather than looking at reported numbers of harm, it may be more important to consider an overall harm score which considers the severity of reported harms as well as the total number of separate experiences. For example, one individual may report shame, psychological distress, and homelessness, and rather than considering this individual as having three harms it may be more beneficial to categorise each harm and produce a harm score.

Although several studies included in this review discuss the different severity of harms a significant amount of work would be necessary to accurately categorise harms, as individual interpretation and circumstances could influence how severe a person considers one consequence to be in comparison to another. A married person with children may rate relationship breakdown as more severe than somebody who has been in a relationship for less than a month. This difference can be seen in Shannon et al. [78] where clinical participants named reduced savings, doing without and worry as the highest rated harms, whereas the community sample highlighted debt, partner issues, and not going out as often.

The vague definitions of harm used in several studies can also impact results, as where one researcher may count chasing losses as a harm another may not. The research team who consider additional harms may therefore find harms within a particular group, where a team who are stricter in labelling would not consider that demographic to be experiencing harms. This could explain some of the variation in results between studies. In future research it would be beneficial to have a taxonomy of harms which is robust, does not conflate risk with actual harm, and can be replicated across multiple studies for comparison.

It was stated by Susana Jiménez Murcia that, “we need to use different treatments for each sub-group of pathological gamblers.” Murcia is the co-author of a study that found that there are four distinct types of gambler [98]. The team concluded that out of the four sub-types only one category of gambler suggested significant pathology, though all were compulsive with differing severity levels, comorbidity and personality profiles. Future research could investigate not only the distribution of harms across society when controlling for participation frequency, but also further understand these sub-types of gambler, attribute them to specific groups or personality profiles, and compare and validate the results against this previous work. The participant base should also be broader, since Álvarez-Moya et al. [98] only investigated self-reporting slot machine gamblers, meaning their results may not be complete, or may not be generalizable.

Further research is needed to fully understand gambling harms and to confirm which individuals and groups are most at risk. In particular, advancing our understanding will depend on researchers considering frequency of play and spending habits, harms without including risk factors, the possibility of harms coming from other sources and ways to manage this, and considering the complex task of categorising harm by severity rather than counting each individual harm as equal.

## Conclusions

To conclude, our review strongly suggests that the distribution of harms in the population is affected by a number of factors, and presents some key signs to identify individuals who may be at risk. The type and number of harms experienced by individuals appears to be dependent on specific social, demographic and environmental conditions such as age, cultural background and socioeconomic status. There is evidence to suggest a health inequality is present, where some individuals will suffer more harms than others, despite equivalent exposure to gambling. With this in mind, Primary Care Workers will be better equipped to identify those who are most at risk, or who are showing signs of Gambling Disorder, and to target prevention and intervention programmes appropriately.

## Limitations

Despite these results it is important to consider the limitations of the study when reviewing the data. Due to time constraints search criteria were limited to titles only, and restricted to just two databases. This could lead to some important research being missed, and so with more time, and a larger team of reviewers, a search of titles and abstracts with additional databases would be more appropriate.

Only one reviewer screened search results. A larger team of reviewers would help remove the risk of bias and human error in screening. This would also allow for more than two researchers to complete quality checks on included studies.

Many of the studies included harmful consequences that are not universally accepted. For example chasing losses and betting above affordable means may be behaviours that lead to harms, and borrowing money could be considered a predictor of harms such as debt or relationship conflict.

## Supplementary Information

**Additional file 1.** The PRISMA Checklist. Table showing the PRISMA checklist completed in relation to this review as a PDF

**Additional file 2.** Full Search Report. Full Electronic Report of Web of Science Search conducted 18^th^ August 2020 as a PDF

**Additional file 3.** Table of Quality Checks. Table of the quality assessment completed by researchers as a PDF

**Additional file 4.** Table of Extracted Data. Full table of data extracted from included studies as a PDF

## Data Availability

Data sharing is not applicable to this article as no datasets were generated or analysed during the current study.

## Abbreviations

AISS: Arnett Inventory of Sensation Seeking
AUD: Australian Dollars
AUDIT: Alcohol Use Disorder Identification Task
BBGS: Brief Biosocial Gambling Screen
CAD: Clinical Assessment of Depression
CAGI: Canadian Adolescent Gambling Inventory
CAGE: Cut-Annoyed-Guilty-Eye Alcohol Questionnaire
CPGI: Canadian Problem Gambling Index
CSPG: Consumption Screen for Problem Gambling
CQLS: Comprehensive Quality of Life Scale
DSM: Diagnostic and Statistical Manual
EGM: Electronic Gambling Machine
GES: Games and Emotions Scale
GFM: Gambling Fallacies Measure
GOES: Gambling Outcomes Expectancies Scale
GPA: Grade Point Average
GPSS: Gambling Problem Severity Scale
IOWA: Iowa Gambling Task
KPDS: Kessler Psychological Distress Scale
NODS: National Opinion Research Centre DSM-IV Screen for Gambling Problems
PGSI: Problem Gambling Severity Index
PPGM: Problem and Pathological Gambling Measure
PRISMA: Preferred Reporting Items for Systematic Reviews and Meta-Analyses
PRIME-MD: Primary Care Evaluation of Mental Disorders
SGP: Safe Gambling Practices
SGHS: Short Gambling Harms Screen
SOC: Spheres of Control
SOGS: South Oaks Gambling Screen
VGS: Victorian Gambling Screen
VLT: Video Lottery Terminal
