# Supplementary material for: Is there a health inequality in gambling related harms? A systematic review
Source: BMC Public Health. 2021 Feb 6;21:305. doi: 10.1186/s12889-021-10337-3 (PMC7866763; doi:10.1186/s12889-021-10337-3)
Supplement: Supplementary file 2 — Additional file 2. Full Search Report. Full Electronic Report of Web of Science Search conducted 18th August 2020 as a PDF [file 12889_2021_10337_MOESM2_ESM.pdf]

Web of Science

Record 1 of 189

Title: Gambling-related harms attributable to lotteries products

Author(s): Booth, L (Booth, Leon); Thomas, S (Thomas, Samantha); Moodie, R (Moodie, Rob); Peeters, A (Peeters, Anna); White, V (White, Victoria); Pierce, H (Pierce, Hannah); Anderson, AS (Anderson, Annie S.); Pettigrew, S (Pettigrew, Simone)

Source: ADDICTIVE BEHAVIORS **Volume:** 109 **Article Number:** 106472 **DOI:** 10.1016/j.addbeh.2020.106472 **Published:** OCT 2020

Accession Number: WOS:000539099900004

PubMed ID: 32485546

Author Identifiers:

Author	Web of Science ResearcherID	ORCID Number
anderson, annie		0000-0002-0047-4500

ISSN: 0306-4603

eISSN: 1873-6327

Record 2 of 189

Title: Association between gambling and self-harm: a scoping review

Author(s): Gray, HM (Gray, Heather M.); Edson, TC (Edson, Timothy C.); Nelson, SE (Nelson, Sarah E.); Grossman, AB (Grossman, Alessandra B.); LaPlante, DA (LaPlante, Debi A.)

Source: ADDICTION RESEARCH & THEORY **DOI:** 10.1080/16066359.2020.1784881 **Early Access Date:** JUL 2020

Accession Number: WOS:000550933400001

ISSN: 1606-6359

eISSN: 1476-7392

Record 3 of 189

Title: Online Gambling-Related Harm: Findings from the Study on the Prevalence, Behavior and Characteristics of Gamblers in Spain

Author(s): Diaz, A (Diaz, Alejandro); Perez, L (Perez, Levi)

Source: JOURNAL OF GAMBLING STUDIES **DOI:** 10.1007/s10899-020-09966-x **Early Access Date:** JUL 2020

Accession Number: WOS:000550120800001

PubMed ID: 32686040

Author Identifiers:

Author	Web of Science ResearcherID	ORCID Number
Perez, Levi	L-6919-2015	0000-0001-6632-6626
Diaz, Alejandro		0000-0002-6545-9432

ISSN: 1050-5350

eISSN: 1573-3602

Record 4 of 189

Title: Open letter from UK based academic scientists to the secretaries of state for digital, culture, media and sport and for health and social care regarding the need for independent funding for the prevention and treatment of gambling harms

Author(s): Wardle, H (Wardle, Heather); Banks, J (Banks, James); Bebbington, P (Bebbington, Paul); Blank, L (Blank, Lindsey); Jones, HB (Jones, Henrietta Bowden); Bramley, S (Bramley, Stephanie); Bunn, C (Bunn, Christopher); Casey, E (Casey, Emma); Cassidy, R (Cassidy, Rebecca); Chamberlain, SR (Chamberlain, Samuel R.); Close, J (Close, James); Critchlow, N (Critchlow, Nathan); Dobbie, F (Dobbie, Fiona); Downs, C (Downs, Carolyn); Dymond, S (Dymond, Simon); Fino, E (Fino, Emanuele); Goyder, E (Goyder, Elizabeth); Gray, C (Gray, Cindy); Griffiths, M (Griffiths, Mark); Grindrod, P (Grindrod, Peter); Hogan, L (Hogan, Lee); Hoon, A (Hoon, Alice); Hunt, K (Hunt, Kate); James, R (James, Richard); John, B (John, Bev); Manthorpe, J (Manthorpe, Jill); McCambridge, J (McCambridge, Jim); McDaid, D (McDaid, David); McKee, M (McKee, Martin); McManus, S (McManus, Sally); Moss, A (Moss, Antony); Norrie, C (Norrie, Caroline); Nutt, DJ (Nutt, David J.); Orford, J (Orford, Jim); Pryce, R (Pryce, Rob); Purves, R (Purves, Richard); Reith, G (Reith, Gerda); Roberts, A (Roberts, Amanda); Roberts, E (Roberts, Emmett); Roderique-Davies, G (Roderique-Davies, Gareth); Rogers, J (Rogers, Jim); Rogers, RD (Rogers, Robert D.); Sharman, S (Sharman, Stephen); Strang, J (Strang, John); Tunney, R (Tunney, Richard); Turner, J (Turner, John); West, R (West, Robert); Zendle, D (Zendle, David)

Source: BMJ-BRITISH MEDICAL JOURNAL **Volume:** 370 **Article Number:** m2613 **DOI:** 10.1136/bmj.m2613 **Published:** JUL 1 2020

Accession Number: WOS:000548489100006

PubMed ID: 32611591

Author Identifiers:

Author	Web of Science ResearcherID	ORCID Number
McManus, Sally	S-4697-2019	0000-0003-2711-0819
James, Richard	AAT-8206-2020	0000-0002-6644-7011
McDaid, David	E-5959-2014	0000-0003-0744-2664
Roberts, Emmert		0000-0002-4152-5570
Tunney, Richard		0000-0003-4673-757X
Wardle, Heather		0000-0003-1361-3706
Strang, John	H-5460-2011	0000-0002-5413-2725

ISSN: 1756-1833

Record 5 of 189

Title: Gambling: Academics call for statutory levy to reduce harm

Author(s): Wise, J (Wise, Jacqui)

Source: BMJ-BRITISH MEDICAL JOURNAL **Volume:** 370 **Article Number:** m2600 **DOI:** 10.1136/bmj.m2600 **Published:** JUL 1 2020

Accession Number: WOS:000548489100016

PubMed ID: 32611554

ISSN: 1756-1833

Record 6 of 189

Title: Harms associated with gambling: abbreviated systematic review protocol

Author(s): Beynon, C (Beynon, Caryl); Pearce-Smith, N (Pearce-Smith, Nicola); Clark, R (Clark, Rachel)

Source: SYSTEMATIC REVIEWS **Volume:** 9 **Issue:** 1 **Article Number:** 148 **DOI:** 10.1186/s13643-020-01397-4 **Published:** JUN 23 2020

Accession Number: WOS:000544974200002

PubMed ID: 32576286

Author Identifiers:

Author	Web of Science ResearcherID	ORCID Number
Beynon, Caryl		0000-0002-2188-1500

eISSN: 2046-4053

Record 7 of 189

Title: Prevalence of gambling behaviours and their associations with socioemotional harm among 11-16 year olds in Wales: findings from the School Health Research Network survey

Author(s): Melendez-Torres, GJ (Melendez-Torres, G. J.); Anthony, RE (Anthony, Rebecca E.); Hewitt, G (Hewitt, Gillian); Murphy, S (Murphy, Simon); Moore, GF (Moore, Graham F.)

Source: EUROPEAN JOURNAL OF PUBLIC HEALTH **Volume:** 30 **Issue:** 3 **Pages:** 432-438 **DOI:** 10.1093/eurpub/ckz176 **Published:** JUN 2020

Accession Number: WOS:000544269000010

PubMed ID: 31580438

Author Identifiers:

Author	Web of Science ResearcherID	ORCID Number
Anthony, Rebecca		0000-0001-9503-9562

ISSN: 1101-1262

eISSN: 1464-360X

Record 8 of 189

Title: Prevalence of gambling behaviours and their associations with socioemotional harm among 11-16 year olds in Wales: findings from the School Health Research Network survey (vol 30, pg 432, 2020)

Author(s): Melendez-Torres, GJ (Melendez-Torres, G. J.); Anthony, RE (Anthony, Rebecca E.); Hewitt, G (Hewitt, Gillian); Murphy, S (Murphy, Simon); Moore, GF (Moore, Graham F.)

Source: EUROPEAN JOURNAL OF PUBLIC HEALTH **Volume:** 30 **Issue:** 3 **Pages:** 611-611 **DOI:** 10.1093/eurpub/ckaa010 **Published:** JUN 2020

Accession Number: WOS:000544269000041

Author Identifiers:

Author	Web of Science ResearcherID	ORCID Number
Anthony, Rebecca R	C-4999-2014	0000-0003-4933-4442

ISSN: 1101-1262

eISSN: 1464-360X

Record 9 of 189

Title: Adolescent Gambling, Gambling Expenditure and Gambling-Related Harms in Finland, 2011-2017

Author(s): Raisamo, S (Raisamo, Susanna); Kinnunen, JM (Kinnunen, Jaana M.); Pere, L (Pere, Lasse); Lindfors, P (Lindfors, Pirjo); Rimpel?, A (Rimpela, Arja)

Source: JOURNAL OF GAMBLING STUDIES **Volume:** 36 **Issue:** 2 **Pages:** 597-610 **DOI:** 10.1007/s10899-019-09892-7 **Published:** JUN 2020

Accession Number: WOS:000531806900012

PubMed ID: 31520272

ISSN: 1050-5350

eISSN: 1573-3602

Record 10 of 189

Title: Use of Self-control Strategies for Managing Gambling Habits Leads to Less Harm in Regular Gamblers

Author(s): Currie, SR (Currie, Shawn R.); Brunelle, N (Brunelle, Natacha); Dufour, M (Dufour, Magali); Flores-Pajot, MC (Flores-Pajot, Marie-Claire); Hodgins, D (Hodgins, David); Nadeau, L (Nadeau, Louise); Young, M (Young, Matthew)

Source: JOURNAL OF GAMBLING STUDIES **Volume:** 36 **Issue:** 2 **Pages:** 685-698 **DOI:** 10.1007/s10899-019-09918-0 **Published:** JUN 2020

Accession Number: WOS:000531806900017

PubMed ID: 31828695

Author Identifiers:

Author	Web of Science ResearcherID	ORCID Number
Flores-Pajot, Marie-Claire		0000-0002-7930-7472

ISSN: 1050-5350

eISSN: 1573-3602

Record 11 of 189

Title: Measuring Gambling Harm: The Influence of Response Scaling on Estimates and the Distribution of Harm Across PGSI Categories

Author(s): Delfabbro, P (Delfabbro, Paul); Georgiou, N (Georgiou, Neophytos); King, DL (King, Daniel L.)

Source: JOURNAL OF GAMBLING STUDIES **DOI:** 10.1007/s10899-020-09954-1 **Early Access Date:** MAY 2020

Accession Number: WOS:000533840200001

PubMed ID: 32424665

Author Identifiers:

Author	Web of Science ResearcherID	ORCID Number
King, Daniel	D-7357-2013	0000-0002-1762-2581
Georgiou, Neophytos		0000-0002-7868-8250

ISSN: 1050-5350

eISSN: 1573-3602

Record 12 of 189

Title: Exploring the prevalence of gambling harm among active duty military personnel: a systematic scoping review

Author(s): Paterson, M (Paterson, Marisa); Whitty, M (Whitty, Megan); Leslie, P (Leslie, Patrick)

Source: JOURNAL OF GAMBLING STUDIES **DOI:** 10.1007/s10899-020-09951-4 **Early Access Date:** MAY 2020

Accession Number: WOS:000532879400001

PubMed ID: 32410048

Author Identifiers:

Author	Web of Science ResearcherID	ORCID Number
Leslie, Patrick		0000-0002-9218-8981
Whitty, Megan		0000-0003-2628-5203

ISSN: 1050-5350

eISSN: 1573-3602

Record 13 of 189

Title: An overview of digital and online strategies to reduce gambling harm

Author(s): Paterson, M (Paterson, Marisa); Whitty, M (Whitty, Megan); Boyer, C (Boyer, Charlotte)

Source: HEALTH PROMOTION JOURNAL OF AUSTRALIA **DOI:** 10.1002/hpja.341 **Early Access Date:** MAY 2020

Accession Number: WOS:000530743900001

PubMed ID: 32246884

Author Identifiers:

Author	Web of Science ResearcherID	ORCID Number
Paterson, Marisa		0000-0001-9989-3467
Whitty, Megan		0000-0003-2628-5203

ISSN: 1036-1073

eISSN: 2201-1617

Record 14 of 189

Title: Don't Say the 'P' Word: Problem Gambling Is More than Harm

Author(s): Delfabbro, P (Delfabbro, Paul); King, DL (King, Daniel L.)

Source: INTERNATIONAL JOURNAL OF MENTAL HEALTH AND ADDICTION **Volume:** 18 **Issue:** 3 **Pages:** 835-843 **DOI:** 10.1007/s11469-020-00274-4 **Early Access Date:** APR 2020 **Published:** JUN 2020

Accession Number: WOS:000529878800001

Author Identifiers:

Author	Web of Science ResearcherID	ORCID Number
King, Daniel	D-7357-2013	0000-0002-1762-2581

ISSN: 1557-1874

eISSN: 1557-1882

Record 15 of 189

Title: Predictors of Strategy Engagement for the Prevention and Reduction of Gambling Harm: a Prospective Application of the Theory of Planned Behaviour

Author(s): Bagot, KL (Bagot, Kathleen L.); Cheetham, A (Cheetham, Alison); Lubman, DI (Lubman, Dan, I); Rodda, SN (Rodda, Simone N.)

Source: INTERNATIONAL JOURNAL OF MENTAL HEALTH AND ADDICTION **DOI:** 10.1007/s11469-020-00265-5 **Early Access Date:** APR 2020

Accession Number: WOS:000523380500002

ISSN: 1557-1874

eISSN: 1557-1882

Record 16 of 189

Title: Commentary on Nilsson et al. (2019): The development of effective interventions for concerned significant others affected by gambling harms

Author(s): Dowling, NA (Dowling, Nicki A.)

Source: ADDICTION **Volume:** 115 **Issue:** 7 **Pages:** 1343-1344 **DOI:** 10.1111/add.15026 **Early Access Date:** MAR 2020 **Published:** JUL 2020

Accession Number: WOS:000520770300001

PubMed ID: 32196805

ISSN: 0965-2140

eISSN: 1360-0443

Record 17 of 189

Title: A public health advocacy approach for preventing and reducing gambling related harm

Author(s): David, JL (David, Jennifer L.); Thomas, SL (Thomas, Samantha L.); Randle, M (Randle, Melanie); Daube, M (Daube, Mike)

Source: AUSTRALIAN AND NEW ZEALAND JOURNAL OF PUBLIC HEALTH **Volume:** 44 **Issue:** 1 **Pages:** 14-19 **DOI:** 10.1111/1753-6405.12949 **Published:** FEB 2020

Accession Number: WOS:000510265900005

PubMed ID: 31777133

Author Identifiers:

Author	Web of Science ResearcherID	ORCID Number
Thomas, Samantha		0000-0003-1427-7775

Randle, Melanie		0000-0001-9129-1701

ISSN: 1326-0200

eISSN: 1753-6405

Record 18 of 189

Title: Tackling gambling related harms as a public health issue

Author(s): Goyder, E (Goyder, Elizabeth); Blank, L (Blank, Lindsay); Baxter, S (Baxter, Susan); van Schalkwyk, MCI (van Schalkwyk, May C. I.)

Source: LANCET PUBLIC HEALTH **Volume:** 5 **Issue:** 1 **Pages:** E14-E15 **DOI:** 10.1016/S2468-2667(19)30243-9 **Published:** JAN 2020

Accession Number: WOS:000505765200008

PubMed ID: 31831371

ISSN: 2468-2667

Record 19 of 189

Title: Gambling related harms - intensive mentoring from mentors with lived experience

Author(s): Niemczewska, A (Niemczewska, Anna); Graham, F (Graham, Frankie)

Source: PERSPECTIVES IN PUBLIC HEALTH **Volume:** 140 **Issue:** 1 **Pages:** 14-15 **Published:** JAN 2020

Accession Number: WOS:000506232700003

PubMed ID: 31913104

ISSN: 1757-9139

eISSN: 1757-9147

Record 20 of 189

Title: Gambling as social practice: a complementary approach for reducing harm?

Author(s): Gordon, R (Gordon, Ross); Reith, G (Reith, Gerda)

Source: HARM REDUCTION JOURNAL **Volume:** 16 **Issue:** 1 **Article Number:** 64 **DOI:** 10.1186/s12954-019-0342-2 **Published:** DEC 5 2019

Accession Number: WOS:000515130600001

PubMed ID: 31805952

eISSN: 1477-7517

Record 21 of 189

Title: A Behaviour Sequence Analysis of Young People and Gambling-Related Harm

Author(s): Keatley, D (Keatley, David); Parke, A (Parke, Adrian); Townsend, E (Townsend, Ellen); Markham, C (Markham, Claire); Clarke, D (Clarke, David)

Source: JOURNAL OF GAMBLING ISSUES **Issue:** 43 **Pages:** 10-28 **DOI:** 10.4309/jgi.2019.43.2 **Published:** DEC 2019

Accession Number: WOS:000529979200002

ISSN: 1910-7595

Record 22 of 189

Title: Measuring Behavioural Dependence in Gambling: A Case for Removing Harmful Consequences from the Assessment of Problem Gambling Pathology

Author(s): Browne, M (Browne, Matthew); Rockloff, MJ (Rockloff, Matthew J.)

Source: JOURNAL OF GAMBLING STUDIES **DOI:** 10.1007/s10899-019-09916-2 **Early Access Date:** NOV 2019

Accession Number: WOS:000498979700001

PubMed ID: 31776754

Author Identifiers:

Author	Web of Science ResearcherID	ORCID Number
Browne, Matthew		0000-0002-2668-6229
Rockloff, Matthew		0000-0002-0080-2690

ISSN: 1050-5350

eISSN: 1573-3602

Record 23 of 189

Title: A public health advocacy approach for preventing and reducing gambling related harm

Author(s): David, JL (David, Jennifer L.); Thomas, SL (Thomas, Samantha L.); Randle, M (Randle, Melanie); Daube, M (Daube, Mike)

Source: AUSTRALIAN AND NEW ZEALAND JOURNAL OF PUBLIC HEALTH **Volume:** 44 **Issue:** 1 **Pages:** 14-19 **DOI:** 10.1111/1753-6405.12949 **Early Access Date:** NOV 2019 **Published:** FEB 2020

Accession Number: WOS:000498788600001

PubMed ID: 31777133

Author Identifiers:

Author	Web of Science ResearcherID	ORCID Number
Thomas, Samantha		0000-0003-1427-7775
Randle, Melanie		0000-0001-9129-1701

ISSN: 1326-0200

eISSN: 1753-6405

Record 24 of 189

Title: Considering the Public Health and Reno Models: Strategic and Tactical Approaches for Dealing with Gambling-Related Harms

Author(s): Shaffer, HJ (Shaffer, Howard J.); Blaszczynski, A (Blaszczynski, Alexander); Ladouceur, R (Ladouceur, Robert)

Source: INTERNATIONAL JOURNAL OF MENTAL HEALTH AND ADDICTION **Volume:** 18 **Issue:** 3 **Pages:** 806-818 **DOI:** 10.1007/s11469-019-00149-3 **Early Access Date:** OCT 2019 **Published:** JUN 2020

Accession Number: WOS:000493362200002

ISSN: 1557-1874

eISSN: 1557-1882

Record 25 of 189

Title: Problem gambling in adolescents: what are the psychological, social and financial consequences?

Author(s): Livazovic, G (Livazovic, Goran); Bojcic, K (Bojcic, Karlo)

Source: BMC PSYCHIATRY **Volume:** 19 **Issue:** 1 **Article Number:** 308 **DOI:** 10.1186/s12888-019-2293-2 **Published:** OCT 22 2019

Accession Number: WOS:000491935800004

PubMed ID: 31640621

Author Identifiers:

Author	Web of Science ResearcherID	ORCID Number
Livazovic, Goran	AAL-6443-2020	0000-0002-0277-5534
Bojcic, Karlo		0000-0001-7901-8833

eISSN: 1471-244X

Record 26 of 189

Title: Avoiding gambling harm: An evidence-based set of safe gambling practices for consumers

Author(s): Hing, N (Hing, Nerilee); Browne, M (Browne, Matthew); Russell, AMT (Russell, Alex M. T.); Rockloff, M (Rockloff, Matthew); Rawat, V (Rawat, Vijay); Nicoll, F (Nicoll, Fiona); Smith, G (Smith, Garry)

Source: PLOS ONE **Volume:** 14 **Issue:** 10 **Article Number:** e0224083 **DOI:** 10.1371/journal.pone.0224083 **Published:** OCT 17 2019

Accession Number: WOS:000532567300089

PubMed ID: 31622430

ISSN: 1932-6203

Record 27 of 189

Title: Gambling harm: a global problem requiring global solutions

Author(s): Reith, G (Reith, Gerda); Wardle, H (Wardle, Heather); Gilmore, I (Gilmore, Ian)

Source: LANCET **Volume:** 394 **Issue:** 10205 **Pages:** 1212-1214 **DOI:** 10.1016/S0140-6736(19)31991-9 **Published:** OCT 5 2019

Accession Number: WOS:000488862500007

PubMed ID: 31443927

ISSN: 0140-6736

eISSN: 1474-547X

Record 28 of 189

Title: Gambling related harm: we lack longitudinal data

Author(s): Bramley, S (Bramley, Stephanie); Manthorpe, J (Manthorpe, Jill); Norrie, C (Norrie, Caroline)

Source: BMJ-BRITISH MEDICAL JOURNAL **Volume:** 366 **Article Number:** l5295 **DOI:** 10.1136/bmj.l5295 **Published:** SEP 4 2019

Accession Number: WOS:000485437600005

PubMed ID: 31484649

Author Identifiers:

Author	Web of Science ResearcherID	ORCID Number
Manthorpe, Jill		0000-0001-9006-1410
Bramley, Stephanie		0000-0003-2702-1672
Norrie, Caroline		0000-0001-6715-9305

ISSN: 1756-1833

Record 29 of 189

Title: Challenges in the Conceptualisation and Measurement of Gambling-Related Harm

Author(s): Delfabbro, P (Delfabbro, Paul); King, DL (King, Daniel L.)

Source: JOURNAL OF GAMBLING STUDIES **Volume:** 35 **Issue:** 3 **Pages:** 743-755 **DOI:** 10.1007/s10899-019-09844-1 **Published:** SEP 2019

Accession Number: WOS:000478751700001

PubMed ID: 30879158

Author Identifiers:

Author	Web of Science ResearcherID	ORCID Number
King, Daniel L	D-7357-2013	0000-0002-1762-2581
Delfabbro, Paul	AAB-2144-2019	

ISSN: 1050-5350

eISSN: 1573-3602

Record 30 of 189

Title: Til Debt Do Us Part: Comparing Gambling Harms Between Gamblers and Their Spouses

Author(s): Jeffrey, L (Jeffrey, Lisa); Browne, M (Browne, Matthew); Rawat, V (Rawat, Vijay); Langham, E (Langham, Erika); Li, E (Li, En); Rockloff, M (Rockloff, Matthew)

Source: JOURNAL OF GAMBLING STUDIES **Volume:** 35 **Issue:** 3 **Pages:** 1015-1034 **DOI:** 10.1007/s10899-019-09826-3 **Published:** SEP 2019

Accession Number: WOS:000478751700017

PubMed ID: 30701378

Author Identifiers:

Author	Web of Science ResearcherID	ORCID Number
Langham, Erika		0000-0002-1824-5108
Browne, Matthew		0000-0002-2668-6229
Rockloff, Matthew		0000-0002-0080-2690

ISSN: 1050-5350

eISSN: 1573-3602

Record 31 of 189

Title: Gambling related harms - community and clinical comparisons

Author(s): Angus, DJ (Angus, Douglas J.); Anjoul, F (Anjoul, Fadi); Shannon, K (Shannon, Kirsten); Blaszczynski, A (Blaszczynski, Alex)

Source: ADDICTION RESEARCH & THEORY **Volume:** 28 **Issue:** 3 **Pages:** 194-203 **DOI:** 10.1080/16066359.2019.1622001 **Early Access Date:** AUG 2019 **Published:** MAY 3 2020

Accession Number: WOS:000483774200001

Author Identifiers:

Author	Web of Science ResearcherID	ORCID Number
Angus, Douglas		0000-0001-9722-2475
Blaszczynski, Alexander		0000-0003-1476-0791

ISSN: 1606-6359

eISSN: 1476-7392

Record 32 of 189

Title: Gambling: health experts investigate scale of harm

Author(s): O'Dowd, A (O'Dowd, Adrian)

Source: BMJ-BRITISH MEDICAL JOURNAL **Volume:** 366 **Article Number:** l4742 **DOI:** 10.1136/bmj.l4742 **Published:** JUL 17 2019

Accession Number: WOS:000476871500017

PubMed ID: 31315838

ISSN: 1756-1833

Record 33 of 189

Title: Social influences normalise gambling, and gambling-related harm, amongst higher-risk gamblers

Author(s): Russell, AMT (Russell, Alex Myles Thomas); Langham, E (Langham, Erika); Hing, N (Hing, Nerilee)

Source: JOURNAL OF BEHAVIORAL ADDICTIONS **Meeting Abstract:** IO2-5 **Volume:** 8 **Pages:** 108-109 **Supplement:** 1 **Published:** JUN 2019

Accession Number: WOS:000470323600176

ISSN: 2062-5871

eISSN: 2063-5303

Record 34 of 189

Title: Avoiding harmful gambling: An evidence-based set of safe gambling practices for consumers

Author(s): Hing, N (Hing, Nerilee); Browne, M (Browne, Matthew); Russell, AMT (Russell, Alex Myles Thomas); Rockloff, M (Rockloff, Matthew); Rawat, V (Rawat, Vijay); Nicoll, F (Nicoll, Fiona); Smith, G (Smith, Garry)

Source: JOURNAL OF BEHAVIORAL ADDICTIONS **Meeting Abstract:** IO4-1 **Volume:** 8 **Pages:** 112-113 **Supplement:** 1 **Published:** JUN 2019

Accession Number: WOS:000470323600182

ISSN: 2062-5871

eISSN: 2063-5303

Record 35 of 189

Title: Problem gambling and gambling-related harms amongst esports bettors and skin gamblers

Author(s): Greer, N (Greer, Nancy)

Source: JOURNAL OF BEHAVIORAL ADDICTIONS **Meeting Abstract:** IO6-4 **Volume:** 8 **Pages:** 122-122 **Supplement:** 1 **Published:** JUN 2019

Accession Number: WOS:000470323600196

ISSN: 2062-5871

eISSN: 2063-5303

Record 36 of 189

Title: What do we know about gambling-related harm affecting migrants and migrant communities? A rapid review

Author(s): Wardle, H (Wardle, Heather); Bramley, S (Bramley, Stephanie); Norrie, C (Norrie, Caroline); Manthorpe, J (Manthorpe, Jill)

Source: ADDICTIVE BEHAVIORS **Volume:** 93 **Pages:** 180-193 **DOI:** 10.1016/j.addbeh.2019.01.017 **Published:** JUN 2019

Accession Number: WOS:000469156600028

PubMed ID: 30716593

ISSN: 0306-4603

eISSN: 1873-6327

Record 37 of 189

Title: Gambling and public health: we need policy action to prevent harm

Author(s): Wardle, H (Wardle, Heather); Reith, G (Reith, Gerda); Langham, E (Langham, Erika); Rogers, RD (Rogers, Robert D.)

Source: BMJ-BRITISH MEDICAL JOURNAL **Volume:** 365 **Article Number:** l1807 **DOI:** 10.1136/bmj.l1807 **Published:** MAY 8 2019

Accession Number: WOS:000467903100004

PubMed ID: 31068335

Author Identifiers:

Author	Web of Science ResearcherID	ORCID Number
Rogers, Robert	AAF-7621-2019	0000-0001-5010-069X
Wardle, Heather		0000-0003-1361-3706

ISSN: 1756-1833

Record 38 of 189

Title: The role of public health advocacy in preventing and reducing gambling related harm: challenges, facilitators, and opportunities for change

Author(s): David, JL (David, Jennifer L.); Thomas, SL (Thomas, Samantha L.); Randle, M (Randle, Melanie); Daube, M (Daube, Mike); Balandin, S (Balandin, Susan)

Source: ADDICTION RESEARCH & THEORY **Volume:** 27 **Issue:** 3 **Pages:** 210-219 **DOI:** 10.1080/16066359.2018.1490410 **Published:** MAY 4 2019

Accession Number: WOS:000465210700005

Author Identifiers:

Author	Web of Science ResearcherID	ORCID Number
Randle, Melanie		0000-0001-9129-1701
Thomas, Samantha		0000-0003-1427-7775
Daube, Mike		0000-0002-3479-2785

ISSN: 1606-6359

eISSN: 1476-7392

Record 39 of 189

Title: Secrets and secretive behaviours: Exploring the hidden through harmful gambling

Author(s): Fulton, C (Fulton, Crystal)

Source: LIBRARY & INFORMATION SCIENCE RESEARCH **Volume:** 41 **Issue:** 2 **Pages:** 151-157 **DOI:** 10.1016/j.lisr.2019.03.003 **Published:** APR 2019

Accession Number: WOS:000473375000008

ISSN: 0740-8188

eISSN: 1873-1848

Record 40 of 189

Title: A Multivariate Evaluation of 25 Proximal and Distal Risk-Factors for Gambling-Related Harm

Author(s): Browne, M (Browne, Matthew); Hing, N (Hing, Nerilee); Rockloff, M (Rockloff, Matthew); Russell, AMT (Russell, Alex M. T.); Greer, N (Greer, Nancy); Nicoll, F (Nicoll, Fiona); Smith, G (Smith, Garry)

Source: JOURNAL OF CLINICAL MEDICINE **Volume:** 8 **Issue:** 4 **Article Number:** 509 **DOI:** 10.3390/jcm8040509 **Published:** APR 2019

Accession Number: WOS:000467500200094

PubMed ID: 31013926

Author Identifiers:

Author	Web of Science ResearcherID	ORCID Number
Russell, Alex	E-2173-2014	0000-0002-3685-7220
Browne, Matthew		0000-0002-2668-6229
Hing, Nerilee		0000-0002-2150-9784

ISSN: 2077-0383

Record 41 of 189

Title: Women and gambling-related harm: a narrative literature review and implications for research, policy, and practice

Author(s): McCarthy, S (McCarthy, Simone); Thomas, SL (Thomas, Samantha L.); Bellringer, ME (Bellringer, Maria E.); Cassidy, R (Cassidy, Rebecca)

Source: HARM REDUCTION JOURNAL **Volume:** 16 **Article Number:** 18 **DOI:** 10.1186/s12954-019-0284-8 **Published:** MAR 4 2019

Accession Number: WOS:000460585200001

PubMed ID: 30832672

Author Identifiers:

Author	Web of Science ResearcherID	ORCID Number
McCarthy, Simone	X-5088-2019	0000-0003-2671-3511
Thomas, Samantha		0000-0003-1427-7775

ISSN: 1477-7517

Record 42 of 189

Title: Online gambling: Which specificities for harm-minimisation tools?

Author(s): Bonnaire, C (Bonnaire, C.); Barrault, S (Barrault, S.)

Source: PRATIQUES PSYCHOLOGIQUES **Volume:** 25 **Issue:** 1 **Pages:** 17-35 **DOI:** 10.1016/j.prps.2018.04.001 **Published:** MAR 2019

Accession Number: WOS:000459943000002

ISSN: 1269-1763

Record 43 of 189

Title: Effects of prevention and harm reduction interventions on gambling behaviours and gambling related harm: An umbrella review

Author(s): McMahon, N (McMahon, Naoimh); Thomson, K (Thomson, Katie); Kaner, E (Kaner, Eileen); Bambra, C (Bambra, Clare)

Source: ADDICTIVE BEHAVIORS **Volume:** 90 **Pages:** 380-388 **DOI:** 10.1016/j.addbeh.2018.11.048 **Published:** MAR 2019

Accession Number: WOS:000456900000058

PubMed ID: 30529994

Author Identifiers:

Author	Web of Science ResearcherID	ORCID Number
Thomson, Katie		0000-0002-9614-728X
McMahon, Naoimh		0000-0001-6319-2263

ISSN: 0306-4603

eISSN: 1873-6327

Record 44 of 189

Title: A mapping review of research on gambling harm in three regulatory environments

Author(s): Baxter, DG (Baxter, David G.); Hilbrecht, M (Hilbrecht, Margo); Wheaton, CTJ (Wheaton, Cameron T. J.)

Source: HARM REDUCTION JOURNAL **Volume:** 16 **Article Number:** 12 **DOI:** 10.1186/s12954-018-0265-3 **Published:** FEB 8 2019

Accession Number: WOS:000458182400001

PubMed ID: 30736817

Author Identifiers:

Author	Web of Science ResearcherID	ORCID Number
Baxter, David		0000-0001-5235-6728
Hilbrecht, Margo		0000-0002-7615-7595

ISSN: 1477-7517

Record 45 of 189

Title: The nature of gambling-related harm for adults with health and social care needs: an exploratory study of the views of key informants

Author(s): Bramley, S (Bramley, Stephanie); Norrie, C (Norrie, Caroline); Manthorpe, J (Manthorpe, Jill)

Source: PRIMARY HEALTH CARE RESEARCH AND DEVELOPMENT **Volume:** 20 **Article Number:** UNSP e115 **DOI:** 10.1017/S1463423619000549 **Published:** 2019

Accession Number: WOS:000485494300001

PubMed ID: 32800002

Author Identifiers:

Author	Web of Science ResearcherID	ORCID Number
Norrie, Caroline		0000-0001-6715-9305
Manthorpe, Jill		0000-0001-9006-1410
Bramley, Stephanie		0000-0003-2702-1672

ISSN: 1463-4236

eISSN: 1477-1128

Record 46 of 189

Title: Decision-Making Measured by the Iowa Gambling Task in Patients with Alcohol Use Disorders Choosing Harm Reduction versus Relapse Prevention Program

Author(s): Briere, M (Briere, Marie); Tocanier, L (Tocanier, Laure); Allain, P (Allain, Phillippe); Le Gal, D (Le Gal, Dewi); Allet, G (Allet, Guillaume); Gorwood, P (Gorwood, Phillip); Gohier, B (Gohier, Benedicte)

Source: EUROPEAN ADDICTION RESEARCH **Volume:** 25 **Issue:** 4 **Pages:** 182-190 **DOI:** 10.1159/000499709 **Published:** 2019

Accession Number: WOS:000471954700003

PubMed ID: 31039565

Author Identifiers:

Author	Web of Science ResearcherID	ORCID Number
ALLAIN, Philiooe		0000-0003-0668-0986

ISSN: 1022-6877

eISSN: 1421-9891

Record 47 of 189

Title: Gambling disorder in adolescents: what do we know about this social problem and its consequences?

Author(s): Ferrara, P (Ferrara, Pietro); Franceschini, G (Franceschini, Giulia); Corsello, G (Corsello, Giovanni)

Source: ITALIAN JOURNAL OF PEDIATRICS **Volume:** 44 **Article Number:** 146 **DOI:** 10.1186/s13052-018-0592-8 **Published:** DEC 4 2018

Accession Number: WOS:000452329200001

PubMed ID: 30514334

Author Identifiers:

Author	Web of Science ResearcherID	ORCID Number
FERRARA, Pietro		0000-0001-9449-3464

ISSN: 1720-8424

eISSN: 1824-7288

Record 48 of 189

Title: An empirical review of gambling expansion and gambling-related harm

Author(s): LaPlante, DA (LaPlante, Debi A.); Gray, HM (Gray, Heather M.); Williams, PM (Williams, Pat M.); Nelson, SE (Nelson, Sarah E.)

Source: SUCHT-ZEITSCHRIFT FUR WISSENSCHAFT UND PRAXIS **Volume:** 64 **Issue:** 5-6 **Special Issue:** SI **Pages:** 295-306 **DOI:** 10.1024/0939-5911/a000563 **Published:** DEC 2018

Accession Number: WOS:000457208500006

Author Identifiers:

Author	Web of Science ResearcherID	ORCID Number
Gray, Heather		0000-0001-8886-5989
Williams, Pat		0000-0003-3732-0510

ISSN: 0939-5911

eISSN: 1664-2856

Record 49 of 189

Title: Social influences normalize gambling-related harm among higher risk gamblers

Author(s): Russell, AMT (Russell, Alex M. T.); Langham, E (Langham, Erika); Hing, N (Hing, Nerilee)

Source: JOURNAL OF BEHAVIORAL ADDICTIONS **Volume:** 7 **Issue:** 4 **Pages:** 1100-1111 **DOI:** 10.1556/2006.7.2018.139 **Published:** DEC 2018

Accession Number: WOS:000454636900031

PubMed ID: 30596469

ISSN: 2062-5871

eISSN: 2063-5303

Record 50 of 189

Title: BEHAVIORAL ECONOMICS AND GAMBLING: A NEW PARADIGM FOR APPROACHING HARM-MINIMIZATION

Author(s): Gainsbury, SM (Gainsbury, Sally M.); Tobias-Webb, J (Tobias-Webb, Juliette); Slonim, R (Slonim, Robert)

Source: GAMING LAW REVIEW-ECONOMICS REGULATION COMPLIANCE AND POLICY **Volume:** 22 **Issue:** 10 **Pages:** 608-617 **DOI:** 10.1089/glr2.2018.22106 **Published:** DEC 1 2018

Accession Number: WOS:000454088800002

ISSN: 2572-5300

eISSN: 2572-5327

Record 51 of 189

Title: Where Lies the Harm in Lottery Gambling? A Portrait of Gambling Practices and Associated Problems

Author(s): Costes, JM (Costes, Jean-Michel); Kairouz, S (Kairouz, Sylvia); Monson, E (Monson, Eva); Eroukmanoff, V (Eroukmanoff, Vincent)

Source: JOURNAL OF GAMBLING STUDIES **Volume:** 34 **Issue:** 4 **Pages:** 1293-1311 **DOI:** 10.1007/s10899-018-9761-3 **Published:** DEC 2018

Accession Number: WOS:000447811800012

PubMed ID: 29536292

Author Identifiers:

Author	Web of Science ResearcherID	ORCID Number
Kairouz, Sylvia		0000-0002-8788-4456

ISSN: 1050-5350

eISSN: 1573-3602

Record 52 of 189

Title: A BRIEF OVERVIEW OF THE AUSTRIAN GAMBLING REGULATION AND IMPLICATIONS FOR PLAYER PROTECTION AND HARM MINIMIZATION

Author(s): Malischnig, D (Malischnig, Doris); Griffiths, MD (Griffiths, Mark D.); Auer, M (Auer, Michael)

Source: GAMING LAW REVIEW-ECONOMICS REGULATION COMPLIANCE AND POLICY **Volume:** 22 **Issue:** 9 **Pages:** 564-567 **DOI:** 10.1089/glr2.2018.2297 **Published:** NOV 1 2018

Accession Number: WOS:000450419900005

ISSN: 2572-5300

eISSN: 2572-5327

Record 53 of 189

Title: The demand for gambling in Italian regions and its distributional consequences

Author(s): Gandullia, L (Gandullia, Luca); Leporatti, L (Leporatti, Lucia)

Source: PAPERS IN REGIONAL SCIENCE **Volume:** 97 **Issue:** 4 **Pages:** 1203-+ **DOI:** 10.1111/pirs.12302 **Published:** NOV 2018

Accession Number: WOS:000449522700017

Author Identifiers:

Author	Web of Science ResearcherID	ORCID Number
LEPORATTI, LUCIA		0000-0003-4186-908X
GANDULLIA, Luca		0000-0003-2545-9742

ISSN: 1056-8190

eISSN: 1435-5957

Record 54 of 189

Title: A Comparison of Online Versus Offline Gambling Harm in Portuguese Pathological Gamblers: An Empirical Study

Author(s): Hubert, P (Hubert, Pedro); Griffiths, MD (Griffiths, Mark D.)

Source: INTERNATIONAL JOURNAL OF MENTAL HEALTH AND ADDICTION **Volume:** 16 **Issue:** 5 **Pages:** 1219-1237 **DOI:** 10.1007/s11469-017-9846-8 **Published:** OCT 2018

Accession Number: WOS:000447735400011

PubMed ID: 30416402

ISSN: 1557-1874

eISSN: 1557-1882

Record 55 of 189

Title: Harm caused by gambling among non-problem gamblers: Is a whole-of-population approach undervalued?

Author(s): Raisamo, S (Raisamo, Susanna)

Source: SCANDINAVIAN JOURNAL OF PUBLIC HEALTH **Volume:** 46 **Issue:** 5 **Pages:** 503-504 **DOI:** 10.1177/1403494817744073 **Published:** JUL 2018

Accession Number: WOS:000438611100001

PubMed ID: 29173046

ISSN: 1403-4948

eISSN: 1651-1905

Record 56 of 189

Title: Prevalence of gambling-related harm provides evidence for the prevention paradox

Author(s): Browne, M (Browne, Matthew); Rockloff, MJ (Rockloff, Matthew J.)

Source: JOURNAL OF BEHAVIORAL ADDICTIONS **Volume:** 7 **Issue:** 2 **Pages:** 410-422 **DOI:** 10.1556/2006.7.2018.41 **Published:** JUN 2018

Accession Number: WOS:000437461800024

PubMed ID: 29788761

Author Identifiers:

Author	Web of Science ResearcherID	ORCID Number
Browne, Matthew		0000-0002-2668-6229

ISSN: 2062-5871

eISSN: 2063-5303

Record 57 of 189

Title: Gambling participation, gambling habits, gambling-related harm, and opinions on gambling advertising in Finland in 2016

Author(s): Salonen, AH (Salonen, Anne H.); Hellman, M (Hellman, Matilda); Latvala, T (Latvala, Tiina); Castren, S (Castren, Sari)

Source: NORDIC STUDIES ON ALCOHOL AND DRUGS **Volume:** 35 **Issue:** 3 **Pages:** 215-234 **DOI:** 10.1177/1455072518765875 **Published:** JUN 2018

Accession Number: WOS:000435962100006

Author Identifiers:

Author	Web of Science ResearcherID	ORCID Number
Hellman, Matilda		0000-0001-8884-8601

ISSN: 1455-0725

eISSN: 1458-6126

Record 58 of 189

Title: Validation of the Short Gambling Harm Screen (SGHS): A Tool for Assessment of Harms from Gambling

Author(s): Browne, M (Browne, Matthew); Goodwin, BC (Goodwin, Belinda C.); Rockloff, MJ (Rockloff, Matthew J.)

Source: JOURNAL OF GAMBLING STUDIES **Volume:** 34 **Issue:** 2 **Pages:** 499-512 **DOI:** 10.1007/s10899-017-9698-y **Published:** JUN 2018

Accession Number: WOS:000431674600010

PubMed ID: 28578519

Author Identifiers:

Author	Web of Science ResearcherID	ORCID Number
Browne, Matthew		0000-0002-2668-6229

ISSN: 1050-5350

eISSN: 1573-3602

Record 59 of 189

Title: Crossover Effects of Protective Behavioural Strategies for Drinking on Gambling Consequences Among College Gamblers With Alcohol or Drug Abuse

Author(s): Granato, H (Granato, Hollie); Luk, JW (Luk, Jeremy W.); Paves, A (Paves, Andrew); Geisner, IM (Geisner, Irene M.); Cronce, JM (Cronce, Jessica M.); Kilmer, JR (Kilmer, Jason R.); Lostutter, TW (Lostutter, Ty W.); Larimer, ME (Larimer, Mary E.)

Source: JOURNAL OF GAMBLING ISSUES **Issue:** 38 **Special Issue:** SI **Pages:** 190-202 **DOI:** 10.4309/jgi.2018.38.10 **Published:** MAY 2018

Accession Number: WOS:000438728500010

Author Identifiers:

Author	Web of Science ResearcherID	ORCID Number
Luk, Jeremy		0000-0002-9061-1555

ISSN: 1910-7595

Record 60 of 189

Title: Women's gambling behaviour, product preferences, and perceptions of product harm: differences by age and gambling risk status

Author(s): McCarthy, S (McCarthy, Simone); Thomas, SL (Thomas, Samantha L.); Randle, M (Randle, Melanie); Bestman, A (Bestman, Amy); Pitt, H (Pitt, Hannah); Cowlishaw, S (Cowlishaw, Sean); Daube, M (Daube, Mike)

Source: HARM REDUCTION JOURNAL **Volume:** 15 **Article Number:** 22 **DOI:** 10.1186/s12954-018-0227-9 **Published:** APR 24 2018

Accession Number: WOS:000431052100001

PubMed ID: 29690876

Author Identifiers:

Author	Web of Science ResearcherID	ORCID Number
Pitt, Hannah	X-9882-2019	0000-0002-4259-6186
McCarthy, Simone	X-5088-2019	0000-0003-2671-3511
Thomas, Samantha		0000-0003-1427-7775
Bestman, Amy		0000-0003-1269-2123
Daube, Mike		0000-0002-3479-2785
Randle, Melanie		0000-0001-9129-1701

ISSN: 1477-7517

Record 61 of 189

Title: The Case for Using Personally Relevant and Emotionally Stimulating Gambling Messages as a Gambling Harm-Minimisation Strategy

Author(s): Harris, A (Harris, Andrew); Parke, A (Parke, Adrian); Griffiths, MD (Griffiths, Mark D.)

Source: INTERNATIONAL JOURNAL OF MENTAL HEALTH AND ADDICTION **Volume:** 16 **Issue:** 2 **Pages:** 266-275 **DOI:** 10.1007/s11469-016-9698-7 **Published:** APR 2018

Accession Number: WOS:000429946600002

PubMed ID: 29670498

Author Identifiers:

Author	Web of Science ResearcherID	ORCID Number
Parke, Adrian	K-4354-2015	0000-0002-5242-6308
Griffiths, Mark		0000-0001-8880-6524

Harris, Andrew		0000-0001-9627-4900

ISSN: 1557-1874

eISSN: 1557-1882

Record 62 of 189

Title: A process evaluation of the 'Aware' and 'Supportive Communities' gambling harm-minimisation programmes in New Zealand

Author(s): Kolandai-Matchett, K (Kolandai-Matchett, Komathi); Bellringer, M (Bellringer, Maria); Landon, J (Landon, Jason); Abbott, M (Abbott, Max)

Source: EUROPEAN JOURNAL OF PUBLIC HEALTH **Volume:** 28 **Issue:** 2 **Pages:** 369-376 **DOI:** 10.1093/eurpub/ckx120 **Published:** APR 2018

Accession Number: WOS:000429036800033

PubMed ID: 29020385

Author Identifiers:

Author	Web of Science ResearcherID	ORCID Number
Landon, Jason	AAD-3951-2019	
Kolandai-Matchett, Komathi	AAK-7059-2020	

ISSN: 1101-1262

eISSN: 1464-360X

Record 63 of 189

Title: Gambling Participation, Expenditure and Risk of Harm in Australia, 1997-1998 and 2010-2011

Author(s): Armstrong, AR (Armstrong, Andrew Richard); Thomas, A (Thomas, Anna); Abbott, M (Abbott, Max)

Source: JOURNAL OF GAMBLING STUDIES **Volume:** 34 **Issue:** 1 **Pages:** 255-274 **DOI:** 10.1007/s10899-017-9708-0 **Published:** MAR 2018

Accession Number: WOS:000426564900016

PubMed ID: 28840412

ISSN: 1050-5350

eISSN: 1573-3602

Record 64 of 189

Title: Gambling-related harms and homelessness: findings from a scoping review

Author(s): Bramley, S (Bramley, Stephanie); Norrie, C (Norrie, Caroline); Manthorpe, J (Manthorpe, Jill)

Source: HOUSING CARE AND SUPPORT **Volume:** 21 **Issue:** 1 **Pages:** 26-39 **DOI:** 10.1108/HCS-02-2018-0003 **Published:** 2018

Accession Number: WOS:000431686800003

Author Identifiers:

Author	Web of Science ResearcherID	ORCID Number
Norrie, Caroline		0000-0001-6715-9305
Bramley, Stephanie		0000-0003-2702-1672
Manthorpe, Jill		0000-0001-9006-1410

ISSN: 1460-8790

eISSN: 2042-8375

Record 65 of 189

Title: Understanding end-user perspectives to enhance perceived value uptake of harm-minimization tools: considering gambler's views of a pre-commitment system

Author(s): Gainsbury, SM (Gainsbury, Sally M.); Jakob, L (Jakob, Laura); Aro, D (Aro, David)

Source: INTERNATIONAL GAMBLING STUDIES **Volume:** 18 **Issue:** 1 **Pages:** 22-38 **DOI:** 10.1080/14459795.2017.1370723 **Published:** 2018

Accession Number: WOS:000423752500002

ISSN: 1445-9795

eISSN: 1479-4276

Record 66 of 189

Title: Identifying risk and mitigating gambling-related harm in online poker

Author(s): Parke, A (Parke, Adrian); Griffiths, MD (Griffiths, Mark D.)

Source: JOURNAL OF RISK RESEARCH **Volume:** 21 **Issue:** 3 **Pages:** 269-289 **DOI:** 10.1080/13669877.2016.1200657 **Published:** 2018

Accession Number: WOS:000427729500001

Author Identifiers:

Author	Web of Science ResearcherID	ORCID Number
Parke, Adrian	K-4354-2015	0000-0002-5242-6308
Griffiths, Mark		0000-0001-8880-6524

ISSN: 1366-9877

eISSN: 1466-4461

Record 67 of 189

Title: Loyalty programmes in the gambling industry: potentials for harm and possibilities for harm-minimization

Author(s): Wohl, MJA (Wohl, Michael J. A.)

Source: INTERNATIONAL GAMBLING STUDIES **Volume:** 18 **Issue:** 3 **Pages:** 495-511 **DOI:** 10.1080/14459795.2018.1480649 **Published:** 2018

Accession Number: WOS:000446111100009

ISSN: 1445-9795

eISSN: 1479-4276

Record 68 of 189

Title: Configurations of gambling change and harm: qualitative findings from the Swedish longitudinal gambling study (Swelogs)

Author(s): Samuelsson, E (Samuelsson, Eva); Sundqvist, K (Sundqvist, Kristina); Binde, P (Binde, Per)

Source: ADDICTION RESEARCH & THEORY **Volume:** 26 **Issue:** 6 **Pages:** 514-524 **DOI:** 10.1080/16066359.2018.1448390 **Published:** 2018

Accession Number: WOS:000445288300011

Author Identifiers:

Author	Web of Science ResearcherID	ORCID Number
Samuelsson, Eva	M-8975-2019	0000-0002-0856-9854

ISSN: 1606-6359

eISSN: 1476-7392

Record 69 of 189

Title: Casino Gambling and the Family: Exploring the Connections and Identifying Consequences

Author(s): Anderson, TL (Anderson, Tammy L.); Rempusheski, VF (Rempusheski, Veronica F.); Leedy, KN (Leedy, Kelly N.)

Source: DEVIANT BEHAVIOR **Volume:** 39 **Issue:** 9 **Pages:** 1109-1119 **DOI:** 10.1080/01639625.2017.1409484 **Published:** 2018

Accession Number: WOS:000433135100001

ISSN: 0163-9625

eISSN: 1521-0456

Record 70 of 189

Title: On gambling research, social science and the consequences of commercial gambling

Author(s): Livingstone, C (Livingstone, Charles); Adams, P (Adams, Peter); Cassidy, R (Cassidy, Rebecca); Markham, F (Markham, Francis); Reith, G (Reith, Gerda); Rintoul, A (Rintoul, Angela); Schull, ND (Schuell, Natasha Dow); Woolley, R (Woolley, Richard); Young, M (Young, Martin)

Source: INTERNATIONAL GAMBLING STUDIES **Volume:** 18 **Issue:** 1 **Pages:** 56-68 **DOI:** 10.1080/14459795.2017.1377748 **Published:** 2018

Accession Number: WOS:000423752500004

Author Identifiers:

Author	Web of Science ResearcherID	ORCID Number
Markham, Francis	H-5471-2019	0000-0002-4266-2569
Rintoul, Angela		0000-0003-4159-8814
Livingstone, Charles		0000-0003-3946-2061
Adams, Peter		0000-0002-3237-0108
Woolley, Richard		0000-0003-1247-8776

ISSN: 1445-9795

eISSN: 1479-4276

Record 71 of 189

Title: Association between gambling harms and game types: Finnish population study

Author(s): Castren, S (Castren, Sari); Perhoniemi, R (Perhoniemi, Riku); Kontto, J (Kontto, Jukka); Alho, H (Alho, Hannu); Salonen, AH (Salonen, Anne H.)

Source: INTERNATIONAL GAMBLING STUDIES **Volume:** 18 **Issue:** 1 **Pages:** 124-142 **DOI:** 10.1080/14459795.2017.1388830 **Published:** 2018

Accession Number: WOS:000423752500008

Author Identifiers:

Author	Web of Science ResearcherID	ORCID Number
Kontto, Jukka		0000-0003-3899-9852

ISSN: 1445-9795

eISSN: 1479-4276

Record 72 of 189

Title: Gambling and Gambling Harm in New Zealand: a 28-Year Case Study

Author(s): Abbott, M (Abbott, Max)

Source: INTERNATIONAL JOURNAL OF MENTAL HEALTH AND ADDICTION **Volume:** 15 **Issue:** 6 **Pages:** 1221-1241 **DOI:** 10.1007/s11469-017-9767-6 **Published:** DEC 2017

Accession Number: WOS:000417880500008

ISSN: 1557-1874

eISSN: 1557-1882

Record 73 of 189

Title: The Unintended Normalization of Gambling: Family Identity Influences on the Adoption of Harmful Consumption Practices

Author(s): Westberg, K (Westberg, Kate); Beverland, MB (Beverland, Michael B.); Thomas, SL (Thomas, Samantha L.)

Source: JOURNAL OF MACROMARKETING **Volume:** 37 **Issue:** 4 **Special Issue:** SI **Pages:** 426-443 **DOI:** 10.1177/0276146717720979 **Published:** DEC 2017

Accession Number: WOS:000414653700008

Author Identifiers:

Author	Web of Science ResearcherID	ORCID Number
Westberg, Kate	P-7601-2019	
Thomas, Samantha		0000-0003-1427-7775

ISSN: 0276-1467

eISSN: 1552-6534

Record 74 of 189

Title: INTENSITY AND GAMBLING HARMS: EXPLORING BREADTH OF GAMBLING INVOLVEMENT AMONG ESPORTS BETTORS

Author(s): Gainsbury, SM (Gainsbury, Sally M.); Abarbanel, B (Abarbanel, Brett); Blaszczynski, A (Blaszczynski, Alex)

Source: GAMING LAW REVIEW-ECONOMICS REGULATION COMPLIANCE AND POLICY **Volume:** 21 **Issue:** 8 **Special Issue:** SI **Pages:** 610-615 **DOI:** 10.1089/glr2.2017.21812 **Published:** OCT 2017

Accession Number: WOS:000411716500008

Author Identifiers:

Author	Web of Science ResearcherID	ORCID Number
Blaszczynski, Alexander	G-2713-2013	0000-0003-1476-0791

ISSN: 2572-5300

eISSN: 2572-5327

Record 75 of 189

Title: What is the harm? Applying a public health methodology to measure the impact of gambling problems and harm on quality of life

Author(s): Browne, M (Browne, Matthew); Rawat, V (Rawat, Vijay); Greer, N (Greer, Nancy); Langham, E (Langham, Erika); Rockloff, M (Rockloff, Matthew); Hanley, C (Hanley, Christine)

Source: JOURNAL OF GAMBLING ISSUES **Issue:** 36 **Pages:** 28-50 **DOI:** 10.4309/jgi.2017.36.2 **Published:** SEP 2017

Accession Number: WOS:000419669100002

Author Identifiers:

Author	Web of Science ResearcherID	ORCID Number
Langham, Erika		0000-0002-1824-5108

ISSN: 1910-7595

Record 76 of 189

Title: The dangers of conflating gambling-related harm with disordered gambling Commentary on: Prevention paradox logic and problem gambling (Delfabbro & King, 2017)

Author(s): Browne, M (Browne, Matthew); Rockloff, MJ (Rockloff, Matthew J.)

Source: JOURNAL OF BEHAVIORAL ADDICTIONS **Volume:** 6 **Issue:** 3 **Pages:** 317-320 **DOI:** 10.1556/2006.6.2017.059 **Published:** SEP 2017

Accession Number: WOS:000411876000012

PubMed ID: 28889755

Author Identifiers:

Author	Web of Science ResearcherID	ORCID Number
Browne, Matthew		0000-0002-2668-6229

ISSN: 2062-5871

eISSN: 2063-5303

Record 77 of 189

Title: Public attitudes towards gambling product harm and harm reduction strategies: an online study of 16-88 year olds in Victoria, Australia

Author(s): Thomas, SL (Thomas, Samantha L.); Randle, M (Randle, Melanie); Bestman, A (Bestman, Amy); Pitt, H (Pitt, Hannah); Bowe, SJ (Bowe, Steven J.); Cowlishaw, S (Cowlishaw, Sean); Daube, M (Daube, Mike)

Source: HARM REDUCTION JOURNAL **Volume:** 14 **Article Number:** 49 **DOI:** 10.1186/s12954-017-0173-y **Published:** JUL 25 2017

Accession Number: WOS:000406319200001

PubMed ID: 28743300

Author Identifiers:

Author	Web of Science ResearcherID	ORCID Number

Pitt, Hannah	X-9882-2019	0000-0002-4259-6186
Bowe, Steven		0000-0003-3813-842X
Randle, Melanie		0000-0001-9129-1701
Bestman, Amy		0000-0003-1269-2123
Daube, Mike		0000-0002-3479-2785
Thomas, Samantha		0000-0003-1427-7775

ISSN: 1477-7517

Record 78 of 189

Title: Prevention paradox logic and problem gambling: Does low-risk gambling impose a greater burden of harm than high-risk gambling?

Author(s): Delfabbro, P (Delfabbro, Paul); King, D (King, Daniel)

Source: JOURNAL OF BEHAVIORAL ADDICTIONS **Volume:** 6 **Issue:** 2 **Pages:** 163-167 **DOI:** 10.1556/2006.6.2017.022 **Published:** JUN 2017

Accession Number: WOS:000404596200010

PubMed ID: 28425779

Author Identifiers:

Author	Web of Science ResearcherID	ORCID Number
Delfabbro, Paul	AAB-2144-2019	
King, Daniel L	D-7357-2013	0000-0002-1762-2581

ISSN: 2062-5871

eISSN: 2063-5303

Record 79 of 189

Title: Sense of Coherence and Gambling: Exploring the Relationship Between Sense of Coherence, Gambling Behaviour and Gambling-Related Harm

Author(s): Langham, E (Langham, Erika); Russell, AMT (Russell, Alex M. T.); Hing, N (Hing, Nerilee); Gainsbury, SM (Gainsbury, Sally M.)

Source: JOURNAL OF GAMBLING STUDIES **Volume:** 33 **Issue:** 2 **Pages:** 661-684 **DOI:** 10.1007/s10899-016-9640-8 **Published:** JUN 2017

Accession Number: WOS:000402186400021

PubMed ID: 27572488

Author Identifiers:

Author	Web of Science ResearcherID	ORCID Number
Russell, Alex	E-2173-2014	0000-0002-3685-7220
Gainsbury, Sally		0000-0002-9641-5838
Hing, Nerilee		0000-0002-2150-9784
Langham, Erika		0000-0002-1824-5108

ISSN: 1050-5350

eISSN: 1573-3602

Record 80 of 189

Title: Keynote for ICBA Conference (Haifa 2017) Behavioural tracking in gambling: Implications for responsible gambling, player protection, and harm minimization

Author(s): Griffiths, M (Griffiths, Mark)

Source: JOURNAL OF BEHAVIORAL ADDICTIONS **Meeting Abstract:** PL-04 **Volume:** 6 **Pages:** 2-2 **Supplement:** 1 **Published:** MAR 2017

Accession Number: WOS:000398224200005

ISSN: 2062-5871

eISSN: 2063-5303

Record 81 of 189

Title: Shape and size - An exploration of both intended and unintended consequences and responses of gambling within a diverse society with reference to South African women

Author(s): De Vries, L (De Vries, Linda)

Source: JOURNAL OF BEHAVIORAL ADDICTIONS **Meeting Abstract:** OP-17 **Volume:** 6 **Pages:** 10-10 **Supplement:** 1 **Published:** MAR 2017

Accession Number: WOS:000398224200023

ISSN: 2062-5871

eISSN: 2063-5303

Record 82 of 189

Title: Online gamblers opinion about harm-minimisation tools

Author(s): Caillon, J (Caillon, J.); Grall-Bronnec, M (Grall-Bronnec, M.); Romo, L (Romo, L.); Bouju, G (Bouju, G.)

Source: JOURNAL OF BEHAVIORAL ADDICTIONS **Meeting Abstract:** SH-04 **Volume:** 6 **Pages:** 63-63 **Supplement:** 1 **Published:** MAR 2017

Accession Number: WOS:000398224200137

Author Identifiers:

Author	Web of Science ResearcherID	ORCID Number
Romo, Lucia	O-1584-2019	

ISSN: 2062-5871

eISSN: 2063-5303

Record 83 of 189

Title: Breaking Bad: Comparing Gambling Harms Among Gamblers and Affected Others

Author(s): Li, E (Li, En); Browne, M (Browne, Matthew); Rawat, V (Rawat, Vijay); Langham, E (Langham, Erika); Rockloff, M (Rockloff, Matthew)

Source: JOURNAL OF GAMBLING STUDIES **Volume:** 33 **Issue:** 1 **Pages:** 223-248 **DOI:** 10.1007/s10899-016-9632-8 **Published:** MAR 2017

Accession Number: WOS:000395082100013

PubMed ID: 27443306

Author Identifiers:

Author	Web of Science ResearcherID	ORCID Number
Langham, Erika		0000-0002-1824-5108
Browne, Matthew		0000-0002-2668-6229

ISSN: 1050-5350

eISSN: 1573-3602

Record 84 of 189

Title: Gambling Harm and Crime Careers

Author(s): May-Chahal, C (May-Chahal, Corinne); Humphreys, L (Humphreys, Leslie); Clifton, A (Clifton, Alison); Francis, B (Francis, Brian); Reith, G (Reith, Gerda)

Source: JOURNAL OF GAMBLING STUDIES **Volume:** 33 **Issue:** 1 **Pages:** 65-84 **DOI:** 10.1007/s10899-016-9612-z **Published:** MAR 2017

Accession Number: WOS:000395082100005

PubMed ID: 27116232

Author Identifiers:

Author	Web of Science ResearcherID	ORCID Number
Francis, Brian J	A-1953-2011	0000-0001-7926-9085
Humphreys, Leslie		0000-0002-3756-4710

ISSN: 1050-5350

eISSN: 1573-3602

Record 85 of 189

Title: A Critical Review of the Harm-Minimisation Tools Available for Electronic Gambling

Author(s): Harris, A (Harris, Andrew); Griffiths, MD (Griffiths, Mark D.)

Source: JOURNAL OF GAMBLING STUDIES **Volume:** 33 **Issue:** 1 **Pages:** 187-221 **DOI:** 10.1007/s10899-016-9624-8 **Published:** MAR 2017

Accession Number: WOS:000395082100012

PubMed ID: 27289237

Author Identifiers:

Author	Web of Science ResearcherID	ORCID Number
Griffiths, Mark		0000-0001-8880-6524

ISSN: 1050-5350

eISSN: 1573-3602

Record 86 of 189

Title: Factors that influence children's gambling attitudes and consumption intentions: lessons for gambling harm prevention research, policies and advocacy strategies

Author(s): Pitt, H (Pitt, Hannah); Thomas, SL (Thomas, Samantha L.); Bestman, A (Bestman, Amy); Daube, M (Daube, Mike); Derevensky, J (Derevensky, Jeffrey)

Source: HARM REDUCTION JOURNAL **Volume:** 14 **Article Number:** 11 **DOI:** 10.1186/s12954-017-0136-3 **Published:** FEB 17 2017

Accession Number: WOS:000394814600001

PubMed ID: 28212685

Author Identifiers:

Author	Web of Science ResearcherID	ORCID Number
Pitt, Hannah	X-9882-2019	0000-0002-4259-6186
Thomas, Samantha		0000-0003-1427-7775
Bestman, Amy		0000-0003-1269-2123
Daube, Mike		0000-0002-3479-2785

ISSN: 1477-7517

Record 87 of 189

Title: Attitudes towards gambling, gambling participation, and gambling-related harm: cross-sectional Finnish population studies in 2011 and 2015

Author(s): Salonen, AH (Salonen, Anne H.); Alho, H (Alho, Hannu); Castren, S (Castren, Sari)

Source: BMC PUBLIC HEALTH **Volume:** 17 **Article Number:** 122 **DOI:** 10.1186/s12889-017-4056-7 **Published:** JAN 26 2017

Accession Number: WOS:000392915000001

PubMed ID: 28122531

ISSN: 1471-2458

Record 88 of 189

Title: Me, myself and money: having a financially focused self-concept and its consequences for disordered gambling

Author(s): Tabri, N (Tabri, Nassim); Wohl, MJA (Wohl, Michael J. A.); Eddy, KT (Eddy, Kamryn T.); Thomas, JJ (Thomas, Jennifer J.)

Source: INTERNATIONAL GAMBLING STUDIES **Volume:** 17 **Issue:** 1 **Pages:** 30-50 **DOI:** 10.1080/14459795.2016.1252414 **Published:** 2017

Accession Number: WOS:000396684100003

Author Identifiers:

Author	Web of Science ResearcherID	ORCID Number
Thomas, Jennifer J.	AAM-4096-2020	
Tabri, Nassim	Y-4094-2018	0000-0002-7085-9350
Tabri, Nassim	AAB-4218-2019	0000-0002-7085-9350

ISSN: 1445-9795

eISSN: 1479-4276

Record 89 of 189

Title: Responsible gambling codes of conduct: lack of harm minimisation intervention in the context of venue self-regulation

Author(s): Rintoul, A (Rintoul, Angela); Deblaquiere, J (Deblaquiere, Julie); Thomas, A (Thomas, Anna)

Source: ADDICTION RESEARCH & THEORY **Volume:** 25 **Issue:** 6 **Pages:** 451-461 **DOI:** 10.1080/16066359.2017.1314465 **Published:** 2017

Accession Number: WOS:000413961400004

Author Identifiers:

Author	Web of Science ResearcherID	ORCID Number
Rintoul, Angela		0000-0003-4159-8814

ISSN: 1606-6359

eISSN: 1476-7392

Record 90 of 189

Title: Assigning responsibility for gambling-related harm: scrutinizing processes of direct and indirect consumer responsibilization of gamblers in Sweden

Author(s): Alexius, S (Alexius, Susanna)

Source: ADDICTION RESEARCH & THEORY **Volume:** 25 **Issue:** 6 **Pages:** 462-475 **DOI:** 10.1080/16066359.2017.1321739 **Published:** 2017

Accession Number: WOS:000413961400005

ISSN: 1606-6359

eISSN: 1476-7392

Record 91 of 189

Title: Harm reduction in gambling: a systematic review of industry strategies

Author(s): Tanner, J (Tanner, Jessica); Drawson, AS (Drawson, Alexandra S.); Mushquash, CJ (Mushquash, Christopher J.); Mushquash, AR (Mushquash, Aislin R.); Mazmanian, D (Mazmanian, Dwight)

Source: ADDICTION RESEARCH & THEORY **Volume:** 25 **Issue:** 6 **Pages:** 485-494 **DOI:** 10.1080/16066359.2017.1310204 **Published:** 2017

Accession Number: WOS:000413961400007

ISSN: 1606-6359

eISSN: 1476-7392

Record 92 of 189

Title: Mapping the proportional distribution of gambling-related harms in a clinical and community sample

Author(s): Shannon, K (Shannon, K.); Anjoul, F (Anjoul, F.); Blaszczynski, A (Blaszczynski, A.)

Source: INTERNATIONAL GAMBLING STUDIES **Volume:** 17 **Issue:** 3 **Pages:** 366-385 **DOI:** 10.1080/14459795.2017.1333131 **Published:** 2017

Accession Number: WOS:000410770900002

Author Identifiers:

Author	Web of Science ResearcherID	ORCID Number
Blaszczynski, Alexander	G-2713-2013	0000-0003-1476-0791

ISSN: 1445-9795

eISSN: 1479-4276

Record 93 of 189

Title: A population-level metric for gambling-related harm

Author(s): Browne, M (Browne, Matthew); Greer, N (Greer, Nancy); Rawat, V (Rawat, Vijay); Rockloff, M (Rockloff, Matthew)

Source: INTERNATIONAL GAMBLING STUDIES **Volume:** 17 **Issue:** 2 **Pages:** 163-175 **DOI:** 10.1080/14459795.2017.1304973 **Published:** 2017

Accession Number: WOS:000406686300002

Author Identifiers:

Author	Web of Science ResearcherID	ORCID Number
Rockloff, Matthew		0000-0002-0080-2690
Browne, Matthew		0000-0002-2668-6229

ISSN: 1445-9795

eISSN: 1479-4276

Record 94 of 189

Title: The family exclusion order as a harm-minimisation measure for casino gambling: the case of Singapore

Author(s): Goh, ECL (Goh, Esther C. L.); Ng, V (Ng, Vincent); Yeoh, BSA (Yeoh, Brenda S. A.)

Source: INTERNATIONAL GAMBLING STUDIES **Volume:** 16 **Issue:** 3 **Pages:** 373-390 **DOI:** 10.1080/14459795.2016.1211169 **Published:** DEC 2016

Accession Number: WOS:000389046000003

Author Identifiers:

Author	Web of Science ResearcherID	ORCID Number
Goh, Esther C L	K-8511-2012	0000-0002-0235-2363
Yeoh, Brenda		0000-0002-0240-3175

ISSN: 1445-9795

eISSN: 1479-4276

Record 95 of 189

Title: The Effects of Pop-up Harm Minimisation Messages on Electronic Gaming Machine Gambling Behaviour in New Zealand

Author(s): du Preez, KP (du Preez, Katie Palmer); Landon, J (Landon, Jason); Bellringer, M (Bellringer, Maria); Garrett, N (Garrett, Nick); Abbott, M (Abbott, Max)

Source: JOURNAL OF GAMBLING STUDIES **Volume:** 32 **Issue:** 4 **Pages:** 1115-1126 **DOI:** 10.1007/s10899-016-9603-0 **Published:** DEC 2016

Accession Number: WOS:000387615100005

PubMed ID: 27038467

Author Identifiers:

Author	Web of Science ResearcherID	ORCID Number
Landon, Jason	AAD-3951-2019	
Landon, Jason		0000-0002-3595-7430

ISSN: 1050-5350

eISSN: 1573-3602

Record 96 of 189

Title: Thoughts and acts of self-harm, and suicidal ideation, in online gamblers

Author(s): Lloyd, J (Lloyd, Joanne); Hawton, K (Hawton, Keith); Dutton, WH (Dutton, William H.); Geddes, JR (Geddes, John R.); Goodwin, GM (Goodwin, Guy M.); Rogers, RD (Rogers, Robert D.)

Source: INTERNATIONAL GAMBLING STUDIES **Volume:** 16 **Issue:** 3 **Pages:** 408-423 **DOI:** 10.1080/14459795.2016.1214166 **Published:** DEC 2016

Accession Number: WOS:000389046000005

Author Identifiers:

Author	Web of Science ResearcherID	ORCID Number
Rogers, Robert	AAF-7621-2019	0000-0001-5010-069X
Lloyd, Joanne		0000-0003-3891-7247

ISSN: 1445-9795

eISSN: 1479-4276

Record 97 of 189

Title: The extent and type of gambling harms for concerned significant others: A cross-sectional population study in Finland

Author(s): Salonen, AH (Salonen, Anne H.); Alho, H (Alho, Hannu); Castren, S (Castren, Sari)

Source: SCANDINAVIAN JOURNAL OF PUBLIC HEALTH **Volume:** 44 **Issue:** 8 **Pages:** 799-804 **DOI:** 10.1177/1403494816673529 **Published:** DEC 2016

Accession Number: WOS:000387502800011

PubMed ID: 28929933

ISSN: 1403-4948

eISSN: 1651-1905

Record 98 of 189

Title: BUILDING THE EVIDENCE FOR EFFECTIVE HARM REDUCTION: WHERE IS GAMBLING?

Author(s): Thomas, A (Thomas, Anna)

Source: DRUG AND ALCOHOL REVIEW **Meeting Abstract:** 103 **Volume:** 35 **Special Issue:** SI **Pages:** 70-70 **Supplement:** 1 **Published:** OCT 2016

Accession Number: WOS:000398381500192

ISSN: 0959-5236

eISSN: 1465-3362

Record 99 of 189

Title: Types of gambling and levels of harm: A UK study to assess severity of presentation in a treatment-seeking population

Author(s): Ronzitti, S (Ronzitti, Silvia); Soldini, E (Soldini, Emiliano); Lutri, V (Lutri, Vittorio); Smith, N (Smith, Neil); Clerici, M (Clerici, Massimo); Bowden-Jones, H (Bowden-Jones, Henrietta)

Source: JOURNAL OF BEHAVIORAL ADDICTIONS **Volume:** 5 **Issue:** 3 **Pages:** 439-447 **DOI:** 10.1556/2006.5.2016.068 **Published:** SEP 2016

Accession Number: WOS:000385362700008

PubMed ID: 27677350

Author Identifiers:

Author	Web of Science ResearcherID	ORCID Number
Clerici, Massimo	U-3074-2019	0000-0001-8769-6474
Soldini, Emiliano		0000-0002-9577-5567

ISSN: 2062-5871

eISSN: 2063-5303

Record 100 of 189

Title: Predictors of adverse gambling related consequences among adolescent boys

Author(s): Ricijas, N (Ricijas, Neven); Hundric, DD (Hundric, Dora Dodig); Huic, A (Huic, Aleksandra)

Source: CHILDREN AND YOUTH SERVICES REVIEW **Volume:** 67 **Pages:** 168-176 **DOI:** 10.1016/j.childyouth.2016.06.008 **Published:** AUG 2016

Accession Number: WOS:000381171100020

Author Identifiers:

Author	Web of Science ResearcherID	ORCID Number
Ricijas, Neven	I-7477-2019	0000-0001-8107-8448

ISSN: 0190-7409

eISSN: 1873-7765

Record 101 of 189

Title: The Interaction of Gambling Outcome and Gambling Harm-Minimisation Strategies for Electronic Gambling: the Efficacy of Computer Generated Self-Appraisal Messaging

Author(s): Harris, A (Harris, Andrew); Parke, A (Parke, Adrian)

Source: INTERNATIONAL JOURNAL OF MENTAL HEALTH AND ADDICTION **Volume:** 14 **Issue:** 4 **Pages:** 597-617 **DOI:** 10.1007/s11469-015-9581-y **Published:** AUG 2016

Accession Number: WOS:000380147300019

Author Identifiers:

Author	Web of Science ResearcherID	ORCID Number
Parke, Adrian	K-4354-2015	0000-0002-5242-6308

ISSN: 1557-1874

eISSN: 1557-1882

Record 102 of 189

Title: Preventing and responding to gambling-related harm and crime in the workplace

Author(s): Binde, P (Binde, Per)

Source: NORDIC STUDIES ON ALCOHOL AND DRUGS **Volume:** 33 **Issue:** 3 **Pages:** 247-265 **DOI:** 10.1515/nsad-2016-0020 **Published:** JUL 2016

Accession Number: WOS:000379145900005

Author Identifiers:

Author	Web of Science ResearcherID	ORCID Number
Binde, Per		0000-0002-4875-8115

ISSN: 1455-0725

eISSN: 1458-6126

Record 103 of 189

Title: Harmonizing Screening for Gambling Problems in Epidemiological Surveys - Development of the Rapid Screener for Problem Gambling (RSPG)

Author(s): Challet-Bouju, G (Challet-Bouju, Gaelle); Perrot, B (Perrot, Bastien); Romo, L (Romo, Lucia); Valleur, M (Valleur, Marc); Magalon, D (Magalon, David); Fatseas, M (Fatseas, Melina); Chereau-Boudet, I (Chereau-Boudet, Isabelle); Luquiens, A (Luquiens, Amandine); Grall-Bronnec, M (Grall-Bronnec, Marie); Hardouin, JB (Hardouin, Jean-Benoit)

Group Author(s): Jeu Grp

Source: JOURNAL OF BEHAVIORAL ADDICTIONS **Volume:** 5 **Issue:** 2 **Pages:** 239-250 **DOI:** 10.1556/2006.5.2016.030 **Published:** JUN 2016

Accession Number: WOS:000379339700009

PubMed ID: 27348558

Author Identifiers:

Author	Web of Science ResearcherID	ORCID Number
Romo, Lucia	O-1584-2019	
Luquiens, Amandine	AAB-4984-2019	0000-0002-9402-442X

ISSN: 2062-5871

eISSN: 2063-5303

Record 104 of 189

Title: Video Lottery is the Most Harmful Form of Gambling in Canada

Author(s): MacLaren, VV (MacLaren, Vance Victor)

Source: JOURNAL OF GAMBLING STUDIES **Volume:** 32 **Issue:** 2 **Pages:** 459-485 **DOI:** 10.1007/s10899-015-9560-z **Published:** JUN 2016

Accession Number: WOS:000376598400007

PubMed ID: 26233645

ISSN: 1050-5350

eISSN: 1573-3602

Record 105 of 189

Title: Consumer Perspectives on Gambling Harm Minimisation Measures in an Australian Jurisdiction

Author(s): Jackson, AC (Jackson, Alun C.); Christensen, DR (Christensen, Darren R.); Francis, KL (Francis, Kate L.); Dowling, NA (Dowling, Nicki A.)

Source: JOURNAL OF GAMBLING STUDIES **Volume:** 32 **Issue:** 2 **Pages:** 801-822 **DOI:** 10.1007/s10899-015-9568-4 **Published:** JUN 2016

Accession Number: WOS:000376598400029

PubMed ID: 26440108

Author Identifiers:

Author	Web of Science ResearcherID	ORCID Number
Jackson, Alun	R-6306-2019	0000-0001-9565-1399
Dowling, Nicki		0000-0001-8592-2407
Francis, Kate	K-2679-2015	0000-0002-1751-5313

ISSN: 1050-5350

eISSN: 1573-3602

Record 106 of 189

Title: The Extent and Distribution of Gambling-Related Harms and the Prevention Paradox in a British Population Survey

Author(s): Canale, N (Canale, Natale); Vieno, A (Vieno, Alessio); Griffiths, MD (Griffiths, Mark D.)

Source: JOURNAL OF BEHAVIORAL ADDICTIONS **Volume:** 5 **Issue:** 2 **Pages:** 204-212 **DOI:** 10.1556/2006.5.2016.023 **Published:** JUN 2016

Accession Number: WOS:000379339700005

PubMed ID: 27156382

Author Identifiers:

Author	Web of Science ResearcherID	ORCID Number
Griffiths, Mark		0000-0001-8880-6524
CANALE, NATALE		0000-0002-6032-0490

ISSN: 2062-5871

eISSN: 2063-5303

Record 107 of 189

Title: Players without problem gambling of 55 years and more: events, consequences, and structural and environmental characteristics influencing gambling

Author(s): Giroux, I (Giroux, Isabelle); Ferland, F (Ferland, Francine); Savard, C (Savard, Cathy); Jacques, C (Jacques, Christian); Brochu, P (Brochu, Priscilla); Nadeau, D (Nadeau, Dominic); Landreville, P (Landreville, Philippe); Sevigny, S (Sevigny, Serge)

Source: JOURNAL OF GAMBLING ISSUES **Issue:** 32 **Pages:** 89-110 **DOI:** 10.4309/jgi.2016.32.6 **Published:** MAY 2016

Accession Number: WOS:000409984700006

ISSN: 1910-7595

Record 108 of 189

Title: Gambling in the Czech Republic: Prevalence and Social Consequences

Author(s): Mravcik, V (Mravcik, Viktor); Rous, Z (Rous, Zdenek); Lestinova, ZT (Lestinova, Zuzana Tion); Drbohlavova, B (Drbohlavova, Barbora); Chomynova, P (Chomynova, Pavla); Grohmannova, K (Grohmannova, Kateoina); Janikova, B (Janikova, Barbara); Vlach, T (Vlach, Tomas)

Source: JOURNAL OF BEHAVIORAL ADDICTIONS **Meeting Abstract:** OR-74 **Volume:** 5 **Pages:** 31-31 **Supplement:** 1 **Published:** MAR 2016

Accession Number: WOS:000374534300083

Author Identifiers:

Author	Web of Science ResearcherID	ORCID Number
Drbohlavova, Barbora	C-7663-2017	0000-0001-9723-840X
Chomynova, Pavla	B-3548-2018	0000-0002-4263-3194

ISSN: 2062-5871

eISSN: 2063-5303

Record 109 of 189

Title: Social Responsibility and Harm Minimization in Commercial Gambling in Great Britain

Author(s): Miers, D (Miers, David)

Source: GAMING LAW REVIEW & ECONOMICS-REGULATION COMPLIANCE AND POLICY **Volume:** 20 **Issue:** 2 **Pages:** 164-176 **DOI:** 10.1089/glre.2016.2024 **Published:** MAR 1 2016

Accession Number: WOS:000372932800005

ISSN: 1097-5349

eISSN: 1941-5494

Record 110 of 189

Title: Problem Gambling Among Urban and Rural Gamblers in Limpopo Province, South Africa: Associations with Hazardous and Harmful Alcohol Use and Psychological Distress

Author(s): Skaal, L (Skaal, Linda); Sinclair, H (Sinclair, Heidi); Stein, DJ (Stein, Dan J.); Myers, B (Myers, Bronwyn)

Source: JOURNAL OF GAMBLING STUDIES **Volume:** 32 **Issue:** 1 **Pages:** 217-230 **DOI:** 10.1007/s10899-015-9522-5 **Published:** MAR 2016

Accession Number: WOS:000375403100015

PubMed ID: 25631703

Author Identifiers:

Author	Web of Science ResearcherID	ORCID Number
Stein, Dan J	A-1752-2008	0000-0001-7218-7810

ISSN: 1050-5350

eISSN: 1573-3602

Record 111 of 189

Title: The relationship between player losses and gambling-related harm: evidence from nationally representative cross-sectional surveys in four countries

Author(s): Markham, F (Markham, Francis); Young, M (Young, Martin); Doran, B (Doran, Bruce)

Source: ADDICTION **Volume:** 111 **Issue:** 2 **Pages:** 320-330 **DOI:** 10.1111/add.13178 **Published:** FEB 2016

Accession Number: WOS:000368940500017

PubMed ID: 26567515

Author Identifiers:

Author	Web of Science ResearcherID	ORCID Number
Markham, Francis	H-5471-2019	0000-0002-4266-2569
Doran, Bruce		0000-0002-4214-2205
Young, Martin		0000-0001-5168-9416

ISSN: 0965-2140

eISSN: 1360-0443

Record 112 of 189

Title: Understanding gambling related harm: a proposed definition, conceptual framework, and taxonomy of harms

Author(s): Langham, E (Langham, Erika); Thorne, H (Thorne, Hannah); Browne, M (Browne, Matthew); Donaldson, P (Donaldson, Phillip); Rose, J (Rose, Judy); Rockloff, M (Rockloff, Matthew)

Source: BMC PUBLIC HEALTH **Volume:** 16 **Article Number:** 80 **DOI:** 10.1186/s12889-016-2747-0 **Published:** JAN 27 2016

Accession Number: WOS:000369475600001

PubMed ID: 26818137

Author Identifiers:

Author	Web of Science ResearcherID	ORCID Number

Langham, Erika		0000-0002-1824-5108
Rose, Judy		0000-0001-6383-7666
Browne, Matthew		0000-0002-2668-6229

ISSN: 1471-2458

Record 113 of 189

Title: Dealing with the negative consequences of gambling addiction

Author(s): Blanco Miguel, P (Blanco Miguel, Pilar)

Source: CUADERNOS DE TRABAJO SOCIAL **Volume:** 29 **Issue:** 2 **Pages:** 335-344 **DOI:** 10.5209/CUTS.48858 **Published:** 2016

Accession Number: WOS:000386278700016

ISSN: 0214-0314

eISSN: 1988-8295

Record 114 of 189

Title: The Need for Knowledge Extraction: Understanding Harmful Gambling Behavior with Neural Networks

Author(s): Percy, C (Percy, Chris); Garcez, ASD (Garcez, Artur S. d'Avila); Dragicevic, S (Dragicevic, Simo); Franca, MVM (Franca, Manoel V. M.); Slabaugh, G (Slabaugh, Greg); Weyde, T (Weyde, Tillman)

Edited by: Kaminka GA; Fox M; Bouquet P; Hullermeier E; Dignum V; Dignum F; VanHarmelen F

Source: ECAI 2016: 22ND EUROPEAN CONFERENCE ON ARTIFICIAL INTELLIGENCE **Book Series:** Frontiers in Artificial Intelligence and Applications **Volume:** 285 **Pages:** 974-981 **DOI:** 10.3233/978-1-61499-672-9-974 **Published:** 2016

Accession Number: WOS:000385793700114

Conference Title: 22nd European Conference on Artificial Intelligence (ECAI)

Conference Date: AUG 29-SEP 02, 2016

Conference Location: Hague, NETHERLANDS

Conference Sponsors: European Assoc Artificial Intelligence, PricewaterhouseCoopers, Taylor & Francis Grp, Essence ITN Network, Vrije Univ Amsterdam

Author Identifiers:

Author	Web of Science ResearcherID	ORCID Number
Garcez, Artur		0000-0001-7375-9518
Weyde, Tillman		0000-0001-8028-9905

ISSN: 0922-6389

eISSN: 1879-8314

ISBN: 978-1-61499-672-9; 978-1-61499-671-2

Record 115 of 189

Title: The nature and framing of gambling consequences in advertising

Author(s): Orazi, DC (Orazi, Davide C.); Lei, J (Lei, Jing); Bove, LL (Bove, Liliana L.)

Source: JOURNAL OF BUSINESS RESEARCH **Volume:** 68 **Issue:** 10 **Special Issue:** SI **Pages:** 2049-2056 **DOI:** 10.1016/j.jbusres.2015.03.002 **Published:** OCT 2015

Accession Number: WOS:000360516300002

Author Identifiers:

Author	Web of Science ResearcherID	ORCID Number
Bove, Liliana	J-9658-2014	0000-0002-1201-9281

ISSN: 0148-2963

eISSN: 1873-7978

Record 116 of 189

Title: CPGI-Population Harm: A Supplement to the Canadian Problem Gambling Index

Author(s): Quilty, LC (Quilty, Lena C.); Watson, C (Watson, Chris); Bagby, RM (Bagby, R. Michael)

Source: CANADIAN JOURNAL OF ADDICTION **Volume:** 6 **Issue:** 2 **Pages:** 20-28 **Published:** SEP 2015

Accession Number: WOS:000219284400004

ISSN: 2368-4720

eISSN: 2368-4739

Record 117 of 189

Title: The extent and distribution of gambling harm in Finland as assessed by the Problem Gambling Severity Index

Author(s): Raisamo, SU (Raisamo, Susanna U.); Makela, P (Makela, Pia); Salonen, AH (Salonen, Anne H.); Lintonen, TP (Lintonen, Tomi P.)

Source: EUROPEAN JOURNAL OF PUBLIC HEALTH **Volume:** 25 **Issue:** 4 **Pages:** 716-722 **DOI:** 10.1093/eurpub/cku210 **Published:** AUG 2015

Accession Number: WOS:000359159900034

PubMed ID: 25505020

Author Identifiers:

Author	Web of Science ResearcherID	ORCID Number
Makela, Pia	C-5679-2015	0000-0002-3343-2139
Lintonen, Tomi		0000-0003-3455-2439

ISSN: 1101-1262

eISSN: 1464-360X

Record 118 of 189

Title: Construct Development for the FocaL Adult Gambling Screen (FLAGS): A Risk Measurement for Gambling Harm and Problem Gambling Associated with Electronic Gambling Machines

Author(s): Schellinck, T (Schellinck, Tony); Schrans, T (Schrans, Tracy); Schellinck, H (Schellinck, Heather); Bliemel, M (Bliemel, Michael)

Source: JOURNAL OF GAMBLING ISSUES **Issue:** 30 **Pages:** 140-173 **DOI:** 10.4309/jgi.2015.30.7 **Published:** MAY 2015

Accession Number: WOS:000410418500009

Author Identifiers:

Author	Web of Science ResearcherID	ORCID Number
Bliemel, Michael		0000-0002-7603-6988

ISSN: 1910-7595

Record 119 of 189

Title: The Impulsivity and Sensation-Seeking Mediators of the Psychological Consequences of Pathological Gambling in Adolescence

Author(s): Estevez, A (Estevez, Ana); Herrero-Fernandez, D (Herrero-Fernandez, David); Sarabia, I (Sarabia, Izaskun); Jauregui, P (Jauregui, Paula)

Source: JOURNAL OF GAMBLING STUDIES **Volume:** 31 **Issue:** 1 **Pages:** 91-103 **DOI:** 10.1007/s10899-013-9419-0 **Published:** MAR 2015

Accession Number: WOS:000350221300006

PubMed ID: 24297606

Author Identifiers:

Author	Web of Science ResearcherID	ORCID Number
Estevez, Ana	V-1236-2019	0000-0003-0314-7086
, David	H-6715-2012	0000-0002-6092-8332
Jauregui, Paula	U-6465-2019	0000-0002-9706-0274

ISSN: 1050-5350

eISSN: 1573-3602

Record 120 of 189

Title: Academic-Industry Partnerships in Alcohol and Gambling: a Continuum of Benefits and Harms

Author(s): Stein, DJ (Stein, Dan J.)

Source: ISRAEL JOURNAL OF PSYCHIATRY AND RELATED SCIENCES **Volume:** 52 **Issue:** 2 **Pages:** 81-84 **Published:** 2015

Accession Number: WOS:000375965400002

PubMed ID: 26431410

Author Identifiers:

Author	Web of Science ResearcherID	ORCID Number
Stein, Dan J	A-1752-2008	0000-0001-7218-7810

ISSN: 0333-7308

Record 121 of 189

Title: TAKING CHANCES. ONLINE GAMBLING ADDICTION AND STUDENTS; CAUSES, CONSEQUENCES AND TREATMENT

Author(s): Butler, L (Butler, Lynann Annie)

Edited by: Chova LG; Martinez AL; Torres IC

Source: INTED2015: 9TH INTERNATIONAL TECHNOLOGY, EDUCATION AND DEVELOPMENT CONFERENCE **Book Series:** INTED Proceedings **Pages:** 2891-2894 **Published:** 2015

Accession Number: WOS:000398586302132

Conference Title: 9th International Technology, Education and Development Conference (INTED)

Conference Date: MAR 02-04, 2015

Conference Location: Madrid, SPAIN

ISSN: 2340-1079

ISBN: 978-84-606-5763-7

Record 122 of 189

Title: The influence of harmonious passion on gambling addiction: The moderating effects of proactive coping

Author(s): Shin, HJ (Shin, Hyun Ji); Im, SH (Im, Sook Hee); Kim, KH (Kim, Kyo Heon)

Source: JOURNAL OF BEHAVIORAL ADDICTIONS **Meeting Abstract:** PO-22 **Volume:** 4 **Pages:** 53-54 **Supplement:** 1 **Published:** 2015

Accession Number: WOS:000350801200128

ISSN: 2062-5871

eISSN: 2063-5303

Record 123 of 189

Title: How the causes, consequences and solutions for problem gambling are reported in Australian newspapers: a qualitative content analysis

Author(s): Miller, HE (Miller, Helen E.); Thomas, SL (Thomas, Samantha L.); Robinson, P (Robinson, Priscilla); Daube, M (Daube, Mike)

Source: AUSTRALIAN AND NEW ZEALAND JOURNAL OF PUBLIC HEALTH **Volume:** 38 **Issue:** 6 **Pages:** 529-535 **DOI:** 10.1111/1753-6405.12251 **Published:** DEC 2014

Accession Number: WOS:000345825200009

PubMed ID: 25169775

Author Identifiers:

Author	Web of Science ResearcherID	ORCID Number
Thomas, Samantha		0000-0003-1427-7775
Daube, Mike		0000-0002-3479-2785

ISSN: 1326-0200

eISSN: 1753-6405

Record 124 of 189

Title: FORTUNE OR FOE: THE FATAL HARM CAUSED BY A GAMBLING DISORDER

Author(s): Tse, S (Tse, Samson); Tang, J (Tang, Joe); Wong, P (Wong, Paul)

Source: ADDICTION **Volume:** 109 **Issue:** 12 **Pages:** 2135-2135 **DOI:** 10.1111/add.12744 **Published:** DEC 2014

Accession Number: WOS:000344780300029

PubMed ID: 25384938

Author Identifiers:

Author	Web of Science ResearcherID	ORCID Number
	A-2507-2010	0000-0003-3388-6285
Tse, Samson Shu-Ki	D-4948-2009	0000-0001-9003-1086

ISSN: 0965-2140

eISSN: 1360-0443

Record 125 of 189

Title: Recommendations for International Gambling Harm-Minimisation Guidelines: Comparison with Effective Public Health Policy

Author(s): Gainsbury, SM (Gainsbury, Sally M.); Blankers, M (Blankers, Matthijs); Wilkinson, C (Wilkinson, Claire); Schelleman-Offermans, K (Schelleman-Offermans, Karen); Cousijn, J (Cousijn, Janna)

Source: JOURNAL OF GAMBLING STUDIES **Volume:** 30 **Issue:** 4 **Pages:** 771-788 **DOI:** 10.1007/s10899-013-9389-2 **Published:** DEC 2014

Accession Number: WOS:000344754800001

PubMed ID: 23748884

Author Identifiers:

Author	Web of Science ResearcherID	ORCID Number
Wilkinson, Claire	A-9155-2017	0000-0002-4815-5840
Schelleman-Offermans, Karen	B-3897-2011	0000-0003-3193-0764

ISSN: 1050-5350

eISSN: 1573-3602

Record 126 of 189

Title: Examining the Structural Relationships Among Gambling Motivation, Passion, and Consequences of Internet Sports Betting

Author(s): Lee, CK (Lee, Choong-Ki); Chung, N (Chung, Namho); Bernhard, BJ (Bernhard, Bo J.)

Source: JOURNAL OF GAMBLING STUDIES **Volume:** 30 **Issue:** 4 **Pages:** 845-858 **DOI:** 10.1007/s10899-013-9400-y **Published:** DEC 2014

Accession Number: WOS:000344754800005

PubMed ID: 23824837

Author Identifiers:

Author	Web of Science ResearcherID	ORCID Number
Lee, Choong-Ki	AAH-9113-2020	
Chung, Namho	V-3143-2017	0000-0002-2118-0413

ISSN: 1050-5350

eISSN: 1573-3602

Record 127 of 189

Title: WORKING TOWARDS A HARM INDEX IN PROBLEM GAMBLING: STUDY 2. ARE TREATMENT OUTCOMES DETERMINED BY TYPE OF GAMBLING?

Author(s): Bowden-Jones, H (Bowden-Jones, H.); Ronzitti, S (Ronzitti, S.)

Source: ALCOHOL AND ALCOHOLISM **Meeting Abstract:** SY26-1 **Volume:** 49 **Supplement:** 1 **Published:** SEP 2014

Accession Number: WOS:000342352100269

ISSN: 0735-0414

eISSN: 1464-3502

Record 128 of 189

Title: WORKING TOWARDS A HARM INDEX IN PROBLEM GAMBLING: DOES TYPE OF PLAY AND LEVEL OF INVOLVEMENT DETERMINE SEVERITY OF PRESENTATION? A UK STUDY (HARM INDEX STUDY 1)

Author(s): Bowden-Jones, HM (Bowden-Jones, H. M.); Ronzitti, S (Ronzitti, S.)

Source: ALCOHOL AND ALCOHOLISM **Meeting Abstract:** SY17-3 **Volume:** 49 **Supplement:** 1 **Published:** SEP 2014

Accession Number: WOS:000342352100234

ISSN: 0735-0414

eISSN: 1464-3502

Record 129 of 189

Title: Gambling expenditure predicts harm: evidence from a venue-level study

Author(s): Markham, F (Markham, Francis); Young, M (Young, Martin); Doran, B (Doran, Bruce)

Source: ADDICTION **Volume:** 109 **Issue:** 9 **Pages:** 1509-1516 **DOI:** 10.1111/add.12595 **Published:** SEP 2014

Accession Number: WOS:000340566600019

PubMed ID: 24773526

Author Identifiers:

Author	Web of Science ResearcherID	ORCID Number
Markham, Francis	H-5471-2019	0000-0002-4266-2569
Doran, Bruce		0000-0002-4214-2205
Young, Martin		0000-0001-5168-9416

ISSN: 0965-2140

eISSN: 1360-0443

Record 130 of 189

Title: Gambling Harms and Gambling Help-Seeking Amongst Indigenous Australians

Author(s): Hing, N (Hing, Nerilee); Breen, H (Breen, Helen); Gordon, A (Gordon, Ashley); Russell, A (Russell, Alex)

Source: JOURNAL OF GAMBLING STUDIES **Volume:** 30 **Issue:** 3 **Pages:** 737-755 **DOI:** 10.1007/s10899-013-9388-3 **Published:** SEP 2014

Accession Number: WOS:000340885700013

PubMed ID: 23740348

Author Identifiers:

Author	Web of Science ResearcherID	ORCID Number
Russell, Alex	E-2173-2014	0000-0002-3685-7220
Breen, Helen	Q-7515-2017	0000-0002-1350-6129
Hing, Nerilee		0000-0002-2150-9784

ISSN: 1050-5350

eISSN: 1573-3602

Record 131 of 189

Title: Commentary on Markham et al. (2014): Huffing and puffing our way to accurate gambling-related harm prevalence estimates

Author(s): Hodgins, DC (Hodgins, David C.)

Source: ADDICTION **Volume:** 109 **Issue:** 9 **Pages:** 1517-1517 **DOI:** 10.1111/add.12678 **Published:** SEP 2014

Accession Number: WOS:000340566600020

PubMed ID: 25103103

Author Identifiers:

Author	Web of Science ResearcherID	ORCID Number
Hodgins, David		0000-0003-2737-5200

ISSN: 0965-2140

eISSN: 1360-0443

Record 132 of 189

Title: Exploring the Relationship Between Treatment Satisfaction, Perceived Improvements in Functioning and Well-Being and Gambling Harm Reduction Among Clients of Pathological Gambling Treatment Programs

Author(s): Monnat, SM (Monnat, Shannon M.); Bernhard, B (Bernhard, Bo); Abarbanel, BLL (Abarbanel, Brett L. L.); St John, S (St John, Sarah); Kalina, A (Kalina, Ashlee)

Source: COMMUNITY MENTAL HEALTH JOURNAL **Volume:** 50 **Issue:** 6 **Pages:** 688-696 **DOI:** 10.1007/s10597-013-9635-1 **Published:** AUG 2014

Accession Number: WOS:000339382400008

PubMed ID: 23756725

Author Identifiers:

Author	Web of Science ResearcherID	ORCID Number
Abarbanel, Brett		0000-0002-4279-8466

ISSN: 0010-3853

eISSN: 1573-2789

Record 133 of 189

Title: CROSS-OVER EFFECTS OF ALCOHOL PROTECTIVE BEHAVIORAL STRATEGIES ON GAMBLING CONSEQUENCES AMONG COLLEGE STUDENTS

Author(s): Granato, HF (Granato, H. F.); Paves, A (Paves, A.); Samuelson, M (Samuelson, M.); Larimer, ME (Larimer, M. E.); Lostutter, TW (Lostutter, T. W.); Cronce, JM (Cronce, J. M.)

Source: ALCOHOLISM-CLINICAL AND EXPERIMENTAL RESEARCH **Meeting Abstract:** 1090 **Volume:** 38 **Special Issue:** SI **Pages:** 273A-273A **Supplement:** 1 **Published:** JUN 2014

Accession Number: WOS:000337523701289

Conference Title: 37th Annual Scientific Meeting of the Research-Society-on-Alcoholism (RSA) / 17th Congress of the International-Society-for-Biomedical-Research-on-Alcoholism (ISBRA)

Conference Date: JUN 21-25, 2014

Conference Location: Bellevue, WA

Conference Sponsors: Res Soc Alcoholism, Int Soc Biomed Res Alcoholism

ISSN: 0145-6008

eISSN: 1530-0277

Record 134 of 189

Title: Identifying Indicators of Harmful and Problem Gambling in a Canadian Sample Through Receiver Operating Characteristic Analysis

Author(s): Quilty, LC (Quilty, Lena C.); Murati, DA (Murati, Daniela Avila); Bagby, RM (Bagby, R. Michael)

Source: PSYCHOLOGY OF ADDICTIVE BEHAVIORS **Volume:** 28 **Issue:** 1 **Pages:** 229-237 **DOI:** 10.1037/a0032801 **Published:** MAR 2014

Accession Number: WOS:000334693600024

PubMed ID: 23647158

ISSN: 0893-164X

eISSN: 1939-1501

Record 135 of 189

Title: Household experience of gambling-related harm by socio-economic deprivation in New Zealand: increases in inequality between 2008 and 2012

Author(s): Tu, D (Tu, Danny); Gray, RJ (Gray, Rebecca J.); Walton, DK (Walton, Darren K.)

Source: INTERNATIONAL GAMBLING STUDIES **Volume:** 14 **Issue:** 2 **Pages:** 330-344 **DOI:** 10.1080/14459795.2014.922112 **Published:** 2014

Accession Number: WOS:000343218900010

Author Identifiers:

Author	Web of Science ResearcherID	ORCID Number
Gray, Rebecca		0000-0001-8492-5585

ISSN: 1445-9795

eISSN: 1479-4276

Record 136 of 189

Title: Examining Antecedents and Consequences of Gambling Passion: The Case of Gambling on Horse Races

Author(s): Lee, CK (Lee, Choong-Ki); Back, KJ (Back, Ki-Joon); Hodgins, DC (Hodgins, David C.); Lee, TK (Lee, Tae Kyung)

Source: PSYCHIATRY INVESTIGATION **Volume:** 10 **Issue:** 4 **Pages:** 365-372 **DOI:** 10.4306/pi.2013.10.4.365 **Published:** DEC 2013

Accession Number: WOS:000329599900008

PubMed ID: 24474985

Author Identifiers:

Author	Web of Science ResearcherID	ORCID Number
Lee, Choong-Ki	AAH-9113-2020	
Hodgins, David		0000-0003-2737-5200

ISSN: 1738-3684

eISSN: 1976-3026

Record 137 of 189

Title: Is online gambling harm a fact? Comparing online and offline pathological gambling

Author(s): Hubert, P (Hubert, Pedro); Griffiths, M (Griffiths, Mark); Sommer, M (Sommer, Manuel); De Lourdes, M (De Lourdes, Maria); De Vasconcelos, V (De Vasconcelos, Venancio)

Source: PSYCHOLOGY & HEALTH **Volume:** 28 **Special Issue:** SI **Pages:** 230-230 **Supplement:** 1 **Published:** JUL 1 2013

Accession Number: WOS:000322613800558

ISSN: 0887-0446

Record 138 of 189

Title: From 'morality' policy to 'normal' policy: framing of drug consumption and gambling in Germany and the Netherlands and their regulatory consequences

Author(s): Euchner, EM (Euchner, Eva-Maria); Heichel, S (Heichel, Stephan); Nebel, K (Nebel, Kerstin); Raschzok, A (Raschzok, Andreas)

Source: JOURNAL OF EUROPEAN PUBLIC POLICY **Volume:** 20 **Issue:** 3 **Special Issue:** SI **Pages:** 372-389 **DOI:** 10.1080/13501763.2013.761506 **Published:** MAR 1 2013

Accession Number: WOS:000316334700005

ISSN: 1350-1763

Record 139 of 189

Title: Gambling-Related Harms Among Adolescents: A Population-Based Study

Author(s): Raisamo, S (Raisamo, Susanna); Halme, J (Halme, Jukka); Murto, A (Murto, Antti); Lintonen, T (Lintonen, Tomi)

Source: JOURNAL OF GAMBLING STUDIES **Volume:** 29 **Issue:** 1 **Pages:** 151-159 **DOI:** 10.1007/s10899-012-9298-9 **Published:** MAR 2013

Accession Number: WOS:000315621900012

PubMed ID: 22367512

Author Identifiers:

Author	Web of Science ResearcherID	ORCID Number
Lintonen, Tomi		0000-0003-3455-2439

ISSN: 1050-5350

Record 140 of 189

Title: The Leisure of Women Caring for People Harmfully Involved With Alcohol, Drugs, and Gambling

Author(s): Wood, S (Wood, Stephanie); Tirone, S (Tirone, Susan)

Source: JOURNAL OF LEISURE RESEARCH **Volume:** 45 **Issue:** 5 **Pages:** 583-601 **Published:** 2013

Accession Number: WOS:000326208600002

ISSN: 0022-2216

eISSN: 2159-6417

Record 141 of 189

Title: Modelling vulnerability to gambling related harm: How disadvantage predicts gambling losses

Author(s): Rintoul, AC (Rintoul, Angela C.); Livingstone, C (Livingstone, Charles); Mellor, AP (Mellor, Andrew P.); Jolley, D (Jolley, Damien)

Source: ADDICTION RESEARCH & THEORY **Volume:** 21 **Issue:** 4 **Pages:** 329-338 **DOI:** 10.3109/16066359.2012.727507 **Published:** 2013

Accession Number: WOS:000319946700007

Author Identifiers:

Author	Web of Science ResearcherID	ORCID Number
Rintoul, Angela	E-8359-2011	
Livingstone, Charles		0000-0003-3946-2061
Rintoul, Angela		0000-0003-4159-8814

ISSN: 1606-6359

eISSN: 1476-7392

Record 142 of 189

Title: Harmful alcohol use and frequent use of marijuana among lifetime problem gamblers and the prevalence of cross-addictive behaviour among Greenland Inuit: evidence from the cross-sectional Inuit health in transition Greenland survey 2006-2010

Author(s): Larsen, CVL (Larsen, Christina Viskum Lytken); Curtis, T (Curtis, Tine); Bjerregaard, P (Bjerregaard, Peter)

Source: INTERNATIONAL JOURNAL OF CIRCUMPOLAR HEALTH **Volume:** 72 **Article Number:** 19551 **DOI:** 10.3402/ijch.v72i0.19551 **Published:** 2013

Accession Number: WOS:000317050200001

PubMed ID: 23515920

Author Identifiers:

Author	Web of Science ResearcherID	ORCID Number
Larsen, Christina Viskum Lytken		0000-0002-6245-4222
Bjerregaard, Peter		0000-0001-7153-8447

ISSN: 1239-9736

eISSN: 2242-3982

Record 143 of 189

Title: Can an Industry Be Socially Responsible If Its Products Harm Consumers? The Case of Online Gambling

Author(s): Yani-de-Soriano, M (Yani-de-Soriano, Mirella); Javed, U (Javed, Uzma); Yousafzai, S (Yousafzai, Shumaila)

Source: JOURNAL OF BUSINESS ETHICS **Volume:** 110 **Issue:** 4 **Special Issue:** SI **Pages:** 481-497 **DOI:** 10.1007/s10551-012-1495-z **Published:** NOV 2012

Accession Number: WOS:000314236100008

ISSN: 0167-4544

eISSN: 1573-0697

Record 144 of 189

Title: Bupropion SR and Harm Reduction vs. Abstinence-Focused Treatment for Problem Gambling

Author(s): Desai, N (Desai, Nitigna); Rofman, BE (Rofman, Barbara Elaine); King, K (King, Kendra); O'Connor, A (O'Connor, Ashley); Krebs, C (Krebs, Christopher); Potenza, M (Potenza, Marc); Drebing, C (Drebing, Charles)

Source: AMERICAN JOURNAL ON ADDICTIONS **Meeting Abstract:** 6 **Volume:** 21 **Issue:** 4 **Pages:** 383-384 **Published:** JUL-AUG 2012

Accession Number: WOS:000305122400018

ISSN: 1055-0496

Record 145 of 189

Title: Risk and Protective Factors Associated with Gambling Consequences for Indigenous Australians in North Queensland

Author(s): Breen, HM (Breen, Helen M.)

Source: INTERNATIONAL JOURNAL OF MENTAL HEALTH AND ADDICTION **Volume:** 10 **Issue:** 2 **Pages:** 258-272 **DOI:** 10.1007/s11469-011-9315-8 **Published:** APR 2012

Accession Number: WOS:000310689900010

Author Identifiers:

Author	Web of Science ResearcherID	ORCID Number
Breen, Helen	Q-7515-2017	0000-0002-1350-6129

ISSN: 1557-1874

eISSN: 1557-1882

Record 146 of 189

Title: Gambling harms can be reduced: public health meets politics

Author(s): Livingstone, C (Livingstone, Charles)

Source: HEALTH PROMOTION JOURNAL OF AUSTRALIA **Volume:** 23 **Issue:** 1 **Pages:** 3-3 **Published:** APR 2012

Accession Number: WOS:000303143700001

PubMed ID: 22730948

Author Identifiers:

Author	Web of Science ResearcherID	ORCID Number
Livingstone, Charles		0000-0003-3946-2061

ISSN: 1036-1073

Record 147 of 189

Title: Knowledge, views and experiences of gambling and gambling-related harms in different ethnic and socio-economic groups in New Zealand

Author(s): Walker, SE (Walker, Sue E.); Abbott, MW (Abbott, Max W.); Gray, RJ (Gray, Rebecca J.)

Source: AUSTRALIAN AND NEW ZEALAND JOURNAL OF PUBLIC HEALTH **Volume:** 36 **Issue:** 2 **Pages:** 153-159 **DOI:** 10.1111/j.1753-6405.2012.00847.x **Published:** APR 2012

Accession Number: WOS:000302352600013

PubMed ID: 22487350

Author Identifiers:

Author	Web of Science ResearcherID	ORCID Number
Gray, Rebecca		0000-0001-8492-5585

ISSN: 1326-0200

Record 148 of 189

Title: Placing Bets: gambling venues and the distribution of harm

Author(s): Young, M (Young, Martin); Markham, F (Markham, Francis); Doran, B (Doran, Bruce)

Source: AUSTRALIAN GEOGRAPHER **Volume:** 43 **Issue:** 4 **Pages:** 425-444 **DOI:** 10.1080/00049182.2012.731302 **Published:** 2012

Accession Number: WOS:000312443800007

Author Identifiers:

Author	Web of Science ResearcherID	ORCID Number
Markham, Francis	H-5471-2019	0000-0002-4266-2569
Young, Martin		0000-0001-5168-9416
Doran, Bruce		0000-0002-4214-2205

ISSN: 0004-9182

eISSN: 1465-3311

Record 149 of 189

Title: A Case Study of Gambling Involvement and Its Consequences

Author(s): Hing, N (Hing, Nerilee); Breen, H (Breen, Helen); Gordon, A (Gordon, Ashley)

Source: LEISURE SCIENCES **Volume:** 34 **Issue:** 3 **Pages:** 217-235 **DOI:** 10.1080/01490400.2012.669682 **Published:** 2012

Accession Number: WOS:000304472700002

Author Identifiers:

Author	Web of Science ResearcherID	ORCID Number
Breen, Helen	Q-7515-2017	0000-0002-1350-6129
Hing, Nerilee		0000-0002-2150-9784

ISSN: 0149-0400

eISSN: 1521-0588

Record 150 of 189

Title: Indigenous Gambling Motivations, Behaviour and Consequences in Northern New South Wales, Australia

Author(s): Breen, HM (Breen, Helen M.); Hing, N (Hing, Nerilee); Gordon, A (Gordon, Ashley)

Source: INTERNATIONAL JOURNAL OF MENTAL HEALTH AND ADDICTION **Volume:** 9 **Issue:** 6 **Pages:** 723-739 **DOI:** 10.1007/s11469-010-9293-2 **Published:** DEC 2011

Accession Number: WOS:000310688800010

Author Identifiers:

Author	Web of Science ResearcherID	ORCID Number
Breen, Helen	Q-7515-2017	0000-0002-1350-6129
Hing, Nerilee		0000-0002-2150-9784

ISSN: 1557-1874

eISSN: 1557-1882

Record 151 of 189

Title: Harm promotion: observations on the symbiosis between government and private industries in Australasia for the development of highly accessible gambling markets

Author(s): Livingstone, C (Livingstone, Charles); Adams, PJ (Adams, Peter J.)

Source: ADDICTION **Volume:** 106 **Issue:** 1 **Pages:** 3-8 **DOI:** 10.1111/j.1360-0443.2010.03137.x **Published:** JAN 2011

Accession Number: WOS:000285205000002

PubMed ID: 21188851

Author Identifiers:

Author	Web of Science ResearcherID	ORCID Number
Adams, Peter		0000-0002-3237-0108
Livingstone, Charles		0000-0003-3946-2061

ISSN: 0965-2140

eISSN: 1360-0443

Record 152 of 189

Title: REGULATING HARM - GAMBLING TECHNOLOGY AND THE CHALLENGES FOR GREAT BRITAIN

Author(s): Reith, G (Reith, Gerda)

Source: ADDICTION **Volume:** 106 **Issue:** 1 **Pages:** 9-10 **DOI:** 10.1111/j.1360-0443.2010.03220.x **Published:** JAN 2011

Accession Number: WOS:000285205000003

PubMed ID: 21188852

ISSN: 0965-2140

eISSN: 1360-0443

Record 153 of 189

Title: BEFORE, DURING AND AFTER MEASURES TO REDUCE GAMBLING HARM

Author(s): Ariyabuddhiphongs, V (Ariyabuddhiphongs, Vanchai)

Source: ADDICTION **Volume:** 106 **Issue:** 1 **Pages:** 12-13 **DOI:** 10.1111/j.1360-0443.2010.03178.x **Published:** JAN 2011

Accession Number: WOS:000285205000005

PubMed ID: 21188854

Author Identifiers:

Author	Web of Science ResearcherID	ORCID Number
Hadianfard, Habib	W-5644-2018	0000-0002-1728-632X

ISSN: 0965-2140

Record 154 of 189

Title: Prevalence of Adolescent Problem Gambling, Related Harms and Help-Seeking Behaviours Among an Australian Population

Author(s): Splevins, K (Splevins, Katie); Mireskandari, S (Mireskandari, Shab); Clayton, K (Clayton, Kymbra); Blaszczynski, A (Blaszczynski, Alex)

Source: JOURNAL OF GAMBLING STUDIES **Volume:** 26 **Issue:** 2 **Pages:** 189-204 **DOI:** 10.1007/s10899-009-9169-1 **Published:** JUN 2010

Accession Number: WOS:000277145700002

PubMed ID: 20054622

Author Identifiers:

Author	Web of Science ResearcherID	ORCID Number
Blaszczynski, Alexander	G-2713-2013	0000-0003-1476-0791

ISSN: 1050-5350

Record 155 of 189

Title: PROBLEM GAMBLING: WE SHOULD MEASURE HARM RATHER THAN 'CASES'

Author(s): Blaszczynski, A (Blaszczynski, Alex)

Source: ADDICTION **Volume:** 104 **Issue:** 7 **Pages:** 1072-1074 **DOI:** 10.1111/j.1360-0443.2009.02505.x **Published:** JUL 2009

Accession Number: WOS:000266686000005

PubMed ID: 19563557

Author Identifiers:

Author	Web of Science ResearcherID	ORCID Number
Blaszczynski, Alexander	G-2713-2013	0000-0003-1476-0791

ISSN: 0965-2140

Record 156 of 189

Title: A question of balance: prioritizing public health responses to harm from gambling

Author(s): Adams, PJ (Adams, Peter J.); Raeburn, J (Raeburn, John); de Silva, K (de Silva, Kawshi)

Source: ADDICTION **Volume:** 104 **Issue:** 5 **Pages:** 688-691 **DOI:** 10.1111/j.1360-0443.2008.02414.x **Published:** MAY 2009

Accession Number: WOS:000265008400003

PubMed ID: 19215607

Author Identifiers:

Author	Web of Science ResearcherID	ORCID Number
Adams, Peter		0000-0002-3237-0108

ISSN: 0965-2140

eISSN: 1360-0443

Record 157 of 189

Title: MINIMIZING HARM FROM GAMBLING: WHAT IS THE GAMBLING INDUSTRY'S ROLE?

Author(s): Griffiths, MD (Griffiths, Mark D.)

Source: ADDICTION **Volume:** 104 **Issue:** 5 **Pages:** 696-697 **Published:** MAY 2009

Accession Number: WOS:000265008400007

PubMed ID: 19413782

Author Identifiers:

Author	Web of Science ResearcherID	ORCID Number
Griffiths, Mark		0000-0001-8880-6524

ISSN: 0965-2140

eISSN: 1360-0443

Record 158 of 189

Title: Consequences of Winning: The Role of Gambling Outcomes in the Development of Irrational Beliefs

Author(s): Monaghan, S (Monaghan, Sally); Blaszczynski, A (Blaszczynski, Alex); Nower, L (Nower, Lia)

Source: BEHAVIOURAL AND COGNITIVE PSYCHOTHERAPY **Volume:** 37 **Issue:** 1 **Pages:** 49-59 **DOI:** 10.1017/S135246580800502X **Published:** JAN 2009

Accession Number: WOS:000263394500005

PubMed ID: 19364407

Author Identifiers:

Author	Web of Science ResearcherID	ORCID Number
Blaszczynski, Alexander	G-2713-2013	0000-0003-1476-0791
Nower, Lia	E-7168-2011	0000-0002-2497-8957

ISSN: 1352-4658

Record 159 of 189

Title: Gambling problems in the family - A stratified probability sample study of prevalence and reported consequences

Author(s): Wenzel, HG (Wenzel, Hanne Gro); Oren, A (Oren, Anita); Bakken, IJ (Bakken, Inger Johanne)

Source: BMC PUBLIC HEALTH **Volume:** 8 **Article Number:** 412 **DOI:** 10.1186/1471-2458-8-412 **Published:** DEC 16 2008

Accession Number: WOS:000263271500003

PubMed ID: 19087339

Author Identifiers:

Author	Web of Science ResearcherID	ORCID Number
Bakken, Inger Johanne		0000-0002-4176-8257

ISSN: 1471-2458

Record 160 of 189

Title: The liberalization and (re)regulation of Dutch gambling markets: National consequences of the changing European context

Author(s): Kingma, SF (Kingma, Sytze F.)

Source: REGULATION & GOVERNANCE **Volume:** 2 **Issue:** 4 **Pages:** 445-458 **DOI:** 10.1111/j.1748-5991.2008.00045.x **Published:** DEC 2008

Accession Number: WOS:000261685100005

Author Identifiers:

Author	Web of Science ResearcherID	ORCID Number
Kingma, Sytze	F-8436-2013	

ISSN: 1748-5983

eISSN: 1748-5991

Record 161 of 189

Title: Pathological gambling and its consequences for public health

Author(s): de Oliveira, MPMT (Magalhaes Tavares de Oliveira, Maria Paula); da Silveira, DX (da Silveira, Dartiu Xavier); Silva, MTA (Araujo Silva, Maria Teresa)

Source: REVISTA DE SAUDE PUBLICA **Volume:** 42 **Issue:** 3 **Pages:** 542-549 **Published:** JUN 2008

Accession Number: WOS:000256053300022

PubMed ID: 18461253

Author Identifiers:

Author	Web of Science ResearcherID	ORCID Number
Oliveira, Maria Paula	AAC-8514-2019	

ISSN: 0034-8910

eISSN: 1518-8787

Record 162 of 189

Title: College students' gambling behavior: When does it become harmful?

Author(s): Weinstock, J (Weinstock, Jeremiah); Whelan, JP (Whelan, James P.); Meyers, A (Meyers, Andrew)

Source: JOURNAL OF AMERICAN COLLEGE HEALTH **Volume:** 56 **Issue:** 5 **Pages:** 513-521 **DOI:** 10.3200/JACH.56.5.513-522 **Published:** MAR-APR 2008

Accession Number: WOS:000259692200006

PubMed ID: 18400663

ISSN: 0744-8481

eISSN: 1940-3208

Record 163 of 189

Title: Harm reduction and electronic gambling machines: Does this pair make a happy couple or is divorce foreseen?

Author(s): Cantinotti, M (Cantinotti, Michael); Ladouceur, R (Ladouceur, Robert)

Source: JOURNAL OF GAMBLING STUDIES **Volume:** 24 **Issue:** 1 **Pages:** 39-54 **DOI:** 10.1007/s10899-007-9072-6 **Published:** MAR 2008

Accession Number: WOS:000252157300004

PubMed ID: 17674162

ISSN: 1050-5350

eISSN: 1573-3602

Record 164 of 189

Title: Virtual harm reduction efforts for Internet gambling: effects of deposit limits on actual Internet sports gambling behavior

Author(s): Broda, A (Broda, Anja); LaPlante, DA (LaPlante, Debi A.); Nelson, SE (Nelson, Sarah E.); LaBrie, RA (LaBrie, Richard A.); Bosworth, LB (Bosworth, Leslie B.); Shaffer, HJ (Shaffer, Howard J.)

Source: HARM REDUCTION JOURNAL **Volume:** 5 **Article Number:** 27 **DOI:** 10.1186/1477-7517-5-27 **Published:** 2008

Accession Number: WOS:000207448600027

PubMed ID: 18684323

ISSN: 1477-7517

Record 165 of 189

Title: Preventing the Incidence and Harm of Gambling Problems

Author(s): Dickson-Gillespie, L (Dickson-Gillespie, Laurie); Rugle, L (Rugle, Lori); Rosenthal, R (Rosenthal, Richard); Fong, T (Fong, Timothy)

Source: JOURNAL OF PRIMARY PREVENTION **Volume:** 29 **Issue:** 1 **Pages:** 37-55 **DOI:** 10.1007/s10935-008-0126-z **Published:** JAN 2008

Accession Number: WOS:000207647500003

PubMed ID: 18373202

Author Identifiers:

Author	Web of Science ResearcherID	ORCID Number
Rosenthal, Richard J.	AAG-6689-2019	

ISSN: 0278-095X

Record 166 of 189

Title: Gambling as an addictive behaviour: impaired control, harm minimization, treatment and prevention

Author(s): Lim, D (Lim, Dominic)

Source: AUSTRALIAN AND NEW ZEALAND JOURNAL OF PSYCHIATRY **Volume:** 41 **Issue:** 6 **Pages:** 558-559 **Published:** JUN 2007

Accession Number: WOS:000247622200015

ISSN: 0004-8674

Record 167 of 189

Title: Association between posttreatment gambling behavior and harm in pathological gamblers

Author(s): Weinstock, J (Weinstock, Jeremiah); Ledgerwood, DM (Ledgerwood, David M.); Petry, NM (Petry, Nancy M.)

Source: PSYCHOLOGY OF ADDICTIVE BEHAVIORS **Volume:** 21 **Issue:** 2 **Pages:** 185-193 **DOI:** 10.1037/0893-164X.21.2.185 **Published:** JUN 2007

Accession Number: WOS:000247222500007

PubMed ID: 17563138

ISSN: 0893-164X

eISSN: 1939-1501

Record 168 of 189

Title: Gambling as an addictive behaviour: Impaired control, harm minimisation, treatment and prevention

Author(s): West, R (West, Robert)

Source: ADDICTION **Volume:** 102 **Issue:** 3 **Pages:** 492-493 **DOI:** 10.1111/j.1360-0443.2007.01785.x **Published:** MAR 2007

Accession Number: WOS:000244098000023

ISSN: 0965-2140

Record 169 of 189

Title: Gambling as an addictive behaviour: Impaired control, harm minimisation, treatment and prevention

Author(s): George, S (George, Sanju)

Source: BRITISH JOURNAL OF PSYCHIATRY **Volume:** 190 **Pages:** 181-182 **DOI:** 10.1192/bjp.bp.106.026187 **Published:** FEB 2007

Accession Number: WOS:000244411200030

ISSN: 0007-1250

eISSN: 1472-1465

Record 170 of 189

Title: Gambling as an addictive behaviour. Impaired control, harm minimisation, treatment and prevention.

Author(s): Paul, EM (Paul, Edward M.)

Source: JOURNAL OF ADDICTIVE DISEASES **Volume:** 26 **Issue:** 1 **Pages:** 87-88 **Published:** 2007

Accession Number: WOS:000245291600013

ISSN: 1055-0887

Record 171 of 189

Title: Evidence against prospect theories in gambles with positive, negative, and mixed consequences

Author(s): Birnbaum, MH (Birnbaum, Michael H.)

Source: JOURNAL OF ECONOMIC PSYCHOLOGY **Volume:** 27 **Issue:** 6 **Pages:** 737-761 **DOI:** 10.1016/j.joep.2006.04.001 **Published:** DEC 2006

Accession Number: WOS:000243178700002

ISSN: 0167-4870

Record 172 of 189

Title: Risk of harm among gamblers in the general population as a function of level of participation in gambling activities

Author(s): Currie, SR (Currie, SR); Hodgins, DC (Hodgins, DC); Wang, JL (Wang, JL); el-Guebaly, N (el-Guebaly, N); Wynne, H (Wynne, H); Chen, S (Chen, S)

Source: ADDICTION **Volume:** 101 **Issue:** 4 **Pages:** 570-580 **DOI:** 10.1111/j.1360-0443.2006.01392.x **Published:** APR 2006

Accession Number: WOS:000235936400016

PubMed ID: 16548936

Author Identifiers:

Author	Web of Science ResearcherID	ORCID Number
Hodgins, David		0000-0003-2737-5200

ISSN: 0965-2140

eISSN: 1360-0443

Record 173 of 189

Title: Structural changes to electronic gaming machines as effective harm minimization strategies for non-problem and problem gamblers

Author(s): Sharpe, L (Sharpe, Louise); Walker, M (Walker, Michael); Coughlan, MJ (Coughlan, Maree-Jo); Enersen, K (Enersen, Kirsten); Blaszczynski, A (Blaszczynski, Alex)

Source: JOURNAL OF GAMBLING STUDIES **Volume:** 21 **Issue:** 4 **Pages:** 503-520 **DOI:** 10.1007/s10899-005-5560-8 **Published:** DEC 2005

Accession Number: WOS:000202951400007

PubMed ID: 16311879

Author Identifiers:

Author	Web of Science ResearcherID	ORCID Number
Blaszczynski, Alexander	G-2713-2013	0000-0003-1476-0791
Sharpe, Louise	J-1868-2012	
Sharpe, Louise		0000-0002-8790-6272

ISSN: 1050-5350

eISSN: 1573-3602

Record 174 of 189

Title: Passion and gambling: Investigating the divergent affective and cognitive consequences of gambling

Author(s): Mageau, GA (Mageau, GA); Vallerand, RJ (Vallerand, RJ); Rousseau, FL (Rousseau, FL); Ratelle, CF (Ratelle, CF); Provencher, PJ (Provencher, PJ)

Source: JOURNAL OF APPLIED SOCIAL PSYCHOLOGY **Volume:** 35 **Issue:** 1 **Pages:** 100-118 **DOI:** 10.1111/j.1559-1816.2005.tb02095.x **Published:** JAN 2005

Accession Number: WOS:000226921900006

Author Identifiers:

Author	Web of Science ResearcherID	ORCID Number
Vallerand, Robert J	B-3028-2013	
Mageau, Genevieve A.	E-5499-2014	
Vallerand, Robert		0000-0001-5852-8877
Ratelle, Catherine		0000-0002-4789-9274

ISSN: 0021-9029

eISSN: 1559-1816

Record 175 of 189

Title: An investigation of the myopia for future consequences theory of vmf patient behaviour on the Iowa gambling task: An abstract neural network simulation

Author(s): Kalidindi, K (Kalidindi, K); Bowman, H (Bowman, H); Wyble, B (Wyble, B)

Edited by: Cangelosi A; Bugmann G; Borisyuk R

Source: MODELING LANGUAGE, COGNITION AND ACTION **Book Series:** Progress in Neural Processing **Volume:** 16 **Pages:** 331-335 **DOI:** 10.1142/9789812701886_0034 **Published:** 2005

Accession Number: WOS:000230933300034

Conference Title: 9th Neural Computational and Psychology Workshop

Conference Date: SEP 08-10, 2004

Conference Location: Univ Plymouth, Plymouth, ENGLAND

Conference Host: Univ Plymouth

Author Identifiers:

Author	Web of Science ResearcherID	ORCID Number
Borisyuk, Roman	A-7476-2014	0000-0003-1384-9057

ISBN: 981-256-324-5

Record 176 of 189

Title: Betting your life on it - Gambling harms both health and equality

Author(s): Wilson, N (Wilson, N)

Source: BRITISH MEDICAL JOURNAL **Volume:** 329 **Issue:** 7479 **Pages:** 1405-1405 **DOI:** 10.1136/bmj.329.7479.1405-a **Published:** DEC 11 2004

Accession Number: WOS:000225785000042

PubMed ID: 15591582

ISSN: 0959-535X

Record 177 of 189

Title: Youth gambling problems: A harm reduction prevention model

Author(s): Dickson, L (Dickson, L); Derevensky, JL (Derevensky, JL); Gupta, R (Gupta, R)

Source: ADDICTION RESEARCH & THEORY **Volume:** 12 **Issue:** 4 **Pages:** 305-316 **DOI:** 10.1080/1606635042000236466 **Published:** AUG 2004

Accession Number: WOS:000223763300002

ISSN: 1606-6359

eISSN: 1476-7392

Record 178 of 189

Title: On the shoulders of Merton - Potentially sobering consequences of problem gambling policy

Author(s): Bernhard, BJ (Bernhard, BJ); Preston, FW (Preston, FW)

Source: AMERICAN BEHAVIORAL SCIENTIST **Volume:** 47 **Issue:** 11 **Pages:** 1395-1405 **DOI:** 10.1177/0002764204265340 **Published:** JUL 2004

Accession Number: WOS:000221901900003

ISSN: 0002-7642

eISSN: 1552-3381

Record 179 of 189

Title: Harm reduction for the prevention of youth gambling problems: Lessons learned from adolescent high-risk behavior prevention programs

Author(s): Dickson, LM (Dickson, LM); Derevensky, JL (Derevensky, JL); Gupta, R (Gupta, R)

Source: JOURNAL OF ADOLESCENT RESEARCH **Volume:** 19 **Issue:** 2 **Pages:** 233-263 **DOI:** 10.1177/0743558403258272 **Published:** MAR 2004

Accession Number: WOS:000188796700006

ISSN: 0743-5584

eISSN: 1552-6895

Record 180 of 189

Title: Direct versus indirect emotional consequences on the Iowa Gambling Task

Author(s): Turnbull, OH (Turnbull, OH); Berry, H (Berry, H); Bowman, CH (Bowman, CH)

Source: BRAIN AND COGNITION **Volume:** 53 **Issue:** 2 **Pages:** 389-392 **DOI:** 10.1016/S0278-2626(03)00151-9 **Published:** NOV 2003

Accession Number: WOS:000186643700103

PubMed ID: 14607188

Conference Title: 13th Annual Meeting on Tennet: Theoretical and Experimental Neuropsychology

Conference Date: JUN 20, 2002-JUN 22, 2003

Conference Location: MONTREAL, CANADA

Conference Sponsors: Univ Quebec Montreal, Min Rech Sci & Technol Quebec, Fonds Rech Sante Quebec, Canada Hlth Res Inst

ISSN: 0278-2626

Record 181 of 189

Title: Gambling and psychotropic substance consumption; prevalence, coexistence and consequences

Author(s): Arseneault, L (Arseneault, L); Ladouceur, R (Ladouceur, R); Vitaro, F (Vitaro, F)

Source: CANADIAN PSYCHOLOGY-PSYCHOLOGIE CANADIENNE **Volume:** 42 **Issue:** 3 **Pages:** 173-184 **DOI:** 10.1037/h0086890 **Published:** AUG 2001

Accession Number: WOS:000170472100002

ISSN: 0708-5591

Record 182 of 189

Title: Too much too young - Focus - Few drugs are tested on children, so doctors are having to gamble on suitable doses - often with dangerous consequences

Author(s): Fricker, J (Fricker, J)

Source: NEW SCIENTIST **Volume:** 161 **Issue:** 2174 **Pages:** 18-19 **Published:** FEB 20 1999

Accession Number: WOS:000078842000018

ISSN: 0262-4079

Record 183 of 189

Title: The luck business: The devastating consequences and broken promises of America's gambling explosion

Author(s): Green, MT (Green, MT)

Source: JOURNAL OF POLICY ANALYSIS AND MANAGEMENT **Volume:** 17 **Issue:** 2 **Pages:** 358-362 **Published:** SPR 1998

Accession Number: WOS:000072727200023

ISSN: 0276-8739

Record 184 of 189

Title: GAMBLERS IN THE GARDEN, THE POLITICAL CONSEQUENCES OF THE FIX

Author(s): FABIAN, A (FABIAN, A)

Source: SOUTH ATLANTIC QUARTERLY **Volume:** 95 **Issue:** 2 **Pages:** 501-521 **Published:** SPR 1996

Accession Number: WOS:A1996UU75600010

ISSN: 0038-2876

Record 185 of 189

Title: THE LUCK BUSINESS - THE DEVASTATING CONSEQUENCES AND BROKEN PROMISES OF AMERICAS GAMBLING EXPLOSION - GOODMAN,R

Author(s): ALVAREZ, A (ALVAREZ, A)

Source: NEW YORK REVIEW OF BOOKS **Volume:** 43 **Issue:** 1 **Pages:** 15-& **Published:** JAN 11 1996

Accession Number: WOS:A1996TL61400003

ISSN: 0028-7504

Record 186 of 189

Title: INSIGHTS ABOUT PATHOLOGICAL GAMBLERS - CHASING LOSSES IN SPITE OF THE CONSEQUENCES

Author(s): CUSACK, JR (CUSACK, JR); MALANEY, KR (MALANEY, KR); DEPRY, DL (DEPRY, DL)

Source: POSTGRADUATE MEDICINE **Volume:** 93 **Issue:** 5 **Pages:** 169-& **Published:** APR 1993

Accession Number: WOS:A1993KW82900010

PubMed ID: 8460075

ISSN: 0032-5481

Record 187 of 189

Title: GAMBLING WITH NATURE - A NEW PARADIGM OF NATURE AND ITS CONSEQUENCES FOR NATURE MANAGEMENT STRATEGY

Author(s): VANZOEST, J (VANZOEST, J)

Edited by: CARTER RWG; CURTIS TG; SHEEHYSKEFFINGTON MJ

Source: COASTAL DUNES : GEOMORPHOLOGY, ECOLOGY AND MANAGEMENT FOR CONSERVATION **Pages:** 503-515 **Published:** 1992

Accession Number: WOS:A1992BW21P00046

Conference Title: 3RD EUROPEAN CONGRESS ON COASTAL DUNES

Conference Date: JUN 17-21, 1992

Conference Location: UNIV COLL GALWAY, GALWAY, IRELAND

Conference Sponsors: EUROPEAN UNION COASTAL CONSERVAT

Conference Host: UNIV COLL GALWAY

ISBN: 90-5410-058-3

Record 188 of 189

Title: LEADERS, RIVERBOAT GAMBLERS, OR PURPOSEFUL UNINTENDED CONSEQUENCES IN THE MANAGEMENT OF COMPLEX, DANGEROUS TECHNOLOGIES

Author(s): OSBORN, RN (OSBORN, RN); JACKSON, DH (JACKSON, DH)

Source: ACADEMY OF MANAGEMENT JOURNAL **Volume:** 31 **Issue:** 4 **Pages:** 924-947 **DOI:** 10.2307/256345 **Published:** DEC 1988

Accession Number: WOS:A1988R138200008

ISSN: 0001-4273

Record 189 of 189

Title: IMPACT OF LEGALIZED GAMBLING - SOCIOECONOMIC CONSEQUENCES OF LOTTERIES AND OFF-TRACK BETTING - WEINSTEIN,D AND DEITCH,L

Author(s): MARTINEZ, T (MARTINEZ, T); LANDSBERG, M (LANDSBERG, M)

Source: SOCIETY **Volume:** 13 **Issue:** 1 **Pages:** 86-87 **Published:** 1975

Accession Number: WOS:A1975BD61700014

ISSN: 0147-2011
