# Supplementary material for: Is there a health inequality in gambling related harms? A systematic review
Source: BMC Public Health. 2021 Feb 6;21:305. doi: 10.1186/s12889-021-10337-3 (PMC7866763; doi:10.1186/s12889-021-10337-3)
Supplement: Supplementary file 3 — Additional file 3. Table of Quality Checks. Table of the quality assessment completed by researchers as a PDF [file 12889_2021_10337_MOESM3_ESM.pdf]

Study	Criteria																
	1	2	3	4	5	6	7	8	9	10	11	12	13	14			
	Question / Objective sufficiently described	Study design evident and appropriate	Method of subject/comparison group selection or source of information/input variables described and appropriate	Subject (and comparison group, if applicable) characteristics sufficiently described	If interventional and random allocation was possible, was it described	f interventional and blinding of investigators was possible, was it reported	If interventional and blinding of subjects was possible, was it reported	Outcome and (if applicable) exposure measure(s) well defined and robust to measurement / misclassification bias? Means of assessment reported	Sample size appropriate	Analytic methods described/justified and appropriate	Some estimate of variance is reported for the main results	Controlled for confounding	Results reported in sufficient detail	Conclusions supported by the results	Total Sum	Total Possible Sum	Summary Score
Angus et al. (2019)	3	3	3	3	N/A	N/A	N/A	3	3	3	3	3	3	3	22	22	**1.00**
Apinuntavech et. al. (2012)	3	3	3	3	N/A	N/A	N/A	2	3	2	1	1	2	3	15	22	**0.68**
Browne and Rockloff (2018)	3	3	3	3	N/A	N/A	N/A	3	2	3	3	1	3	3	19	22	**0.86**
Browne et al. (2017)	3	3	3	3	N/A	N/A	N/A	3	3	3	2	1	3	3	19	22	**0.86**
Browne et al. (2019)	3	3	3	3	N/A	N/A	N/A	3	3	3	3	3	3	3	22	22	**1.00**
Browne et al. (2020)	3	3	3	3	N/A	N/A	N/A	3	3	3	1	3	3	3	20	22	**0.91**
Browne, Goodwin, and Rockloff (2018)	3	3	3	3	N/A	N/A	N/A	3	N/A	3	3	3	3	3	20	20	**1.00**
Canale, Vieno, and Griffiths (2016)	3	3	3	3	N/A	N/A	N/A	3	3	3	3	2	3	3	21	22	**0.95**
Castren et al. (2018)	3	3	2	3	N/A	N/A	N/A	3	3	3	3	2	3	3	20	22	**0.91**
Currie et al. (2006)	3	3	3	2	N/A	N/A	N/A	3	3	3	3	2	3	3	20	22	**0.91**
Delfabbro, Georgiou, and King (2020)	3	3	3	3	N/A	N/A	N/A	3	3	3	3	3	3	3	22	22	**1.00**
Estevez et al. (2015)	3	3	2	3	N/A	N/A	N/A	3	3	3	3	3	3	3	21	22	**0.95**
Hing et al. (2014)	3	3	3	3	N/A	N/A	N/A	3	3	3	2	1	3	3	19	22	**0.86**
Hubert and Griffiths (2018)	3	3	2	3	N/A	N/A	N/A	3	3	3	3	N/A	3	3	19	20	**0.95**
Jeffrey et al. (2019)	3	3	3	2	N/A	N/A	N/A	3	3	3	3	3	3	3	21	22	**0.95**
Kildahl et. al. (2020)	2	3	3	3	N/A	N/A	N/A	3	3	3	3	3	3	3	21	22	**0.95**
Langham et al. (2017)	3	3	3	3	N/A	N/A	N/A	3	3	3	3	3	3	3	22	22	**1.00**
Larsen, Curtis, and Bjerregaard (2013)	3	3	3	3	N/A	N/A	N/A	3	3	3	3	3	3	3	22	22	**1.00**
Lee, Chung, and Bernhard (2014)	3	3	2	3	N/A	N/A	N/A	3	3	3	3	3	3	3	21	22	**0.95**

Li et al. (2017)	3	3	3	2	N/A	N/A	N/A	3	3	3	3	1	3	3	19	22	**0.86**
Livazovic and Bojcic (2019)	3	3	3	3	N/A	N/A	N/A	3	3	3	3	1	3	3	20	22	**0.91**
Lloyd et al. (2016)	3	3	2	3	N/A	N/A	N/A	3	3	3	3	3	3	3	21	22	**0.95**
Mageau et al. (2005)	3	3	3	2	N/A	N/A	N/A	3	3	3	3	3	3	3	21	22	**0.95**
May-Chahal et al. (2017)	3	3	3	2	N/A	N/A	N/A	2	3	3	3	3	3	3	20	22	**0.91**
Melendez-Torres et al. (2019)	3	3	3	3	N/A	N/A	N/A	3	3	3	3	1	3	3	20	22	**0.91**
Mihaylova, Kairouz, and Nadeau (2013)	3	3	3	3	N/A	N/A	N/A	3	3	3	3	3	3	3	22	22	**1.00**
Raisamo et al. (2013)	3	3	3	3	N/A	N/A	N/A	3	3	3	3	2	3	3	21	22	**0.95**
Raisamo et al. (2015)	3	3	3	3	N/A	N/A	N/A	3	3	3	3	3	3	3	22	22	**1.00**
Raisamo et al. (2019)	3	3	3	3	N/A	N/A	N/A	3	3	3	3	1	3	3	20	22	**0.91**
Ricijas, Hundric, and Huic (2016)	3	3	2	3	N/A	N/A	N/A	3	3	3	3	3	3	3	21	22	**0.95**
Salonen, Alho, and Castren (2017)	3	3	3	3	N/A	N/A	N/A	3	3	3	3	3	3	3	22	22	**1.00**
Salonen et al. (2018)	3	3	2	2	N/A	N/A	N/A	2	3	3	3	1	3	3	17	22	**0.77**
Shannon, Anjoul, and Blaszczynski (2017)	3	3	2	3	N/A	N/A	N/A	3	3	3	3	N/A	3	3	19	20	**0.95**
Skaal et al. (2016)	3	3	2	3	N/A	N/A	N/A	3	3	3	3	3	3	3	21	22	**0.95**
Splevins et al. (2010)	3	3	2	3	N/A	N/A	N/A	3	2	3	3	1	3	3	18	22	**0.82**
Tu, Gray, and Walton (2014)	3	3	3	3	N/A	N/A	N/A	2	3	3	3	1	3	3	19	22	**0.86**
Yani-de-Soriano, Javed, and Yousafzai [illegible]	3	3	2	2	N/A	N/A	N/A	3	2	3	3	1	3	3	17	22	**0.77**

Study	Criteria												
	1	2	3	4	5	6	7	8	9	10			
	Question / objective sufficiently described	Study design evident and appropriate	Context for the study clear	Connection to a theoretical framework / wider body of knowledge	Sampling strategy described, relevant and justified	Data collection methods clearly described and systematic	Data analysis clearly described and systematic	Use of verification procedure(s) to establish credibility	Conclusions supported by the results	Reflexivity of the account	Total Sum	Total Possible Sum	Summary Score
Anderson, Rempusheski, and Leedy (2018)	3	3	3	3	3	3	3	3	3	2	19	28	**0.68**
Bergh and Kuhlhorn (1994)	3	3	3	3	1	2	1	1	3	1	11	28	**0.39**
Binde (2016)	3	3	3	3	1	2	2	1	3	1	12	28	**0.43**
Bramley, Norrie, and Manthorpe (2019)	3	3	3	3	2	3	3	1	3	1	15	28	**0.54**
Bramley, Norrie, and Manthorpe (2020)	3	3	3	3	3	3	3	1	3	1	16	28	**0.57**

Breen (2012)	3	3	3	3	3	3	3	3	3	1	18	28	**0.64**
Breen, Hing, and Gordon (2011)	3	3	3	3	3	3	3	3	3	1	18	28	**0.64**
Delfabbro and King (2019)	2	2	3	3	1	1	2	1	3	2	10	28	**0.36**
Ferrara, Franceschini, and Corsello (2018)	1	2	3	3	1	1	2	1	3	1	8	28	**0.29**
Fulton (2019)	3	3	3	3	3	3	3	3	3	1	18	28	**0.64**
Goh, Ng, and Yeoh (2016)	3	3	3	3	3	3	3	3	3	3	20	28	**0.71**
Heiskanen and Matilainen (2020)	3	3	3	3	3	3	3	3	3	1	18	28	**0.64**
Hing and Breen (2015)	3	3	3	3	3	3	2	1	3	3	17	28	**0.61**
Hing, Breen, and Gordon (2012)	3	3	3	3	3	3	3	3	3	3	20	28	**0.71**
Kolandai-Matchett et al. (2017)	3	3	3	3	3	3	3	2	3	1	17	28	**0.61**
Langham et al. (2016)	3	3	3	3	3	3	3	3	3	2	19	28	**0.68**
McCarthy et al. (2019)	3	2	3	3	2	2	2	1	3	2	13	28	**0.46**
Paterson, Whitty, and Leslie (2020)	3	3	3	3	3	3	3	1	3	1	16	28	**0.57**
Pitt et al. (2017)	3	3	3	3	2	3	3	3	3	3	19	28	**0.68**
Rintoul, Deblaquiere, and Thomas (2017)	3	3	3	3	2	3	3	3	3	2	18	28	**0.64**
Samuelsson, Sundqvist, and Binde (2018)	3	3	3	3	3	3	3	3	3	2	19	28	**0.68**
Wardle et al. (2019)	3	3	3	3	3	3	3	3	3	2	19	28	**0.68**
