# Supplementary material for: Is there a health inequality in gambling related harms? A systematic review
Source: BMC Public Health. 2021 Feb 6;21:305. doi: 10.1186/s12889-021-10337-3 (PMC7866763; doi:10.1186/s12889-021-10337-3)
Supplement: Supplementary file 4 — Additional file 4. Table of Extracted Data. Full table of data extracted from included studies as a PDF [file 12889_2021_10337_MOESM4_ESM.pdf]

Author(s)	Study Design	Measures Used	Country	Sample	Funding	Discussion Categories	Relevant Results Identified
Anderson, Rempusheski, and Leedy (2018)	Qualitative – Interview	In-Depth Interview	USA	34<br>Seniors (62-88)	University of Delaware	Age	Seniors gambling let to mild-severe arguments, broken relationships (divorce), anxiety, debts, exhausting pensions/ inheritance/ savings, shame
Angus et al. (2019)	Case-Control Study	Harm Questionnaire<br>PGSI	Australia	99 Clinical<br>330 Non-Clinical	The New South Wales Government from the Responsible Gambling Fund	Clinical	100% Psych harms in clinical compared to 14.85% in non-clinical, and 97.98% financial harm vs. 23.33% in non-clinical. Greater severity of harm in all domains for clinical
						Risk Severity	PGs associated with more severe harms, more PGs in clinical sample, 100% psych harms in clinical PGs vs. 54.69% in non-clinical PGs
Apinuntavech et. al. (2012)	Cross-Sectional	DSM-IV-TR	Thailand	173 Students	Psychiatric Association of Thailand	SES	Average GPA lower in gamblers, high chance of smoking, alcohol use and energy drink use, over 50% report 10/20 harms, psych effects were most common, guilt, concealing behaviour, perceived poor health, depression, insomnia, debts, selling possessions, anxiety, substance use, school absence, considering suicide
Bergh and Kuhlhorn (1994)	Qualitative – Interview	Interview<br>DSM-III-R	Sweden	105 Gen. Pop.	The Commission for Social Research	Age	Younger (20-34) gambled longer than older (35+)
						Gender	Similar results for all harms
						Gambling Behaviour	Amount spent correlates with debt
Binde (2016)	Qualitative – Narrative Review	Interview	Sweden	1 Security Professional<br>2 Addiction Professionals<br>5 Therapists	Small writing grant from The Responsible Gambling Trust UK	General Gamblers Harm - Workplace Harms	Gambling in breaks, gambling during work, borrowing from colleagues, poor work performance, lateness, depression, anxiety, tiredness, irritability, absences, less talk about other hobbies (preoccupation), selling belongings, appearing troubled, tax

				5 Problem Gamblers	Government or Public Health funding		authorities investigating wages, poor self-care, poor standards of belongings (car), crime – transaction irregularities/ embezzlement
Bramley, Norrie, and Manthorpe (2019)	Qualitative – Interview	Semi-Structured Interview	UK	23 Medical and Care Professionals	Ridgeway Information Ltd.	Clinical	Once gambling became habit in a patient they showed high anxiety, financial difficulties, depression,
Bramley, Norrie, & Manthorpe (2020)	Qualitative – Focus Group	Focus Group	UK	32 Migrants	London School of Hygiene and Tropical Medicine and King's College London Interdisciplinary Research Fund and the NIHR Policy Research Programme	Culture	Migrants experienced similar gambling harms to the general population including selling possessions, relationship breakdown, mental health problems, drug use or drug selling, homelessness, domestic violence, sex work and suicide. Participants felt harms were exacerbated by factors including lack of a 'safety net', difficulty accessing informal support. Sub-Saharan African migrants (particularly men) felt that if they lost money they would feel a loss of community status, other migrants felt that they could not easily undo financial hardship as additional employment would have family consequences.
Breen (2012)	Qualitative – Interview	Interview	Australia	60 Indigenous<br>14 Non-Indigenous<br>14 Counsellors<br>20 Venue Managers	Not Stated	Age	Card harms increased by starting young and skipping school. Young gamblers had reduced lifelong aspirations
						Culture	Indigenous Australians experienced debt, child neglect (missed school), lack of resources (food, clothes), social isolation, shame, reluctant to seek help, community shame (gossip)
Breen, Hing, and Gordon (2011)	Qualitative - Interview	Interview<br>Observations	Australia	169 Indigenous Australians	Gambling Research Australia	Game Choice	Card and commercial gambling associated with poverty. Card games led to financial loss and loss of welfare. Commercial led to financial hardship, family and relationship issues, mental health issues, crime, eviction, homelessness, domestic violence, neglect, relationship breakdown, depression, suicidality, theft, sold belongings

Browne and Rockloff (2018)	Secondary Data Analysis	CSPG<br>PGSI<br>SOGS-4<br>DSM-IV-16<br>BBGS-2<br>72-Item Checklist<br>CQLS	Multiple	156 Adult Gamblers	Victorian Responsible Gambling Foundation	Risk Severity	Only 10% of report financial harms were in PG but over 50% social deviance harms in PG, domestic violence, neglect, lowered religious connection most common in RG, other domains mostly even distribution, common moderate harms such as increased debt and less spending on essentials more likely in LR, over 50% selling belongings were RG. Severe harms like loss of utilities or significant assets more likely MR or PG. Most severe harms (unhygienic living) mostly PG.
Browne, Goodwin, and Rockloff (2018)	Cross-Sectional	CSPG<br>PGSI<br>SOGS-4<br>DSM-IV-16<br>BBGS-2<br>72-Item Checklist<br>CQLS	Worldwide	156 Adult Gamblers	Victorian Responsible Gambling Fund	Age/ Gender	Non-significant difference
						SES	Wellbeing difference between $15-30k AUD and $101-150k AUD less than 5 points
						Risk Severity	Prevalence of harm in Non-PG twice that of PG (41.5 vs 20.6)
Browne et al. (2019)	Cross-Sectional	GOES<br>PGSI<br>GFM<br>SGP<br>SGHS	Canada	121 Gen. Pop.	Alberta Gambling Research Institute	Age	Younger ages had a large correlation but was insignificant
						Gender	No significant bivariate relationship to harm
						SES	Part time work and unemployment, marriage status, lower education, and lower income had large correlations but were insignificant
						Gambling Behaviour	Motivation didn't predict harm
Browne et al. (2017)	Cross-Sectional	PGSI<br>72-Item Checklist	Australia	786 Gamblers	Not Stated	Risk Severity	Highest severity of risk showed the same disability weights as moderate to severe alcohol use disorder and similar weights to bipolar disorder. Moderate risk group had similar weights to mild alcohol use disorder and stroke. Low risk was estimated as slightly less disability weights than anxiety.

							Less severe harms experienced by a large proportion of the population might be of more magnitude than the intense harms by the small high risk proportion. Low risk individuals were 5-10x more prevalent than high risk.
Browne et al. (2020)	Secondary Data Analysis	PPGM<br>72-Item Checklist	Finland	3795 Gen. Pop.	The Ministry of Social Affairs and Health, Finland	Risk Severity	Total weighted harms reported in the highest PPGM category was 282 compared to 395 across the three lower categories. All individuals in highest risk category reported at least 1 harm, however any individual reporting 1+ harm is more likely to be in a lower risk group. Highest risk group make up the fewest participants reporting few (1-4) harms, and overwhelming majority reporting many (20+) harms.<br>Mild financial, psychological and work/study harms were broadly distributed across all PPGM groups. Mild financial and psych harms were common. Health, relationship and social deviance harms were mostly restricted to highest risk category (over 50% of reports). Mild harms were the most prevalent and were more common in gamblers not experiencing impaired control.<br>Severe financial harms were more likely in those displaying control issues. In psych harms more than 50% of all harms were reported in the lower risk categories, and more than 50% of severe harms were in the high risk group. Work/study and social deviance harms had low prevalence overall, health harms and relationship harms both had around 50% of all harm from the high risk group.
Canale, Vieno, and Griffiths (2016)	Secondary Data Analysis	DSM-IV	UK	781 Gen. Pop.	None	Age	Dependence and social harm higher in 16-34 than 35-54 and 55+

		Frequency Survey<br>PGSI<br>4-Point Scale				Gender	Males gambled more frequently for longer and spent more. Dependence and social harm higher in men
						Gambling Behaviour	Most harms reported by non-high time and spend regular gamblers. Harm odds increased with greater gambling volume individually. So higher individual risk in high volume play but larger proportion of at least one harm reported in low volume play.
Castren et al. (2018)	Secondary Data Analysis	PGSI<br>SOGS	Finland	360 Gen. Pop.	The Ministry of Social Affairs and Health, Helsinki	Age	Participants over 24 experienced fewer harms
						Gender	Gender did not have an effect on gambling harms
						Gambling Behaviour	Daily play doubled harms, multiple gambling sessions per week also increased harms. Spending at least 1% of monthly income increased harms.
						Game Choice / Online vs. Offline	Online gambling statistically associated with harm but not strongly. Scratchers, betting, slots, non-poker games online, poke online and non-monopoly increased harms. Weekly lottery, fast-paced lottery, low-paced lottery, casino games, horses and private gambling were not included. Weekly lottery had lowest harms and non-monopoly the highest.
Currie et al. (2006)	Secondary Data Analysis	CPGI<br>DSM-IV<br>NODS	Canada	202 Gen. Pop.	Alberta Gaming Research Institute	Age	Under 40s more likely two or more harms than over 40s
						Gender	Two or more harms in the last year more likely reported by men
						SES	Two or more harms in the last year more likely reported by low income, high school or lower education
						Culture	Two or more harms in the last year more likely reported by non-caucasians

						Gambling Behaviour/ Game Choice	Harm risk increased with frequency of play on EGMs, instant-win tickets, bingo and casino games but not lottery plays
Delfabbro and King (2019)	Qualitative – Narrative Review	DSM-5<br>PGSI<br>SOGS<br>NODS<br>VGS<br>PPGM<br>GES	Multiple	N/A	None	Risk Severity	Not much harm appearing in LR when scaling severity. LR-MR show only low-medium severity harms but a large proportion of these lower severity harms present<br>Even when scaling severity financial harms found in LR groups (i.e. selling belongings)<br>Low SES in the non-PG/LR group may mean financial harms aren't gambling related (many non-PG scored 0 so little-no gambling)
Delfabbro, Georgiou, and King (2020)	Cross-Sectional	Gambling Activity Scale<br>Risk-Behaviour Scale<br>PGSI<br>72-Item Checklist	Multiple	554 Adult Gamblers	Not Stated	Risk Severity	Participants scored as "problem gamblers" experienced more harm in general, harm reported by low risk groups was very small when participants were asked to indicate if harm resulted from gambling. Harm close to 0 for all but financial and psych harms in the lowest risk group.
Estevez et al. (2015)	Case-Control Study	Multicage-CAD-4<br>Symptom List-90<br>AISS	Spain	131 Clinical<br>2 Non-Clinical	International Contest ONCE of Research on Responsible Gambling	Clinical	Young adults in the clinical sample had more dysfunctional symptomatology: anxiety, depression, hostility, OC behaviour, and somatisation, high scores for alcohol addiction, drug addiction, game "addiction", sex "addiction" and "compulsive" shopping.<br>No significant difference for eating or internet use.<br>Impulsivity partially mediated anxiety, depression and psychoticism and perfectly mediated somatization, OCD, interpersonal sensitivity, paranoid ideation and hostility.
	Qualitative – Narrative Review	Multiple – Study dependent	Worldwide	Adolescents	None	Age	Young age was associated with high rates of addiction comorbidity

Ferrara, Franceschini, and Corsello (2018)						Gender	Males were associated with high rates of addiction comorbidity
						Culture	Non-white was associated with high rates of addiction comorbidity
						Risk Severity	Participants rated as high risk were more likely to have increased alcoholism or substance use, depression, dysthymia, anxiety, panic and phobia, anger, resentment, headaches, and gastrointestinal symptoms, eating disorders, and criminality.<br>Poor relationship communication, conflicts and arguments, consideration of separation or divorce. Anger and conflict in families, less independence, less engagement in intellectual or cultural activities, little expression of emotions.
						Game Choice	Sports betting was associated with high rates of addiction comorbidity
Fulton (2019)	Qualitative – Interview	In-Depth Interview	Ireland	22 Recovering Gamblers and their Social Circle	Irish Research Council of Ireland's Research Project Grants and The Social Protection Research Innovation Awards	Online vs. Offline	Hiding debt, stealing, hidden bills leading to further debt. Use of online gambling reduced shame but the double life led to stress and emotional deterioration, mood swings, irritability, relationship issues, arguments, self-perception changes (low-esteem increased shame).<br>One individual showed changing perception of reality as he stole from work but did not process that this was theft.
Goh, Ng, and Yeoh (2016)	Qualitative – Interview	In-Depth Interview	Singapore	105 FEO Applicants	National Council on Problem Gambling Singapore and The Humanities and Social Sciences Research Grant	Age	Verbal abuse was most common from young male gamblers towards their parents. Older males were found to coerce money from their wives.
						Gender	Physical abuse was equally common between genders

						Culture	Where traditional gender roles were present a mother gambling was seen to lead to child neglect, followed by father leaving work to care for the children and therefore causing financial hardships. The gambler was seen as self-centred and family relationships became strained.
Heiskanen and Matilainen (2020)	Qualitative – Focus Group	Focus Group	Finland	25 “Baby Boomers”	The Finnish Foundation for Alcohol Studies	Age	Difficulty passing a machine without gambling, excessive time and money spent both online and offline, loss of money, negative consequences to social relationships.<br>Some participants discussed feeling unable to “meddle”, and shame if seen gambling publicly (particular in small country communities) suggesting a person with a gambling problem would be less likely to have peer support
Hing et al. (2014)	Cross-Sectional	PGSI<br>11-Listed Harms	Australia	1259 Indigenous Australians	Australian Research Council	Culture	The three most prevalent harms were betting more than they could afford to lose (49%), guilt or regret (44%), and chasing loss (44%). These were followed by financial problems (38%), feeling they had a problem (36%), criticism (34%), needing to spend more (34%), borrowing or selling (25%) and health problems (25%).
						Risk Severity	Among problem gamblers 93.8% indicated at least sometimes betting more than they could afford to lose, 92.9% reported betting larger amounts to get the same thrill and 96.2% reported loss chasing. A significantly higher proportion of moderate risk gamblers (18%) experienced family arguments than low risk gamblers (0.9%). 96.2% of problem gamblers were aware of having an issue, 94.9% had a gambling related health issue

Hing and Breen (2015)	Qualitative – Interview	Semi-Structured Interview	Australia	169 Indigenous Australians	Gambling Research Australia	Culture	Counsellors noted that cultural acceptance was high for gambling and so those with a problem had a strong support network. However when discussing harms isolation from community was a key factor, with family and relationship difficulties. Harms included financial and personal distress, debt, cut off utilities, crime, loss of employment, homelessness, lack of food and no support. Non-Indigenous counsellors highlighted credit card debt as a key harm in the community, using one card to pay off another and getting into a cycle of loss chasing. Gamblers hid their losses due to shame, low self-esteem and guilt and these were highlighted as reasons help wasn't sought out. In contrast a win would lead to high popularity and community power.<br>Family issues were described as missing key events, neglecting children, lying and arguments, violence and breakups. These relationship issues sometimes led to social isolation. Within Indigenous communities' solitary gamblers were viewed as poor role models and were believed to have lost their cultural values. Community gamblers were seen as those with positive consequences and strong family groups.
Hubert and Griffiths (2018)	Cohort Study	Portuguese SOGS<br>Self-Devised Likert Scale of Harms	Portugal	1599 Gen. Pop.	None	Online vs. Offline	Offline gamblers were more likely to have jobs, children and a stable relationship/ Online gamblers appear to become problem gamblers faster (30y/o compared to 40y/o). They had fewer suicidal thoughts but less control over impulsivity and less able to cope with frustrations. Same level of online and offline suicide attempts. Online gambling associated with heavy alcohol use, poor academic functioning, unemployment, and less money later in life.
			Australia			Gender/ Culture	Heavy commercial gamblers who were women in small villages and men in towns

							both experienced similar harms. Heavy card players of both genders experienced similar harms. Binge commercial gamblers were mostly men and experienced financial loss.
Hing, Breen, and Gordon (2012)	Qualitative – Interview	Semi-Structured Interview		169 Indigenous Australians	Gambling Research Australia	Game Choice	Heavy card players spent their pension and borrowed money, as well as playing all day/night. Heavy commercial gamblers played alone, spending their whole pay, and played all day/night. They experienced debt, relationship issues, lost homes, overcrowded refuge housing, missed bills, lack of food and poor nutrition, child neglect and abuse, stress, lying, domestic violence, self-esteem issues, depression suicidality, theft, selling items, and ripping off their work. Binge commercial gamblers had financial loss.
Jeffrey et al. (2019)	Cohort Study	PGSI<br>72-Item Checklist<br>83-Item Checklist	Australia and New Zealand	5036 Gamblers	Victorian Responsible Gambling Foundation and New Zealand Ministry of Health	General Gamblers Harm - Gamblers Perceptions vs. Others	Gamblers were more likely to report individual problems such as not having access to money, being late, gambling during work or study time, alcohol use, attempted suicide, hygiene issues, lack of sleep, reduced physical activity, shame, and worthlessness. Spouses reported joint harms such as missed bills, tension and conflict in the relationship. Gamblers may be less aware of relationship dysfunction.
Kildahl et. al. (2020)	Cross-Sectional	GamTest<br>IOWA Task	Denmark	140 Gamblers	The Luxembourg National Research Fund	Gambling Behaviour	Overconsumption of time, overconsumption of money, negative consequences of overconsumption of money, negative social consequences, negative emotional consequences and general consequences all increase linearly with gambling frequency.<br>Individuals influenced by reward frequency, who were more likely to swap decks rather than persevere with the same cards were more likely to overconsume time, experience negative social consequences, emotional consequences and overconsume money.

Kolandai-Matchett et al. (2017)	Mixed Methods	Focus Group	New Zealand	8 Gamblers<br>26 Professionals	New Zealand Ministry of Health	Culture	Pacific people experienced harms that are similar to other populations. However contexts were complex when culture-related. Collectivist values and least distinction between family, friends and strangers. Collectivism meant harm is far-reaching and included loss of belongings, isolation, shame, loss of respect, disruption of trusting relationships, transference of communal responsibility and an overall loss of social cohesion. Non-present or non-contributing members may be excluded by the wider community.
Langham et al. (2017)	Secondary Data Analysis	SOC-3 Scale<br>PGSI<br>72-Item Checklist	Australia	1236	Victorian Responsible Gambling Foundation	Sense of Coherence	Sense of coherence correlated with all harm categories. Stronger sense meant fewer harms. Weaker sense specifically related to reduced spending on beneficial items and essentials, increased negative health behaviour such as loss of sleep, reduced physical activity, poor nutrition, stress related illness and depression, feelings of failure, worthlessness, hopelessness, shame, anger, and the need to run away.<br>Weak sense of coherence was not linked to attempted suicide.
Langham et al. (2016)	Qualitative – Multiple Methods	Focus Group<br>Interview	Australia	35	Victorian Responsible Gambling Foundation	General Gamblers Harm/ Risk Severity	Cultural and relationship harms were separate however occurring together due to the link between family and culture. Financial harms triggered a behaviour change or reassessment of circumstances. The crisis threshold was found to depend on tolerance for deprivation. Relationship harm was dependent on the other persons opinion of gambling, and there was a category of distortion such as when a child becomes the parents' caretaker. Emotional harms were most effected by other harm categories, with shame as the most pervasive harm. Health harm occurred for even recreational gamblers but were not well documented or measured. Criminality was only associated

							with high risk / problem gamblers and was usually a second-order harm used as a tool to address primary harms such as financial.
Larsen, Curtis, and Bjerregaard (2013)	Cross-Sectional	Lie/Bet Questionnaire<br>Interview	Greenland	2189	None	Age	Alcohol use increased with age in PG but decreased with age in non-PG.
						Risk Severity	Harmful alcohol use and frequent marijuana use was associated with lifetime gambling problems and prevalence was high amount PG compared to non-PG.
Lee, Chung, and Bernhard (2014)	Cross-Sectional	Gambling Passion Scale<br>Consequences Checklist	South Korea	288 Online Gamblers	Not Stated	Gambling Behaviour	Motivations of excitement, escape and challenge were linked to positive consequences, whereas money motivation led to harms and obsession.
						Online vs. Offline	Obsessive online gambling was associated with anxiety and guilt.
Li et al. (2017)	Cohort Study	72-Item Checklist<br>PGSI	Australia	5205	Victorian Responsible Gambling Foundation	General Gamblers Harm	Harms in all domains tended to accumulate more quickly to gamblers than to 'affected others' as gambling problems increased.
						Risk Severity	Reduced spending was the most prevalent financial harm, reduced performance, reduced sleep, worry, shame, reduced time, and paying money were highest in other categories. Sold items, absence, reduced performance, reduced sleep, extreme distress, and tension had the highest correlation with PGSI categories.
Livazovic and Bojcic (2019)	Cross-Sectional	CAGI<br>GPSS	Croatia	366 Students	None	Age	Older participants scored higher on problem gambling and risk behaviour but there was no increase in harms.
						Gender	Males scored significantly higher on psychological, social and financial harms but also higher for problem gambling and risk behaviour.

						SES	Family life and parents' education level had no significant effect on harms. Lower achievers in school reported higher psychological harms. There was no significant differences in harms across school types
						Risk Severity	There was a weak significant correlation between success in school and risk score.
Lloyd et al. (2016)	Cross-Sectional	DSM-IV<br>Frequency Questions<br>Mood Disorder Q<br>General Health Q<br>Self-Harm Yes/No<br>CAGE<br>PRIME-MD<br>10-Item Drug Screen	Europe and North America	4125	Responsibility in Gambling Trust and the Economic and Social Research Council	Age	Age was not a significant predictor for thoughts or acts of self-harm
						Gender	Gambling related thoughts of self-harm were more frequent in males. This contrasts with non-gambling thoughts of self-harm. Actual acts of self-harm were not related to gender.
						SES	Gambling related thoughts of self-harm were more frequent in the unemployed but not linked to marital status. Gambling related acts of self-harm were also associated with unemployment, but not marriage status. Thoughts of harm also related to parents gambling.
						Gambling Behaviour	Thoughts of harm were associated with years gambling<br>Gambling for mood modification and money was associated with thoughts of self-harm. Gambling for enjoyment was negatively associated. Non-gambling related self-harm was also significantly related to gambling for mood modification and inversely with gambling for enjoyment.

Mageau et al. (2005)	Cross-Sectional	Gambling Passion Scale<br>Questionnaire	Canada	554	Not Stated	Gambling Behaviour	Harmonious passion (someone choosing to gamble) was related to positive emotions and thoughts, and negatively related to feeling judged or guilty. However after engaging in gambling it no longer related to guilt. Harmonious passion was not related to anxiety. Obsessive passion (feeling compelled to gamble) was strongly associated with guilt, anxiety, and negative emotions. Obsession was negatively related to feeling in control and having fun.
						Game Choice	Casino users reported more positive and negative outcomes overall than lottery players.
May-Chahal et al. (2017)	Cross-Sectional	PGSI<br>Substance Use Q<br>UK National Offenders Data	UK	1057 Prisoners	Economic and Social Research Council	Criminality	The prevalence of gambling within prison was higher than a general population but prevalence of gambling before incarceration was significantly lower. High rate mid-20s peak offenders were 5.3x more likely to chase losses and experience harms. Rate chronic persisters were 3.7x more likely. There was no significant link between gambling and drug use, but occasional gamblers were more likely to abstain from alcohol and drugs.
						Risk Severity	Nearly 2/3 who scored highly on the PGSI abstained from drugs and alcohol.
McCarthy et al. (2019)	Qualitative – Narrative Review	Multiple	Worldwide	Women	None	Age/ Game Choice	Older women personally believed EGMs were less harmful as they could socialise
						Gender	Women were more likely to report comorbid anxiety and depression than men. Also personality disorders, alcoholism and psychological distress.
						Culture	Women from ethnic minorities and indigenous communities (Māori, Aboriginal

							and Pacific New Zealanders) were more at risk of harms compared to European women.<br>Some women in Southeast Asia only saw positive consequences despite negative ones being recorded as present.
Melendez-Torres et al. (2019)	Secondary Data Analysis	Student Health Wellbeing Survey	Wales	363 Students	British Heart Foundation Cancer Research, Economic & Social Research and Medical Research, Welsh Government & Welcome Trust	Age	Increasing school year was associated with increasing socioemotional harms and increased gambling. Harms increased with age more acutely in males.
						Gender	Women were less likely to feel bad about gambling. Harms increased with age was more acute in males.
						SES	Higher family affluence was associated with more harms but also more gambling. Feeling less school connectedness/ belonging led to more harms and also more gambling.
						Culture	Non-white British students experienced more harms. White female students were less likely to feel bad about gambling.
Mihaylova, Kairouz, and Nadeau (2013)	Cohort Study	CPGI – PGSI<br>AUDIT<br>Drug Frequency Q	Canada	2139 Undergraduate Students	Centre Dollard-Cormier, University Institute on Addictions	Game Choice	Online poker gamblers were more likely to gamble at least weekly, spend more and have higher annual debt as a result of poker specifically.
						Online vs. Offline	Online poker players had a greater risk of alcohol dependency. Cannabis use did not differ between online and offline gamblers but online poker users were more likely to have used other illicit drugs over the past year. Academic performance did not differ between online and offline gamblers bur online poker players were more likely to report family issues, studying issues and financial issues.
Paterson, Whitty, and Leslie (2020)	Qualitative – Systematic Review	Multiple	UK<br>Australia	Military Personnel	None	Military Personnel	Those in the military were found to be quickly reprimanded for gambling but meaningful assistance was slow to be

			New Zealand<br>Canada<br>USA				offered. In contrast one study found 21/25 active-duty gamblers who received treatment were retained in the military. Several studies highlighted comorbid substance abuse, mental health problems and suicide in military personnel. One study found 9/35 gamblers receiving treatment had major depressive disorder. One study found 20% of gamblers receiving treatment endorsed suicide ideation and 3 had made attempts. The study noted that targeting the gambling issue led to no further ideation.
Pitt et al. (2017)	Qualitative - Interview	Semi-Structured Interview	Australia	48 Children (8-16)	Australian Research Council Discovery Grant on Sports Wagering	Age	Children understood the slim chance of winning and that continuing to play meant you would lose “until you’re poor.” Children gambled with pocket money or verbal agreements and so financial harms were not experienced. Children didn’t seem to perceive harms other than peer pressure and financial loss, though other studies cited show exposure as a child led to increased alcoholism and cigarette use. Children were found to be more likely to experience harm later in life due to believing sport bets were skill based and gambling as a child with minimal harms due to family protection.
Raisamo et al. (2013)	Secondary Data Analysis	Adolescent Health and Lifestyle Survey<br>Harms Questions	Finland	4566	Finnish Foundation for Alcohol Studies and the Ministry of Social Affairs and Health	Age	Gambling involvement increased with age, though harms differed very little. Despite this financial issues appeared much less common in adolescents.
						Gender	Reported harms differed very little between genders. However when not examining frequent gamblers males reported more financial issues than females.
						Gambling Behaviour	Considerably more frequent gamblers reported harms than occasional gamblers. The most common harm for frequent gamblers was guilt or shame, followed by relationship issues and daily routine

							disruptions. Daily gamblers were significantly more likely to report all harms.
Raisamo et al. (2015)	Secondary Data Analysis	PGSI<br>Frequency of Play<br>Spends	Finland	4484	Ministry of Social Affairs and Health Helsinki	Age	Guilt was not related to age. Younger ages were more likely to report harms.
						Gender	Men had a higher prevalence of harm but gambled more and spent more. Increased spends, betting above means and guilt were most prevalent harms. When controlling for frequency and spends gender was no longer related to harms.
						Risk Severity	The most harm reported were found in low-medium risk scorers.
						Gambling Behaviour	Higher frequency of play and higher spends caused more harms. Harm risk increased when weekly spends exceeded E21.
Raisamo et al. (2019)	Secondary Data Analysis	6-Month Gambling<br>Spends<br>8-Item Harm List	Finland	18857 Adolescents	Ministry of Social Affairs and Health Helsinki	Gender	Most prevalent harm was guilt or shame for both genders. Disrupted daily rhythm and disrupted schoolwork were second most common in girls but conflict with friends was second for boys.
Ricijas, Hundric, and Huic (2016)	Cross-Sectional	Gambling Activities, Motives and Beliefs<br>CAGI<br>Delinquency Scale<br>Experiences Measure	Croatia	Male Students	Not Stated	Risk Severity	Half of the participants were social gamblers without consequences. Occasional gamblers had low-moderate consequences and problem/high risk gamblers had serious consequences. Higher risk score associated with delinquency, and cognitive distortions.
						Game Choice	Sports bettors, VLT users and virtual bettors showed severe psychosocial consequences related to gambling.
Rintoul, Deblaquiere, and Thomas (2017)	Qualitative – Multiple Methods	Observation<br>Semi-Structured Interview	Australia	60	Commonwealth Government of Australia	General Gamblers Harm	Venues highlighted inappropriate social behaviour and emotional responses such as aggression, shouting at the machine or people, appearing sad or depressed, being withdrawn or sweating excessively. In 34

		Focus Groups					hours harms were present at all observed venues.
						Gambling Behaviour	Gambling fast and intensely, betting over $3 per spin, using multiple machines at once, skipping meals and withdrawing cash multiple times led to more observed harms.
Salonen, Alho, and Castren (2017)	Secondary Data Analysis	PGSI<br>SOGS<br>Attitudes towards Gambling	Finland	4515	Ministry of Social Affairs and Health Helsinki	Age	Females aged 18-24 experienced more harms but also reported an increase in occasional gambling. Gambling more than intended was most common for all age groups.
						Gender	Gambling prevalence was higher in males however females 18-24 reported experiencing more harms. Loss of control (gambling more than intended) and guilt were the most common harms for both genders. The portion of women who lost control, felt guilty, and hid betting slips increased while harms such as lying about wins and loss chasing decreased in males.
Salonen et al. (2018)	Secondary Data Analysis	Frequency and Habit Questions<br>14-Item PPGM<br>72-Item Checklist	Finland	7305	Ministry of Social Affairs and Health - Appropriation of the Lotteries Act	Age	Financial harm, work and study harm, health problems and emotional harms all tended to decline in older age groups. Relationship issues did not differ. Financial harm was the most commonly reported among younger ages.
						Gender	Men reported more harms than women. Most typical harms reported were financial or emotional/psychological. Men reported more work and study harms than women.
						Clinical	11% Gen. Pop experienced at least one harm during 2016. The clinical sample experienced a notably larger amount of harm 88% emotional, 87% financial, 87% health and 81% relationship. Financial harms, health harms, and work/study harms were

							similar in both samples, clinical showed more anger in emotional harms than Gen. Pop. Overall other harms were rare in Gen. Pop but in clinical promising to pay back money without intending to, stealing and outcast feelings were common.
Samuelsson, Sundqvist, and Binde (2018)	Qualitative – Interview	Semi-Structured Interview<br>PGSI	Sweden	40	Public Health Agency of Sweden	Gambling Behaviour	Low frequency stable gamblers (mild-no harm) had only shame and guilt. High frequency gamblers with decreasing use (moderate harm) had substantial financial loss, frustration, alcohol use and isolation. Periodic gamblers (moderate harm) showed financial, psychological, insomnia, isolation, relationship issues and low self-esteem. High frequency increased users (substantial harm) had irrational thoughts (felt immortal), and increased spends.<br>Harms such as financial issues and psychological distress caused a period of reduced play depending upon individual life changes and support networks.
Shannon, Anjoul, and Blaszczynski (2017)	Case-Control Study	PGSI<br>48-Harm Indicators	Australia	542	South Australian Independent Gambling Authority	Clinical	In the clinical sample the highest rated harms were reduced savings, doing without, worry, frustration and debt. The lower rated harms were drug use, suicide, bankruptcy, self-injury, and education problems. In the general community debt, partner issues, feeling constrained, going out less, decreased self-control and lower pride were rated worst. The distribution of averaged harm was consistent across both samples, excluding reduced savings and decreased happiness.
Skaal et al. (2016)	Cross-Sectional	PGSI<br>10-KPDS<br>AUDIT	South Africa	900	South African National Responsible Gambling Foundation	SES	Participants from urban areas were more likely to report psychological distress and be high risk gamblers.<br>Alcohol use was most strongly associated with high risk gambling in individuals from urban areas

						Risk Severity	High risk gambling was associated with alcohol use and psychological distress.
Splevins et al. (2010)	Cross-Sectional	DSM-IV-MR-J<br>SOGS<br>Harm Scale<br>Attitudes	Australia	252 Students	Star City Casino	Age	Students spent pocket money or wages from part-time jobs on gambling. There was no significant difference between self-report and peer-reported signs of a gambling problem suggesting adolescents may be less aware of what to look for.
						Gender	Males were more likely to be preoccupied with thoughts of gambling, feel the need to increase spending, and experience arguments.
						Risk Severity	Highest scoring individuals on the SOGS were more likely to miss school, sell personal property, commit illegal acts, and try other risks like smoking and drugs. Legal drugs and ecstasy use was the same for all risk categories.
Tu, Gray, and Walton (2014)	Secondary Data Analysis	Health Lifestyle Survey<br>New Zealand Index of Deprivation	New Zealand	6020	Ministry of Health	SES	More affluent individuals were more likely to gamble than less affluent, however where rates of gambling and also harm dropped in affluent groups they did not in less affluent.
Wardle et al. (2019)	Qualitative – Systematic Rapid Review	Multiple	Worldwide	Migrants	King's College London and London School of Tropical Hygiene and Medicine Interdisciplinary Research Fund	Culture	Migrants were less likely to gamble than non-migrants but were found to be more likely to experience harms. There was minimal evidence of specific harms migrants experience and whether these differed from non-migrants. However they found that Spanish migrants tended to spend over 300 euros daily, claim losses as wins, and chase losses. Australian migrants experienced financial harm, shame, relationship issues, suicide, mental health issues, isolation and prostitution.

Yani-de-Soriano, Javed, and Yousafzai (2012)	Mixed Methods	SOGS<br>K-10 Scale<br>Dichotomous Health Survey<br>8-Internet Addiction Scale	UK	209 Students	Cardiff Business School	Risk Severity	Probable pathological gamblers reported the highest degree of harms in all categories, including longest time spent online generally and for gambling specifically. As risk scores increased so did physical, mental health, social relationship and academic harms.
						Online vs. Offline	Online gambling was associated with binge drinking but not smoking. Around 60% of participants who were online gamblers rated as high risk (problem gambler) and as scores increased so did physical, mental health, social relationship and academic harms.
